# Frequency-dependent coupling in response to oscillatory inputs in minimal networks of electrically coupled nodes: Gap junction networks and spatially extended neurons

**DOI:** 10.1007/s00422-026-01047-3

**Published:** 2026-07-23

**Authors:** Andrea Bel, Ulises Chialva, Horacio G. Rotstein

**Affiliations:** 1 Departamento de Matemática, Universidad Nacional del Sur (UNS) and CONICET, Bahía Blanca, Argentina; 2 Federated Department of Biological Sciences, New Jersey Institute of Technology and Rutgers University, Newark, NJ USA; 3 Present Address: ETSI de Minas y Energía, Departamento de Ingeniería Geológica y Minera, Universidad Politécnica de Madrid, Madrid, España

## Abstract

In electrically coupled networks, the coupling coefficient (CC) quantifies the strength of the connectivity between pairs of nodes. The CC is typically measured by computing the relative stationary responses to constant inputs of the indirectly activated (post-J) and the directly activated (pre-J) nodes. The natural extension of the CC to time-dependent inputs is frequency-dependent and has two components reflecting the contributions of the amplitude and phase frequency-dependent profiles (curves of these quantities as a function of the frequency f) of the participating nodes: the quotient of amplitudes K(f) and the phase-difference $\Delta \Phi (f)$ profiles. The properties and mechanisms of generation of these frequency-dependent CCs (FD-CCs) are largely unknown beyond electrically coupled passive cells and their electrical linear circuit equivalents. For passive cells, K(f) is monotonically decreasing (low-pass filter) and $\Delta \Phi (f)$ is monotonically increasing and positive. Moreover, for linear systems, the FD-CCs depend on the properties of the post-J cell and the connectivity and are independent of the properties of the pre-J cell and the input amplitude. It remains largely unclear how the FD-CCs are shaped by the presence of (i) intrinsic cellular positive and negative feedback currents (resonance and amplification), and (ii) cellular nonlinearities that incorporates the dependence of the FD-CC on the post-J node in addition to the pre-J one. In this paper we address these issues by using biophysically plausible (conductance-based) mathematical modeling, numerical simulations, analytical calculations and dynamical systems tools. We conduct a systematic analysis of the properties of the FD-CC profiles in networks of two electrically connected nodes receiving oscillatory inputs, which is the minimal network architecture that allows for a systematic study of the biophysical and dynamic mechanisms that shape the FD-CC profiles. The participating neurons are either passive cells (low-pass filters) or resonators (band-pass filter) and exhibit lagging or mixed leading-lagging phase responses as the input frequency increases. The formalism and tools we develop and use in this paper are amenable to be extended to larger networks with an arbitrary number of nodes, to spatially extended multicompartment neuronal models, and to neurons having a variety of ionic currents.

## Introduction

Neuronal circuits are impacted by external inputs that often have oscillatory components spanning a range of frequencies (f). The frequency-dependent outputs are shaped by the complex interaction between the input and the cellular and synaptic properties. Neuronal filters describe the information processing building blocks where specific frequency components of the output are enhanced over others (Hutcheon and Yarom 2000; Tsodyks et al. 2000; Izhikevich et al. 2003; Stark et al. 2022; Laudansky et al. 2014; Akam and Kullmann 2012). Therefore neuronal filters play a crucial role in understanding neuronal communication and how the brain process information, generates rhythmic activity and performs complex computations (Maass 2016; Beiran and Ostojic 2019; Fortune and Rose 2001; Klyachko and Stevens 2006; Thomson 2003; Akam and Kullmann 2010; Stark et al. 2022; Blankenburg et al. 2015; Rosenbaum et al. 2012; Brunel et al. 2001; Buonomano and Maass 2009; Izhikevich et al. 2003; Lisman 1997; Sherfey et al. 2018).

**Fig. 1:**
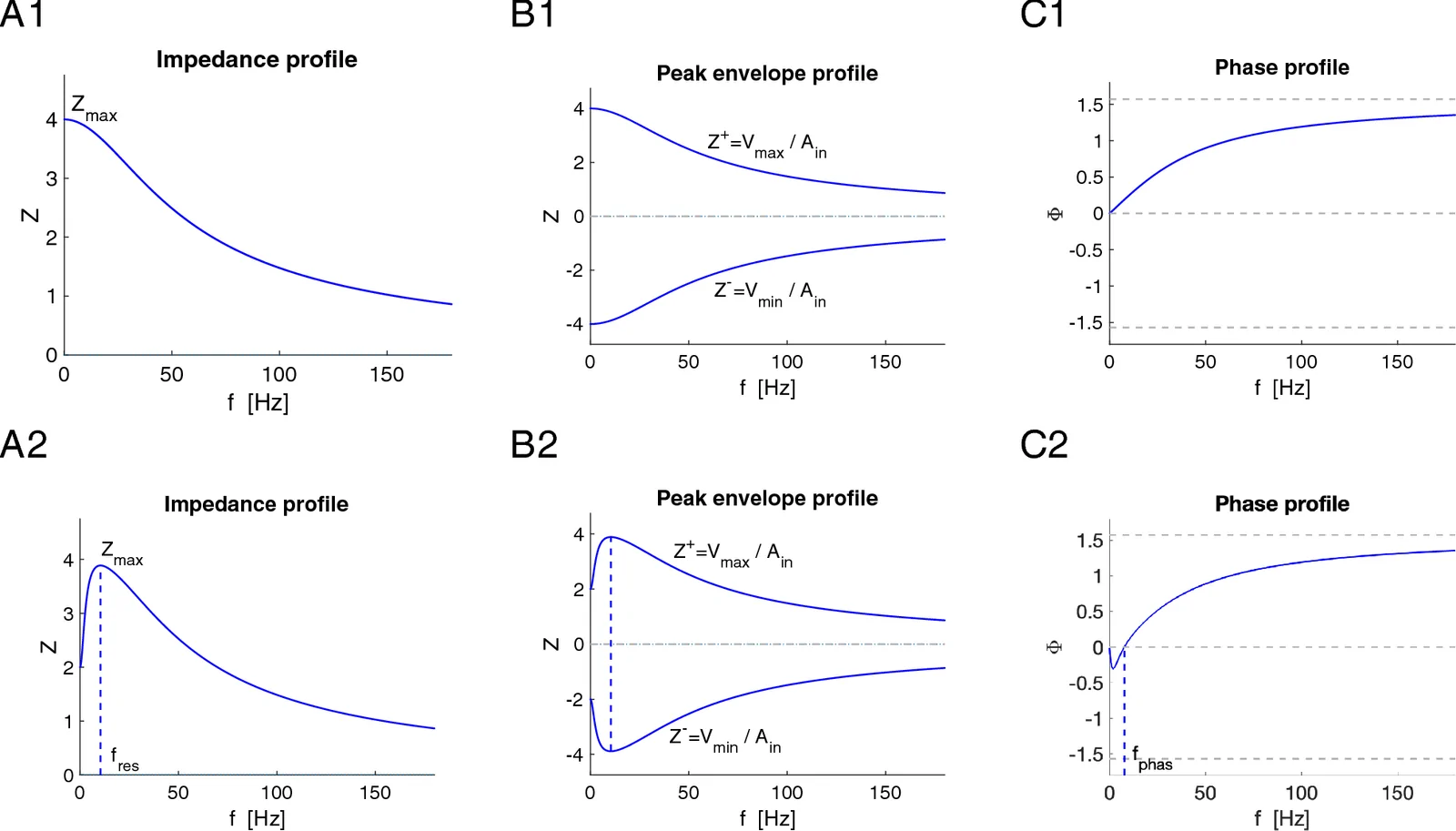
Schematic diagrams of the impedance (A), peak envelope (B) and phase (C) profiles for a passive cell (low-pass filter, LPF, and lagging response; top) and a cell exhibiting resonance (band-pass filter; BPF) and phasonance (leading-lagging response) (bottom). **A.** **A1.** $Z_{max} = Z(0)$. **A2.** Resonance refers to the ability of a cell to exhibit peak in Z(f) at a non-zero (resonant) frequency $f_{res}$. **B1 - B2** $V_{max}$/$V_{min}$ are the steady state peak /trough (maximum/minimum) voltage response values to the oscillatory inputs. $Z^+$ and $Z^-$ are the upper and lower *Z*-envelope profiles, respectively. **C1.** Phasonance refers to the ability of a cell to exhibit a zero phase (phase-shift, Φ=0) response at a non-zero (phasonant) frequency $f_{phas}$. The voltage response lags for $f > f_{phas}$ and leads for $f < f_{phas}$

In this paper we investigate the mechanisms of interaction of cellular neuronal filters in electrically coupled nodes. We use biophysically plausible (conductance-based) mathematical modeling, numerical simulations, analytical calculations and dynamical systems tools. We focus on pairs of gap junction connected cells and two-compartment models of spatially extended neurons. While biologically, these are different structures, dynamically, they can be described by models that belong to the same family of electrically coupled nodes, each of them expressing neuronal amplitude and phase filters. Therefore, their response to frequency-dependent inputs can be investigated within the same mathematical framework.

Two of the most common types of neuronal filters are low-pass (LPFs; e.g., Figs. 1-A1, -B1) and band-pass (BPFs; e.g., Figs. 1-A2, -B2) filters. BPFs are closely linked to the notion of neuronal resonance (Hutcheon and Yarom 2000; Stark et al. 2013, 2022), defined as the ability of a neuronal system to exhibit a maximal response (e.g., subthreshold membrane potential) to periodic inputs at a preferred (resonant), non-zero frequency (or frequency band). In contrast, LPFs describe a preference for inputs in the lowest frequency band. The associated frequency-dependent phase (phase-shift) profiles can be entirely positive (e.g., Figs. 1-C1), indicating a lagging response for all input frequencies, or they can transition from negative to positive as the input frequency increases (e.g., Figs. 1-C2), indicating a mixed leading-lagging response and the occurrence of phasonance (Richardson et al. 2003; Rotstein and Nadim 2014; Rotstein 2014), defined as the ability of a neuronal system to exhibit a zero-phase (phase-shift) response to periodic inputs at a nonzero (phasonant) frequency.

**Fig. 2:**
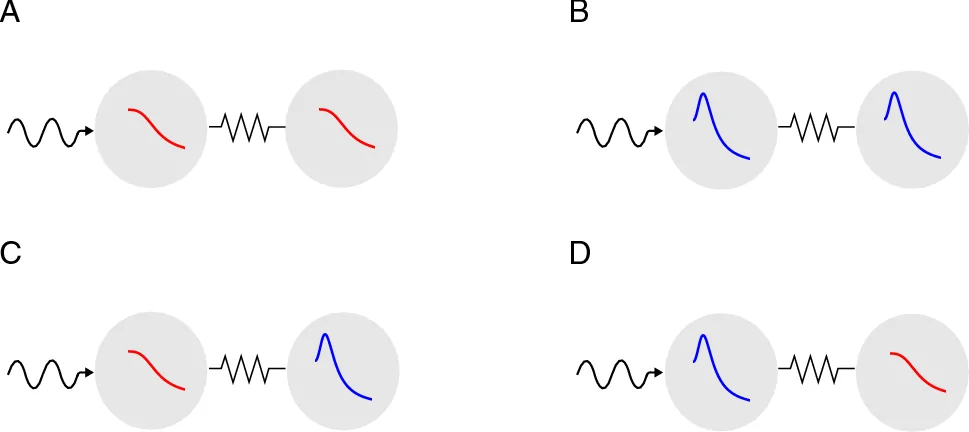
Schematic diagrams of electrically coupled cells receiving oscillatory inputs. The individual cells (disconnected) can have one-dimensional (1D, red) or two-dimensional (2D, blue) dynamics and exhibit either a low-pass filter (LPF; e.g., passive cells, red, see Fig. 1-A1) or a band-pass filter (BPF; e.g., resonators, blue, see Fig. 1-A2). The oscillatory inputs arrives to only one of the cells and are propagated to the second cell. We refer to the cell that receives the input as the pre-J cell and to the other cell as the post-J cell. In our models we use the indices k=1 (pre-J) and k=2 (post-J) cell. The same diagrams represent two-compartment models receiving oscillatory input to only one compartment. The filtering properties of the two connected cells may qualitatively differ from these of the individual (disconnected cells)

Electrical synapses mediated by gap junctions are ubiquitous in the nervous system and coexist with chemical (excitatory and inhibitory) synapses (Dermietzel and Spray 1993, Shimizu and Stopfer 2013, García-Perez et al. 2004, Pereda et al. 1828, Bennett 1977, Connor and Long 2004, Gibson et al. 2005, Landisman et al. 2001, Nielsen et al. 2012, Jing 2017, Curti et al. 2012, Alcamí and Pereda 2019, Bennett and Zukin 2004, Rela and Szczupak 2004). A widely used metric to characterize the strength of electrical synapses is the coupling coefficient (CC) (Shimizu and Stopfer 2013, García-Perez et al. 2004, Hoge et al. 2011, Pereda et al. 1828, Gibson et al. 2005, Bennett 1977, Bennett 1966, Weizel and Schuster 2019, Carnevale and Johnston 1982, Watanabe and Grundfest 1961, Galarreta and Hestrin 1999, Galarreta and Hestrin 2001, Pernelle et al. 2018, Curti and O’brien 2016, Curti et al. 2022, Hjort et al. 2009, Welzel and Schuster 2019), defined by $K = \Delta V_2 / \Delta V_1$ and measured by injecting a step current into one cell and computing the ratio of the steady state voltage deflections of the indirectly ($\Delta V_2$; post-J) and directly ($\Delta V_1$; pre-J) stimulated cells. The larger K, the stronger the degree to which the pre-J and post-J cells are electrically coupled. Following others, we used the terms pre-J and post-J cells in this context (Fig. 2) although electrical transmission mediated by gap junctions is reciprocal (Galarreta and Hestrin 1999).

The magnitude of the CC is determined by the electrical connectivity and the cellular properties and can be altered by neuromodulators and additional synaptic inputs that affect the components of the CC (Sira and Bennett 1972; Curti and O’brien 2016). To the linear level of description, K varies between 0 to 1, increases monotonically with the gap junction conductance ($g_c$) and decreases monotonically with the post-J membrane ($g_{L,2}$) and ionic ($g_2$) conductances (e.g., in passive cells supplemented with a positive or negative ionic current feedback terms). These dependences occur in a balanced, homeostatic manner on the combined parameters $g_{L,2} / g_c$ and $g_2 / g_c$. This characterization determines the efficiency of electrical transmission for heterogeneous network and establishes a path in the flow of information in gap junction connected networks. Electrical transmission is more efficient from the higher to the lower conductances as compared to the reverse direction.

The analysis of more realistic scenarios involving time-dependent inputs and the effects of the intrinsic cellular time constants requires extending the notion of CC to include the response of gap junction networks to oscillatory inputs (Curti and O’brien 2016, Galarreta and Hestrin 1999, Curti et al. 2012, García-Perez et al. 2004, Gibson et al. 2005). This leads to the frequency-dependent (amplitude) coupling coefficient profile $K(f) = A_2(f)/A_1(f)$, where $A_{1,2}(f)$ are the voltage amplitude responses of the pre-J and post-J cells, respectively, as a function of the input frequency f, and the associated phase-difference coefficient profile $\Delta \Phi (f) = \Phi _2(f) - \Phi _1(f)$ where $\Phi _{1,2}(f)$ are the corresponding voltage phase responses.

While the K(f) and $\Delta \Phi (f)$ profiles have been discussed in the literature (Curti and O’brien 2016, Galarreta and Hestrin 1999, Curti et al. 2012, García-Perez et al. 2004, Gibson et al. 2005, Curti et al. 2022, Dugué et al. 2009, Curti and Pereda 2004, Davoine et al. 2020, Li et al. 2023), it is largely unknown how they are shaped by the inherently nonlinear ionic currents and the multiplicity of time scales present in neurons (but see Curti and O’brien (2016)), particularly in the presence of resonance (BPFs) and phasonance (mixed negative-positive phase responses) in the pre-J and post-J cells. Furthermore, existing theoretical studies have largely linear systems, primarily passive cell models or equivalent electric circuits (but see Curti and O’brien 2016, Curti et al. 2012, Dugué et al. 2009, Curti and Pereda 2004, Davoine et al. 2020, Li et al. 2023) for which the K(f) and $\Delta \Phi (f)$ profiles depend on the biophysical properties of the post-J cell and the gap junction conductance, and are independent of the biophysical properties of the pre-J cell and the input amplitude (Curti and O’brien 2016) (revisited in Section 3.1.1). It remains unclear how the latter two affect the K(f) and $\Delta \Phi (f)$ profiles when the neurons operate away from their linear regime.

**Table 1:** Glossary. The precise definitions are provided in Section 2

Context	Symbol	Meaning
Neuronal	LPF	Low-pass filter: see Fig. 1-A1
filters	BPF	Band-pass filter: see Fig. 1-A2
Electrically	pre-J	The node that directly receives the input (Fig. 2, left node)
coupled	post-J	The node that indirectly receives the input (Fig. 2, right node)
network		Nodes: Cells or compartments
Index	pre-J	k=1
	post-J	k=2
Frequency	f	Input frequency (in Hz)
	$f_{res}$	Resonant frequency
	$f_{phas}$	Phasonant frequency
	Ω	$= 2\, \pi \, f / T_{scale}$ ($T_{scale} = 1000$ ms)
Single cell	$\mathbf{Z(f)}$	Complex impedance
response	Z(f), A(f)	Impedance amplitude
profiles	Φ(f)	Impedance phase
	$V_{max}(f)$	Peak envelope
	$V_{min}(f)$	Trough envelope
	${\hat{\textbf{Z}}}(f)$	Extended (complex) impedance
	$\hat{Z}(f)$	Extended impedance amplitude
	$\hat{\Phi }(f)$	Extended impedance phase
Individual cell	$A_{k}(f)$	Amplitude response
(network)	$\Phi _k(f)$	Phase response
response	$\textbf{A}_k(f)$	Complex amplitude response
profiles:	$\textbf{Z}_k(f)$	Complex network impedance
cell k	${\hat{\textbf{Z}}}_k^0(f)$	Extended (complex) impedance
	$\hat{Z}_k(f)$	Extended impedance amplitude
	$\hat{\Phi }_k(f)$	Extended impedance phase
Coupling and	K(f)	Coupling coefficient
phase-difference	$\Delta \Phi (f)$	Phase-difference coefficient
coefficients	$f_{res,K}$	Resonant (peak) frequency for K
	$f_{phas,\Delta \Phi }$	Phasonance (zero-phase) frequency for $\Delta \Phi$

The generation of cellular resonance and phasonance in individual cells requires the interplay of negative and positive feedback effects mediated by the gating variables associated to the intrinsic slow resonant currents (e.g., hyperpolarization-activated mixed-cation $$ I_h$$, M-type slow potassium $$I_{M}$$ and T-type calcium $$I_{CaT} $$ inactivation) and fast amplifying currents (e.g., persistent sodium $$ I_{Nap} $$ and $$ I_{Ca,T} $$ activation), which oppose and favor changes in voltage, respectively (Hutcheon and Yarom 2000; Richardson et al. 2003; Rotstein and Nadim 2014). The presence of amplifying and resonant ionic currents has been proposed to regulate the frequency-dependent coupling by increasing the K(f) profile (Curti et al. 2012, Dugué et al. 2009, Curti and Pereda 2004) or by causing K(f) profile to be a BPF (Curti et al. 2012; Davoine et al. 2020), indicating the existence of an intermediate frequency band at which the transmission of information is more efficient than for other frequencies. However, a systematic study of how presence cellular resonance and phasonance in the participating cells shape the K(f) and $$ \Delta \Phi (f) $$ profiles is lacking.

**Fig. 3:**
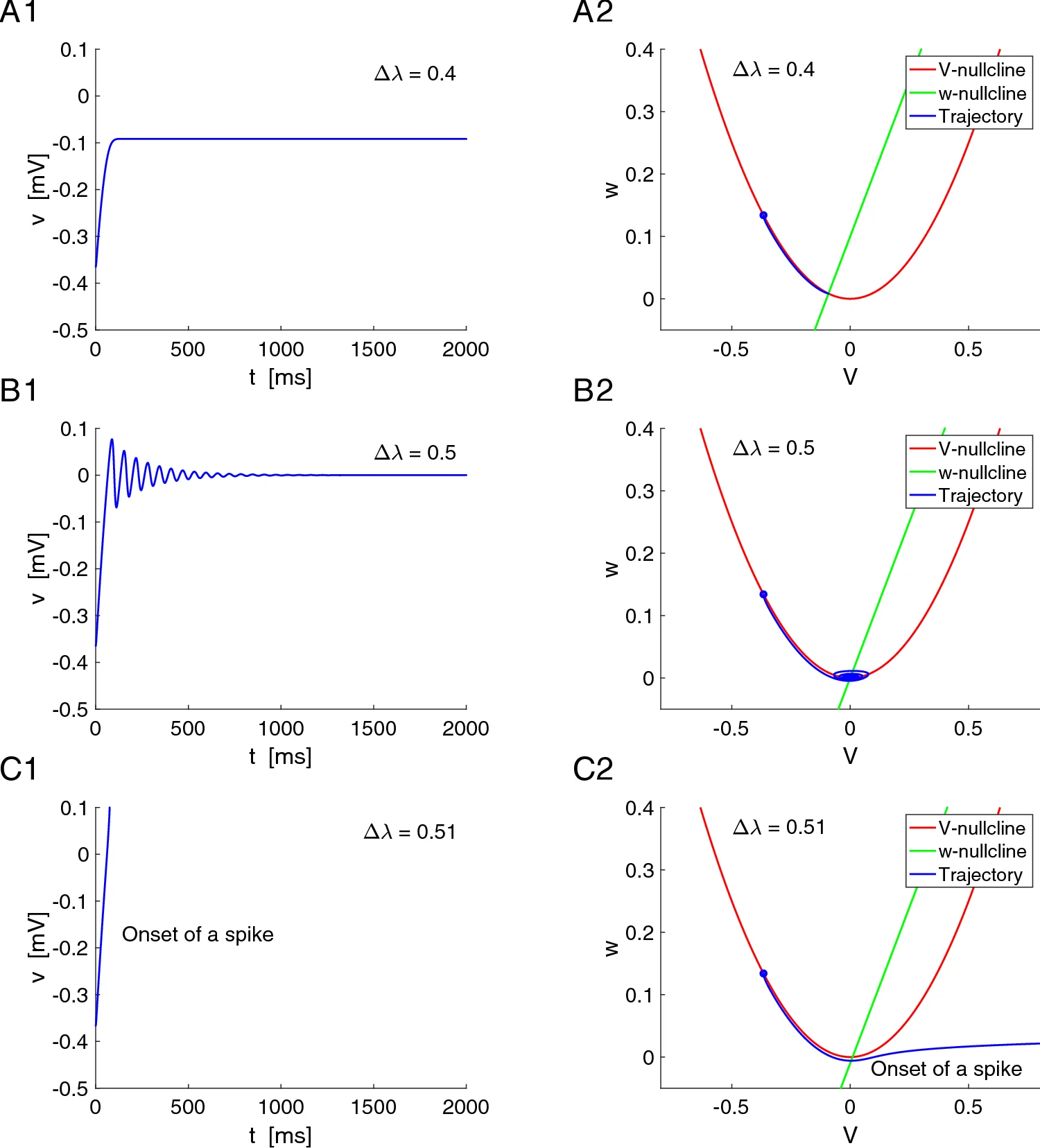
Response of a single cell quadratic model to constant inputs: Voltage traces and phase-plane diagrams. We used the model (3)-(4) for a single cell ($g_c = 0$) with a=1, α=1 and ϵ=0.01. The initial conditions (blue dots) were determined as the steady-state response to a baseline value of λ: $\lambda _{base} = -0.5$ and the values of λ were determined as $\lambda = \lambda _{base} + \Delta \lambda$. **Left Column.** Voltage traces. **Right column.** Phase-plane diagrams. The V-nullclines (red) are the curves satisfying $w = a\, v^2$ and the w-nullclines (green) are the lines satisfying $w = \alpha \, v - \lambda$. The fixed-point is determined by the intersection between the two nullclines. The trajectories (blue) are initially located at the blue dot and evolve towards the fixed-point. **A.** $\Delta \lambda = 0.4$ (λ=-0.1). The model exhibits an almost monotonic increase to the steady state value of v (there is an imperceptible overshoot at low values of t). **B.** $\Delta \lambda = 0.5$ (λ=0). The model exhibits subthreshold oscillations around the steady-state value of v. **C.** $\Delta \lambda = 0.51$ (λ=0.1). The model produces the onset of spikes when the trajectory increases unboundedly and escapes the subthreshold voltage regime. The model ceases to be a good approximation to any biophysical model. A voltage reset mechanism is needed to bring the trajectory back to the subthreshold regime

Multicompartment models of spatially extended neurons have been used to investigate the neuronal resonant properties distributed along the somato-dendritic axis and how the neuronal intrinsic ionic currents affect the response to, and propagation of spatially localized inputs (Hu et al. 2002, 2009; Narayanan and Johnston 2008; Laudansky et al. 2014; Ostojic et al. 2015; Zhuchkova et al. 2014, 2013; Kasevich and LaBerge 2011; Oz et al. 2022; Ladenbauer et al. 2012). In an intriguing set of results (Hu et al. 2002, 2009), it was experimentally demonstrated that two different types of subthreshold theta resonances (Hu et al. 2002, 2009; Pike et al. 2000; Leung and Yu 1998; Zemankovics et al. 2010) coexist along the somato-dendritic axis n hippocampal CA1 pyramidal cells (*in vitro*): (i) a perisomatic resonance generated by a slow-potassium M-type current ($I_M$) and amplified by a persistent sodium current ($I_{Nap}$), and (ii) a dendritic resonance generated by a hyperpolarization-activated mixed-cation (sodium and potassium) current or h-current ($I_h$). The mechanisms that control the interaction between these two types of resonances are not well understood.

**Fig. 4:**
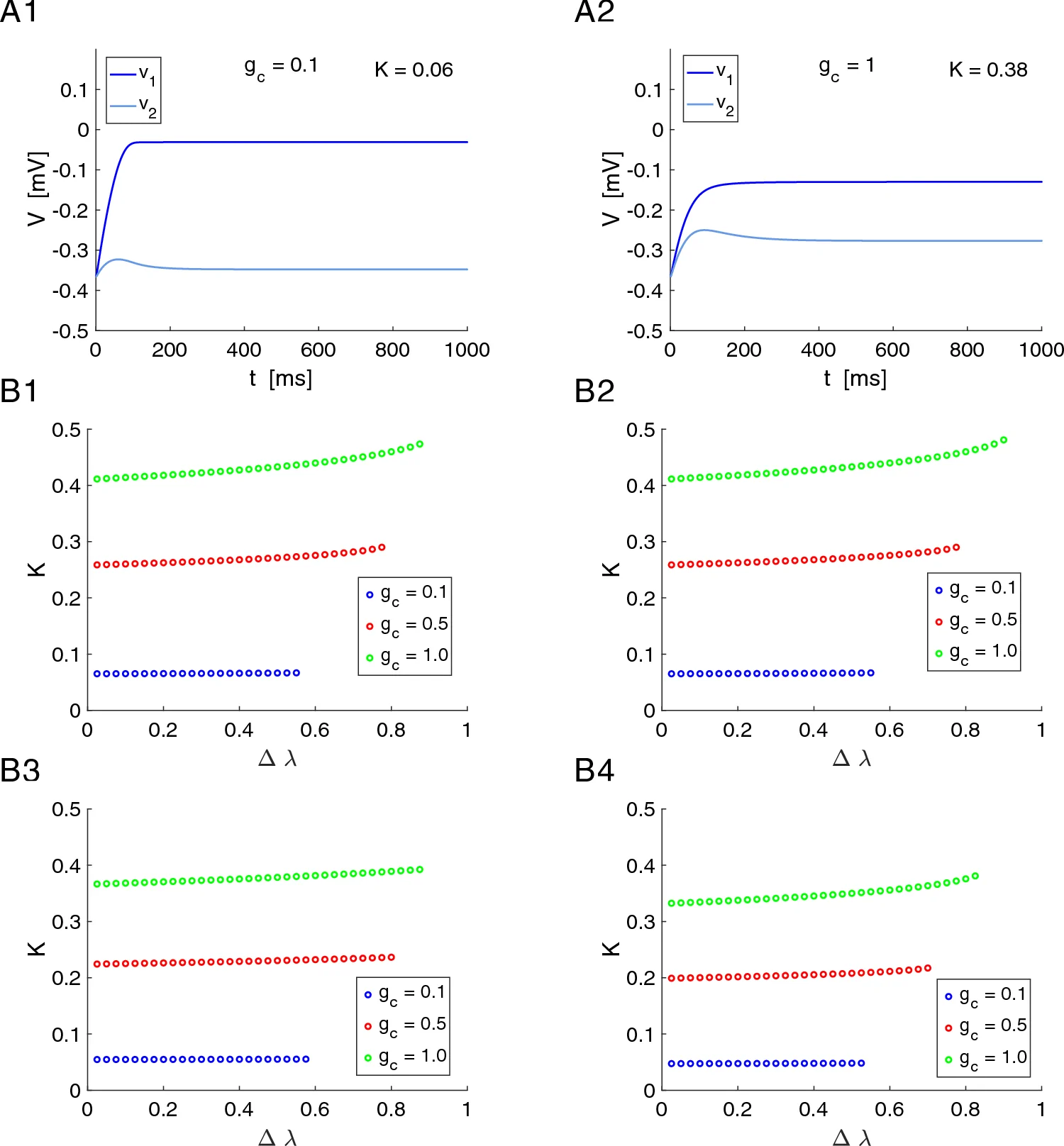
Response of a single cell quadratic model to constant inputs: Coupling coefficient. We used the model (3)-(4) with a=1, α=1 and ϵ=0.01. The initial conditions were determined as the steady-state response to a baseline value of λ: $\lambda _{base} = -0.5$ and the values of λ were determined as $\lambda = \lambda _{base} + \Delta \lambda$. **A.** Voltage traces for representative examples and λ=0.5. **A1.** $g_c = 0.1$. **A2.** $g_c = 1$. **B.** Dependence of K on $\lambda = \lambda _{base} + \Delta \lambda$ for representative values of $g_c$, ϵ, α and a. **B1.** a=1, α=0.25, ϵ=0.01. **B2.** a=1, α=0.25, ϵ=0.1. **B3.** a=1, α=1, ϵ=0.01. **B4.** a=2, α=0.25, ϵ=0.01

We address these issues systematically in this paper. We consider a network of two electrically connected neurons mediated by gap junctions receiving external inputs (Fig. 2). This is the minimal network architecture that allows for a systematic study of the biophysical and dynamic mechanisms that shape the K(f) and $\Delta \Phi (f)$ profiles. The participating neurons are either passive cells (LPFs; 1D; Fig. 1-A1) or resonators (BPFs; 2D; Fig. 1-A2) and exhibit positive (Fig. 1-C1) or mixed negative-positive (Fig. 1-C2; phasonators) phase responses. We begin our study by using linear models to understand how the participating building blocks (e.g., conductances, negative and positive feedback effects) operating at the single node level control the K(f) and $\Delta \Phi (f)$ profiles.

**Fig. 5:**
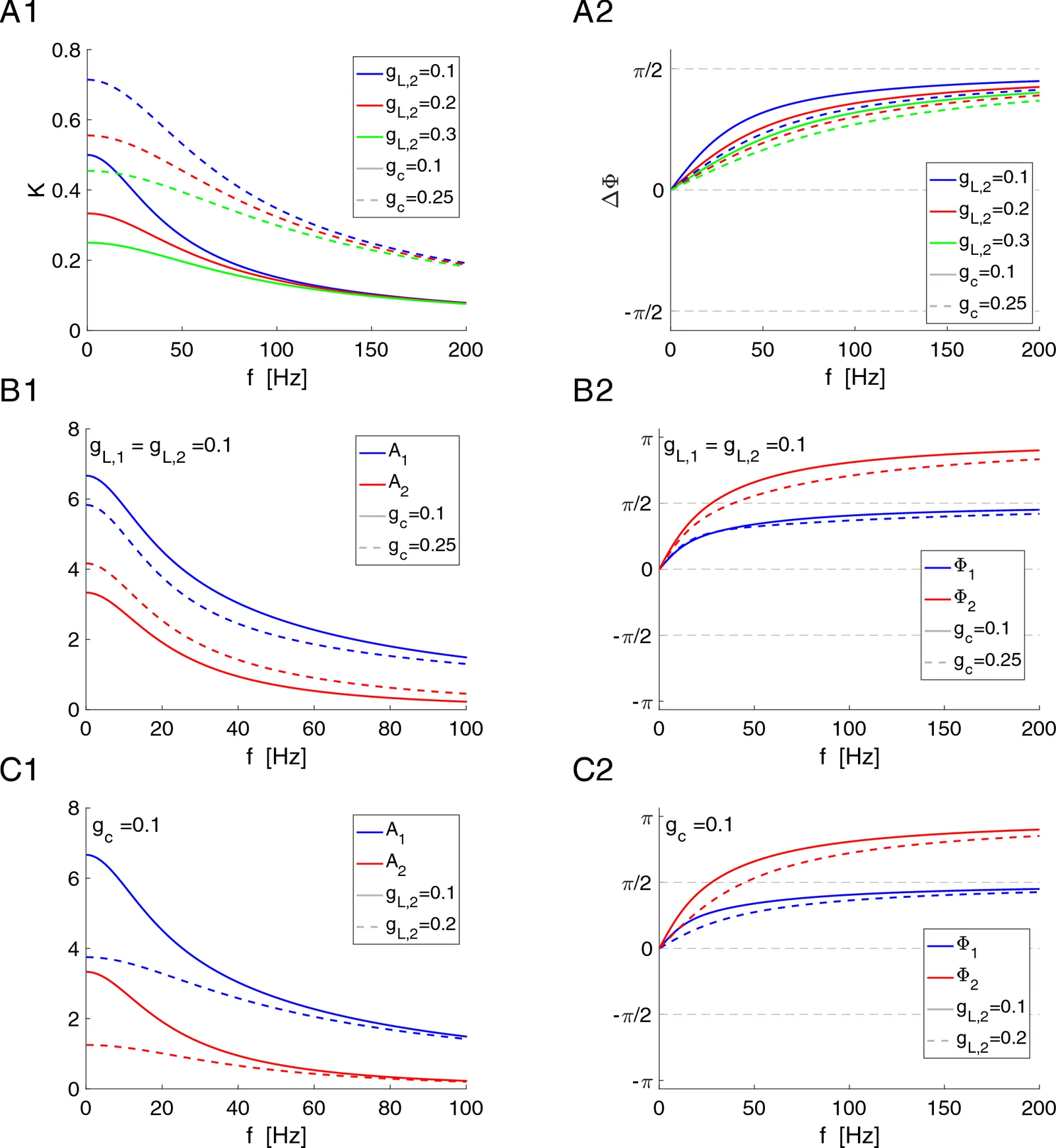
Response of two electrically coupled passive cells to oscillatory inputs for representative parameter values. The passive cell model is given by eqs. (1)-(2) with $g_1 = g_2 = 0$. Cells 1 and 2 are the pre-J and post-J cells, respectively. **A.** Coupling and phase-difference coefficients. **A1.** Coupling coefficient K given by eq. (26). **A2.** Phase-difference coefficient $\Delta \Phi$ given by eq. (27). **B.** Amplitude and phase response profiles for the pre-J (blue) and post-J (red) cells for representative values of $g_{L,1}$, $g_{L,2}$ and $g_c$. The blue and red curves correspond to the solid- and dashed-blue curves in panels A, respectively. These quantities were computed using eqs. (7)-(8) with $g_1 = g_2 = 0$. **B1.** Amplitude response profiles. **B2.** Phase response profiles. **C.** Amplitude and phase response profiles for the pre-J (blue) and post-J (red) cells for representative values of $g_{L,1}$, $g_{L,2}$ and $g_c$. The blue curves and red curves correspond to the solid- and dashed-blue curves in panels A, respectively. These quantities were computed using eqs. (7)-(8) with $g_1 = g_2 = 0$. **C1.** Amplitude responses. **C2.** Phase responses

We then turn our attention to linearized models of neurons with biophysically realistic ionic currents and investigate (to the linear level of approximation) how the presence of cellular ionic currents and cellular resonance and phasonance contribute to shaping the K(f) and $$ \Delta \Phi (f) $$ profiles. We use models having $$ I_{Nap} $$, $$ I_h $$ and $$ I_{Ks} $$ for parameter values within the physiological range motivated by the experimental results mentioned above and previous studies (Rotstein and Nadim 2014; Rotstein 2017a, b; Acker et al. 2003; Richardson et al. 2003). The linearized parameters (i.e., the parameters in the linearized models) are recalculated for each set of biophysical parameter values of the (biophysical) nonlinear model whose dynamics they capture. Therefore, the linearized models capture certain nonlinear dependencies with the model parameters (Rotstein and Nadim 2014). The $$ I_{Nap} $$ + $$ I_h $$ and $$ I_{Nap} $$ + $$ I_{Ks}$$ models we use are representative models of the interaction between resonant ($$I_h $$ and $$ I_{Ks} $$) and amplifying ($$I_{Nap}$$) currents where $$ I_h $$ and $$ I_{Ks} $$ have opposite depolarization voltage dependencies and reversal potentials relative to the resting potential. This allow us to test scenarios that are biophysically different (different combinations of ionic currents), but dynamically similar (interaction of negative and positive feedback effects).

**Fig. 6:**
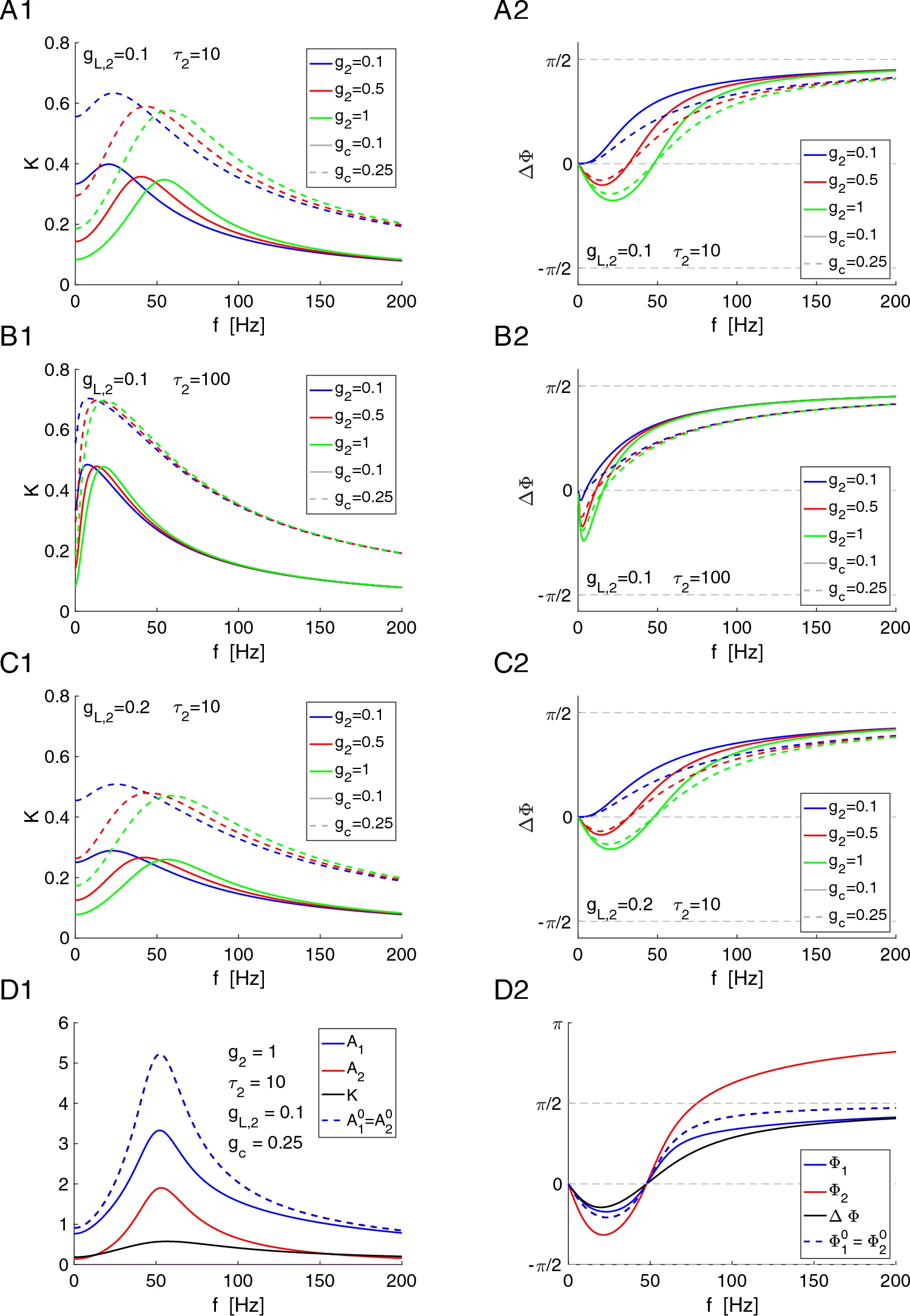
Response of two electrically coupled resonators to oscillatory inputs for representative parameter values. The linear model is given by eqs. (1)-(2). The pre-J and post-J cells (1 and 2, respectively) are identical. **A1, B1, C1.** Coupling coefficients K(f) given by eq. (28). **A2, B2, C2.** Phase-difference coefficient $$ \Delta \Phi (f) $$ given by eq. (29). **D.** Network amplitude ($$A_1 $$ and $$ A_2 $$, panel D1) and phase ($$\Phi _1$$ and $$ \Phi _2$$, panel D2) responses computed from the complex responses $$ \mathbf{A_1} $$ and $$ \mathbf{A_2}$$ given by eqs. (7)-(8) for the parameter values used for the dashed-green curves in panels A. Superimposed to these profiles are the amplitudes of the individual cells ($$A_1^0 $$ and $$ A_2^0 $$, dashed-blue in panel D1) and the coupling coefficient K (panel D1), and the phase of the individual cells ($$\Phi _1^0$$ and $$ \Phi _2^0$$, dashed-blue in panel D2) and the phase-difference coefficient $$ \Delta \Phi $$ (panel D2)

We then extend our investigation to include the model nonlinearities to understand how the K(f) and $$ \Delta \Phi (f) $$ profiles are shaped by the interaction between the pre-J and post-J cells, and not just the post-J cells as for linear models. To this end, we used the quadratized $$ I_{Nap} $$ + $$ I_h $$ and $$ I_{Nap} $$ + $$ I_{Ks}$$ models (Rotstein 2015; Turnquist and Rotstein 2018; Chialva et al. 2023). These are two-dimensional models with parabolic nonlinearities in the voltage equation and linear dynamics for the recovery variable. Similarly to the linearized models, the parameters in the quadratized model are linked to the biophysical parameter values of the (biophysical) nonlinear model whose dynamics they capture, and can be recalculated for each set of biophysical parameter values.

**Fig. 7:**
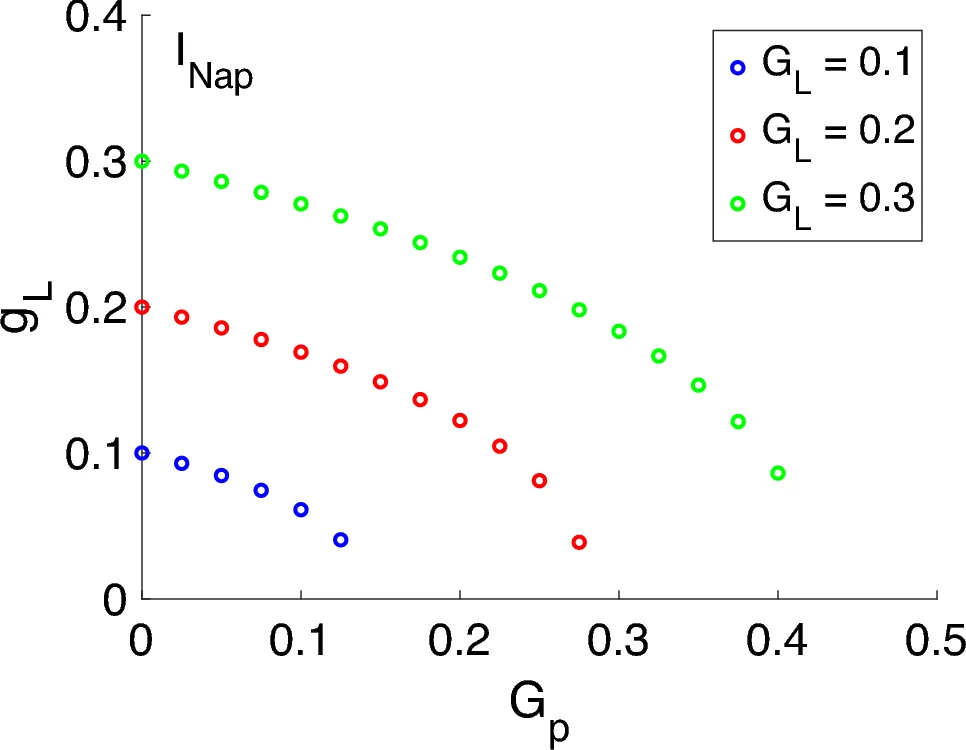
$$ \mathbf{I_{Nap}} $$ model: Dependence of the linearized conductance $$ \mathbf{g_L} $$ on the biophysical conductances $$ \mathbf{G_p} $$ and $$ \mathbf{G_L} $$. We used eq. (30) with the parameter values presented in Section A.2 and $$I_{app} = 0 $$

As a first step, we used symmetric connectivity. However, gap junctions can be rectifying (Marder 2009; Fortier 2010) (see references therein) and compartmental models (e.g., dendritic) are typically asymmetrically connected as the result of the different geometries of the participating compartments (Dayan and Abbott 2001; Ermentrout and Terman 2010). We investigate the effects of asymmetric connectivity to understand how the compartmental geometry interacts with the neuronal biophysical properties to shape the K(f) and $\Delta \Phi (f)$ profiles.

The formalism and tools we develop and use in this paper can be extended to larger networks with an arbitrary number of nodes, to spatially extended multicompartment neuronal models and to models having ionic currents with regenerative and restorative processes such as the ($I_{Ca,T}$) activation and inactivation.

Our results make experimentally testable predictions and have implications for the understanding of spike transmission, synchronic firing and coincidence detection in electrically coupled networks in the presence of oscillatory inputs.

Table 1 summarizes the notation used throughout the paper. The precise definitions are provided in Methods.

## Methods

We consider a network of two electrically connected neurons mediated by gap junctions receiving external inputs (Fig. 2). With minimal modifications, the formalism we use describes two-compartment neurons receiving external inputs. The dynamics of the individual neurons are described by reduced, linearized (Richardson et al. 2003; Rotstein and Nadim 2014) or quadratized (Turnquist and Rotstein 2018; Rotstein 2015; Chialva et al. 2023) biophysical (conductance-based) models of Hodgkin-Huxley (HH) type (Hodgkin and Huxley 1952; Ermentrout and Terman 2010). The models include non-spiking ionic currents such as the persistent sodium current ($$I_{Nap}$$), the hyperpolarization-activated mixed-cation ($$ I_h$$) current, the M-type slow-potassium current ($$I_{Ks}$$). $$ I_{Nap} $$ is regenerative (amplifying, favors changes in voltage, provides positive feedback effects; Fig. 20-C, red) and has fast dynamics, and it is assumed to be slaved to voltage, while $$ I_h $$ and $$ I_{Ks} $$ are restorative (resonant, oppose changes in voltage, provide negative feedback effects; Fig. 20-C, blue and green) and have slower dynamics (Izhikevich 2006; Rotstein et al. 2006; Rotstein 2015, 2017a, b).

We refer the reader to the Appendix A for a description of the biophysical $I_{Nap}$, $I_{Nap}$+$I_h$ and $I_{Nap}$ + $I_{Ks}$ models of HH type in the subthreshold voltage regime (Sections A.1 and A.2) and for the details on the linearization (Section A.3) and quadratization (Section A.4) processes. A biophysical interpretation of the linear (linearized) and quadratic (quadratized) models in terms of the biophysical models of HH type can be achieved by using the formulas linking the biophysical parameters with the parameters of the reduced linearized (Section A.3) and quadratized models (Section A.4).

**Fig. 8:**
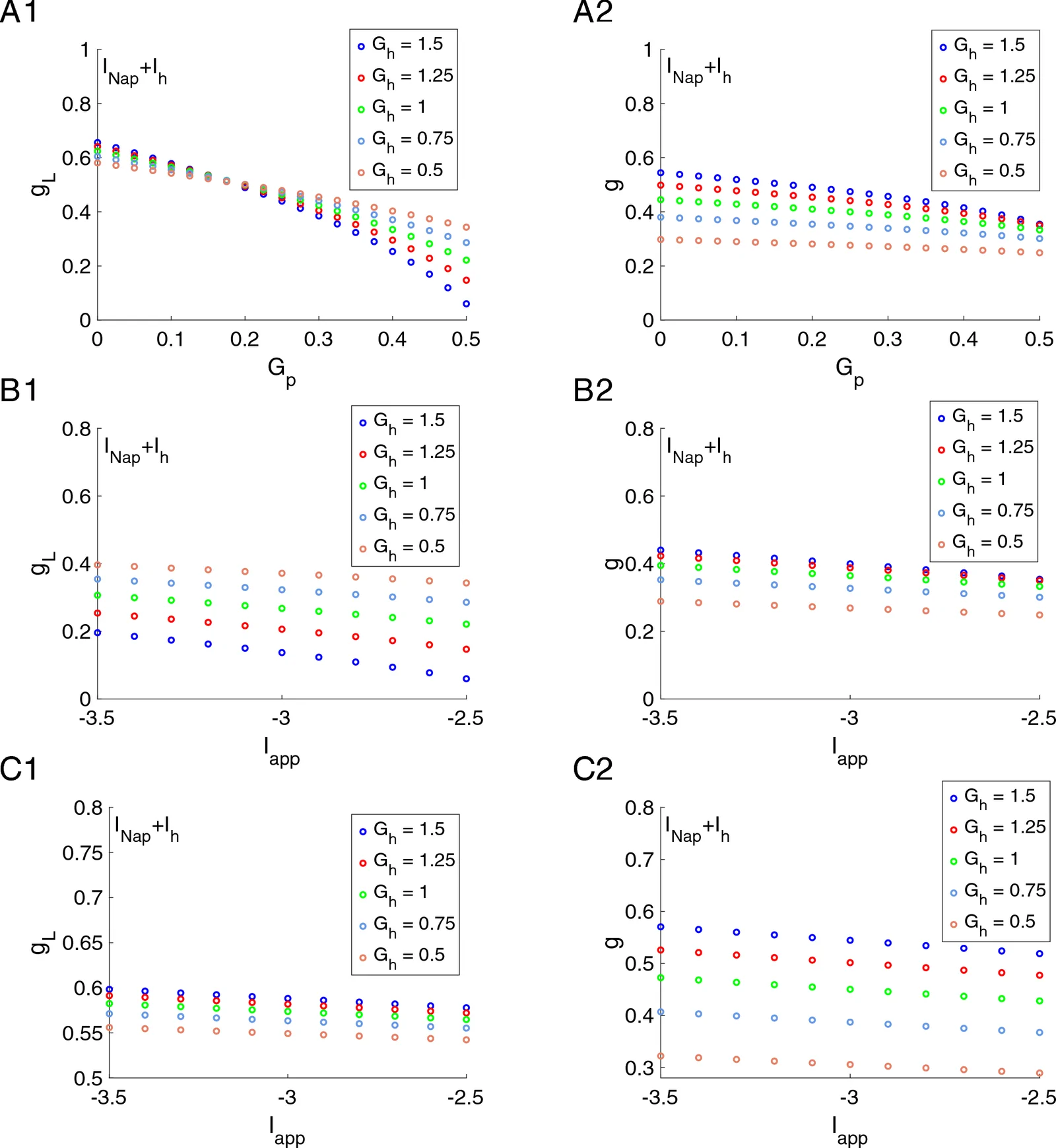
Dependence of the linearized conductances ($$\mathbf{g_L}$$ and $$\textbf{g}$$) on the biophysical maximal conductances ($$\mathbf{G_p}$$ and $$ \mathbf{G_h} $$) and $$ \mathbf{I_{app}}$$ for the $$\mathbf{I_{Nap}} $$+$$\mathbf{I_h}$$ model for representative parameter values. We used the biophysical model (A-1)-(A-2) with the parameter values presented in the Appendix A.2 and the linear model (1)-(2). The linearization process is described in the Appendix A.3**A.** $$ g_L $$ and g as a function of $$ G_p $$ and $$ G_h $$ for $$ G_L = 0.5 $$ and $$ I_{app} = -2.5$$. **B.** $$ g_L $$ and g as a function of $$ I_{app} $$ for $$ G_L = 0.5 $$ and $$ G_p = 0.5 $$. **C.** $$ g_L $$ and g as a function of $$ I_{app} $$ for $$ G_L = 0.5 $$ and $$ G_p = 0.1 $$

**Fig. 9:**
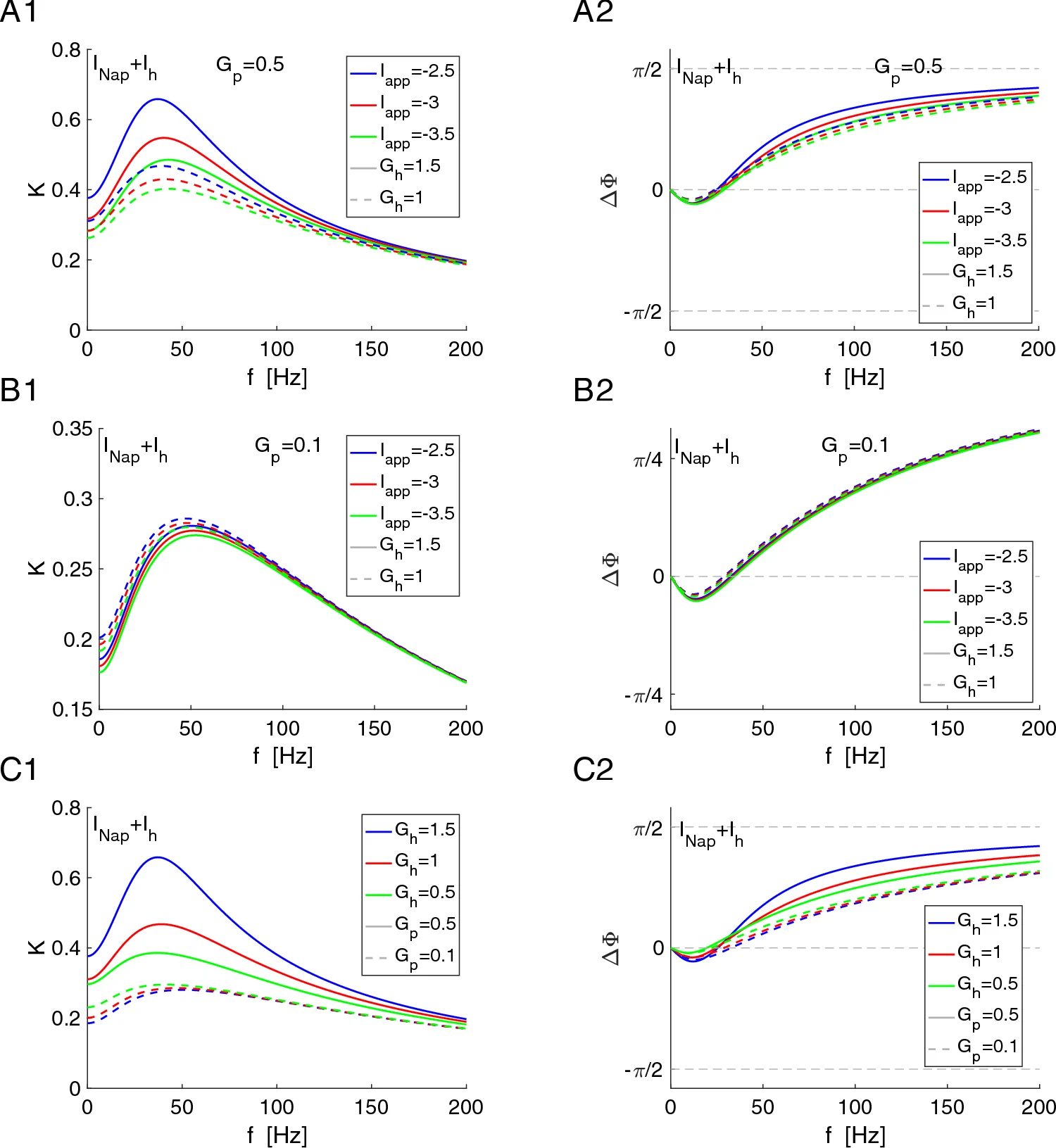
$$ \textbf{K} $$- and $$ \mathbf{\Delta \Phi } $$- profiles for the $$ \mathbf{I_{Nap}} $$ + $$\mathbf{I_h}$$ model for representative parameter values. We used eqs. (28)- (29) for the biophysical $$ I_{Nap} $$ + $$ I_h $$ model (A-1)-(A-2) with the parameter values presented in the Appendix A.2. The linearization process leading to the linearized model (1)-(2) is described in the Appendix A.3. **Left column.** K-profiles. **Right column.** $$ \Delta \Phi $$-profiles. **A.** $$ G_p = 0.5 $$, $$ G_L = 0.5 $$. **B.** $$ G_p = 0.1 $$, $$ G_L = 0.5 $$. **C.** $$ I_{app} = -2.5 $$, $$ G_L = 0.5$$

We use the following units for all the models in this paper: mV for voltage, ms for time, μF/cm^2^  for capacitance, μA/cm^2^  for current, mS/cm^2^  for the maximal conductances and Hz for frequency.

### Networks of electrically coupled linearized cells

The linearized network model is described by (see Appendix A.3)

1 $$\begin{aligned} C_k\, \frac{dv_k}{dt} = -g_{L,k}\, v_k - g_{k}\, w_{k} + g_c\, (v_j - v_k) + I_{in,k}(t), \end{aligned}$$

2 $$\begin{aligned} \tau _{k}\, \frac{dw_{k}}{dt} = v_k - w_{k}, \end{aligned}$$

**Fig. 10:**
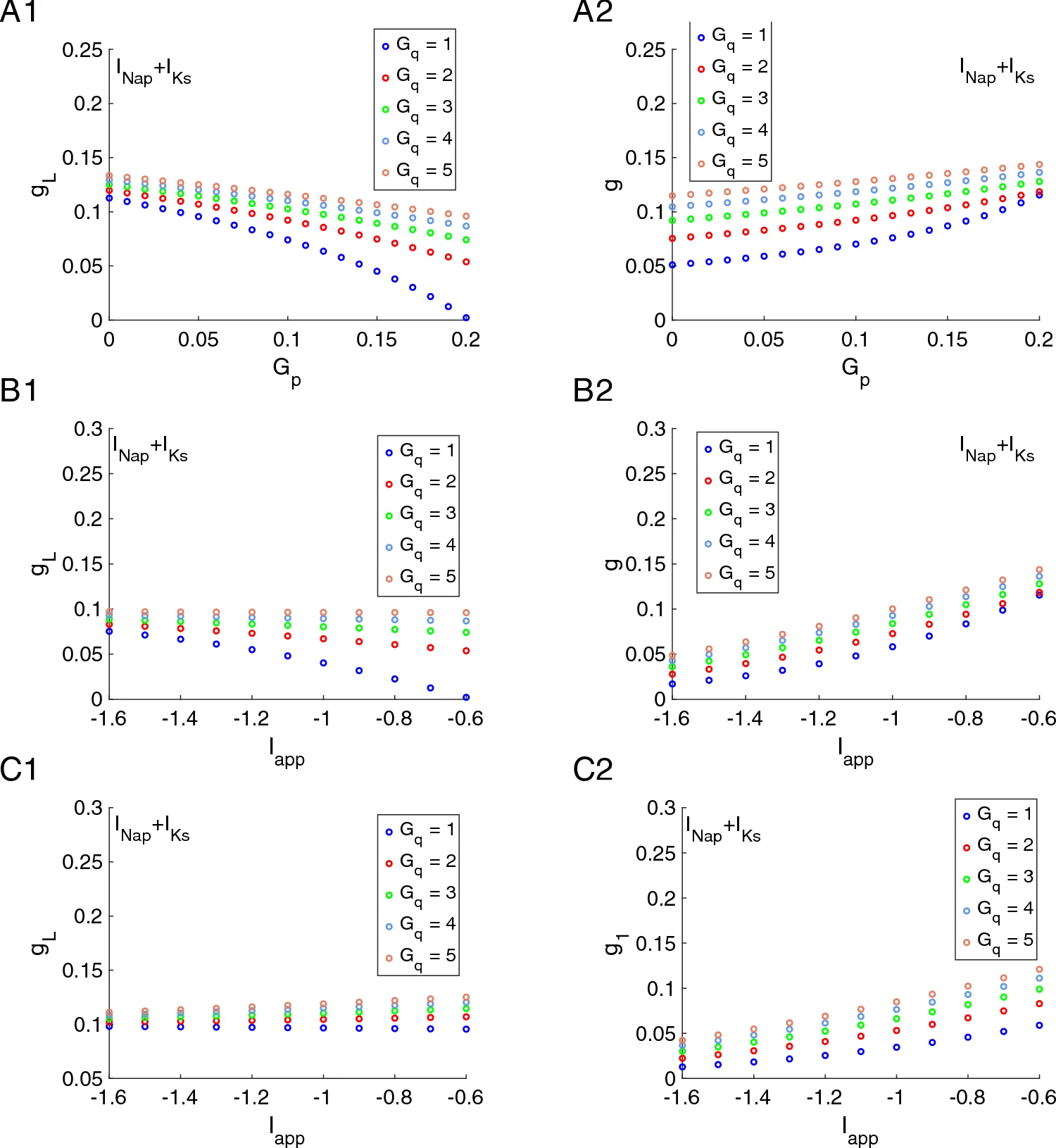
Dependence of the linearized conductances ($$\mathbf{g_L}$$ and $$\textbf{g}$$) on the biophysical maximal conductances ($$\mathbf{G_p}$$ and $$ \mathbf{G_q} $$) and $$ \mathbf{I_{app}}$$ for the $$\mathbf{I_{Nap}} $$+$$\mathbf{I_{Ks}}$$ model for representative parameter values. We used the biophysical model (A-1)-(A-2) with the parameter values presented in the Appendix A.2 and linear model (1)-(2). The linearization process is described in the Appendix A.3**A.** $$ g_L $$ and g as a function of $$ G_p $$ and $$ G_q $$ for $$ G_L = 0.1 $$ and $$ I_{app} = -0.6$$. **B.** $$ g_L $$ and g as a function of $$ I_{app} $$ for $$ G_L = 0.1 $$ and $$ G_p = 0.2 $$. **C.** $$ g_L $$ and g as a function of $$ I_{app} $$ for $$ G_L = 0.1 $$ and $$ G_p = 0.05 $$. AB: Panel C2: the ordinate should be g instead of $$ g_1 $$

**Fig. 11:**
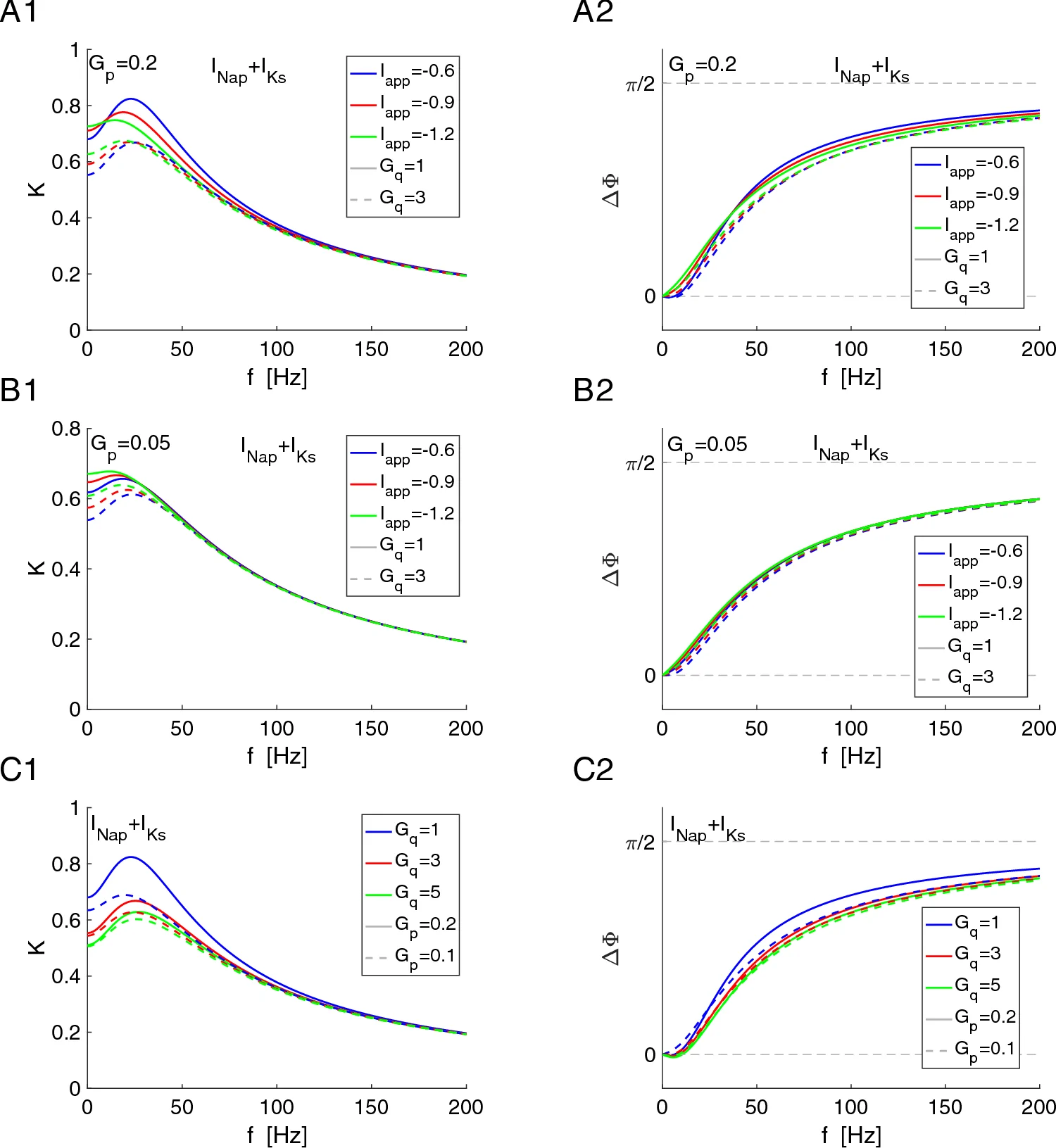
$$ \textbf{K} $$- and $$ \mathbf{\Delta \Phi } $$- profiles for the $$ \mathbf{I_{Nap}} $$ + $$\mathbf{I_{Ks}}$$ model for representative parameter values. We used eqs. (28)- (29) for the biophysical $$ I_{Nap} $$ + $$ I_{Ks} $$ model (A-1)-(A-2) with the parameter values presented in the Appendix A.2. The linearization process leading to the linearized model (1)-(2) is described in the Appendix A.3. **Left column.** K-profiles. **Right column.** $$ \Delta \Phi $$-profiles. **A.** $$ G_p = 0.2 $$, $$ G_L = 0.1 $$. **B.** $$ G_p = 0.05 $$, $$ G_L = 0.1 $$. **C.** $$ I_{app} = -0.6 $$, $$ G_L = 0.1$$

for k,j=1,2. In eqs. (1)-(2), t is time, $$ v_k $$ represent the membrane potential, $$ w_{k} $$ represent the gating variable, $$ C_k $$ are the capacitances, $$ g_{L,k} $$ are the linearized leak maximal conductances, $$g_{k} $$ are the ionic current linearized conductances, $$ \tau _{k} $$ are the linearized time constants for the linearized gating variables $$ w_k$$, $$ g_c $$ is the gap junction coupling coefficient, and $$I_{in,k}(t) $$ are time-dependent input currents. The linearized component of the ionic currents with instantaneously fast dynamics (e.g., $$ I_{Nap} $$) are incorporated in $$ g_{L,k} $$. For two-compartment models with different connectivity coefficients and for rectifying gap junctions, we use the notation $$ g_c = g_{c,kj} $$.

Note that the heterogeneity due to different values of the biophysical parameters in the original conductance-based model is translated into the linearized model both explicitly and implicitly through the equilibria $(\bar{V}_1$ and $\bar{V}_2)$ of the original system. Unless stated otherwise, we use $C_1=C_2 = 1$ for all models in this paper.

### Networks of electrically coupled quadratized cells

The quadratized network model is given by (see Appendix A.4)

3 $$\begin{aligned} \frac{dv_k}{dt} = a v_k^2 - w_k + g_c\, (v_j - v_k) + I_{in,k}(t), \ \ \ \ \ \ \ \ \, \end{aligned}$$

4 $$\begin{aligned} \frac{dw_k}{dt} = \epsilon \, [\, \alpha \, v_k - \lambda - w_k\, ], \end{aligned}$$

for k,j=1,2, k≠j, where a>0 captures the effects of an amplifying current, $g_c$ represents the gap junction coupling coefficient, ϵ is the inverse of the time constant of the resonant gating variable, $\alpha \ge 0$ represents the strength of the negative feedback (resonant) current, and λ is a combination of parameters including, and having the same sign as $I_{app}$ in the original biophysical model. Increasing (decreasing) values of $I_{app}$ cause λ to increase (decrease) and displaces the w-nullcline to the right (left), thus favoring (opposing) the generation of subthreshold oscillations and spikes (Fig. 3). Geometrically, a controles the curvature of the parabolic v-nullcline, α controls the slope of the w-nullcline, ϵ represents the time scale separation between the two variables, and λ controls the displacement of the w-nullcline with respect to the v-nullcline. As for the linear models, for two-compartment models with different connectivity coefficients or rectifying gap junctions, we use the notation $g_c = g_{c,kj}$.

Nonlinearities of parabolic type in the subthreshold voltage regime are generated in the presence of regenerative/amplifying currents (e.g., $$I_{Nap}$$). The resulting voltage nullclines of parabolic type F(ig. 20-A3 and -B3) are shaped by the interplay of these and the other participating ionic currents (e.g., $$ I_h $$, $$ I_{Ks}$$).

**A note about quadratization**

Quadratization of biophysically plausible models of HH type extends the notion of linearization to include the parabolic-like properties of the V-nullcline in the subthreshold regime and therefore capture more realistic aspects of the dynamics of these models, which can be missed by the corresponding linearization (Izhikevich 2006, 2010, 2003; Rotstein 2015; Turnquist and Rotstein 2018; Chialva et al. 2023). In contrast to linearization, which is carried out around the model’s fixed point (determined by the intersection of the model’s nullclines), quadratization is done around the extremum (minimum or maximum) of the V-nullcline of quadratic type and the resulting quadratized nullcline is always concave up.

The process of quadratization (Rotstein 2015; Turnquist and Rotstein 2018) consists of expanding the right-side of the (original) biophysical model’s differential equations into Taylor series around the minimum/maximum ($$V_e$$, $$x_{1,e}$$) of the parabolic-like V-nullcline in the subthreshold regime, neglecting all the terms with power bigger than two in the equation for V and bigger than one in the equation for the gating variable $$ x_1 $$, and translating the minimum/maximum of the V-nullcline to the origin. The description of the quadratization process as well as the definition of the quadratized parameters below in terms of the parameters of biophysically realistic (conductance-based) models of Hodgkin-Huxley type are presented in the Appendix A.4. Additional details are provided in Rotstein (2015); Turnquist and Rotstein (2018); Chialva et al. (2023) for 2D and 3D models. A Comparison between the original (biophysical) $$ I_{Nap} $$ + $$ I_h$$ and $$ I_{Nap} $$ + $$ I_{Ks}$$ models (see below) and their quadratization is shown in Fig. 20-A3 and -B3 (Chialva et al. 2023). A detailed description of the process for 3D models of HH type including time-dependent external currents and synaptic inputs is presented in Turnquist and Rotstein (2018) and also discussed in Chialva et al. (2023) in the context of reduced neuronal models.

While the assumption that the V-nullcline is parabolic-like in the subthreshold regime (e.g., Fig. 2 in Turnquist and Rotstein (2018) and Fig. 7 in Chialva et al. (2023)) is a rather general property of the type of models of HH described above, in certain, also rather general parameter regimes, these models can have V-nullclines of cubic-type (Rotstein 2017a, b) around which (not around the parabolic component of the cubic-like nullcline) relevant subthreshold behavior such as subthreshold oscillations are generated.

**Table 2:** Dependence of the linearized conductances $\mathbf {g_L}$ and $\textbf{g}$ on the biophysical parameters $\mathbf {I_{app}}$, $\mathbf {G_p}$, and $\mathbf {G_h}$ / $\mathbf {G_q}$

As X increases		
	$I_{Nap}$ + $I_h$	$I_{Nap}$ + $I_{Ks}$
X = $I_{app}$	$g_L$ decreases (Fig. 8-B1, -C1)	$g_L$ decreases for the higher values of $G_p$ (Fig. 10-B1)
		$g_L$ increases for the lower values of $G_p$ (Fig. 10-C1)
	g decreaes (Fig. 8-B2, -C2)	g increases for all values of $$ G_p$$ (Fig. 10-B2, -C2)
X = $G_h$ / $G_q$	$g_L$ decreases for the higher values of $G_p$ (Fig. 8-B1)	$g_L$ increases for all values of $G_p$ (Fig. 10-B1, -C1)
	$g_L$ increases for the lower values of $G_p$ (Fig. 8-C1)	
	g increases for all values of $G_p$ (Fig. 8-B2, -C2)	g increases for all values of $G_p$ (Fig. 10-B2, -C2)
X = $$ G_p $$	$$ g_L $$ decreases for all values of $$ G_h $$ (Figs. 8-A1)	$$ g_L $$ decreases for all values of $$ G_ q$$ (Fig. 10-A1)
	g decreases for all values of $G_h$ (Figs. 8-A2)	g increases for all values of $G_q$ (Fig. 10-A2)

**Table 3:** Dependence of the properties of the K profile on the biophysical parameters $\mathbf {I_{app}}$, $\mathbf {G_p}$, and $\mathbf {G_h}$ / $\mathbf {G_q}$. When appropriate, the changes in $\Delta \Phi$ are reported in absolute value. * When it exists (there are parameter regimes for which $\Delta \Phi (f)$ is always positive)

As X increases		
	$I_{Nap}$ + $I_h$	$I_{Nap}$ + $I_{Ks}$
X = $$ I_{app} $$	K(f) is amplified (Fig. 9-A1, -B1)	K(f) is amplified for the lower values of $$ G_q $$ and the higher values of $$ G_p$$ (Fig. 11-A1, solid)
		K(f) is attenuated for the higher values of $G_q$ and the higher values of $G_p$ (Fig. 11-A1, dashed)
		K(f) is attenuated for all values of $G_q$ and the lower values of $G_p$ (Fig. 11-B1)
	$$ f_{res,K} $$ decreases (Fig. 9-A1, -B1)	$$ f_{res,K} $$ increases for the higher values of $$ G_p$$ (Fig. 11-A1, -B1)
	$\Delta \Phi (f)$ increases (Fig. 9-A2, -B2)	$\Delta \Phi (f)$ decreases for the lower values of f and increases for the higher values of f (Figs. 11-A2)
	$f_{phas,\Delta \Phi }$decreases (Fig. 9-A2, -B2)	$f_{phas,\Delta \Phi }$ increases* (Fig. 11-A2, -B2)
X = $$ G_h $$ / $$ G_q $$	K(f) is amplified for the higher values of $$ G_p$$ (Figs. 9-C1,solid)	K(f) is attenuated (Fig. 11-C1)
	K(f) is attenuated for the lower values of $G_p$ (Figs. 9-C1, dashed)	
	$f_{res,K}$ first increases and then decreases for the higher values of $G_p$ (Figs. 9-C1,solid)	$f_{res,K}$ increases (Fig. 11-C1)
	$f_{res,K}$ increases for the lower values of $G_p$ (Figs. 9-C1, dashed)	
	$\Delta \Phi (f)$ increases for the higher values of $G_p$ (Figs. 9-C2, solid)	$\Delta \Phi (f)$ decreases (Fig. 11-C2)
	$\Delta \Phi (f)$ decreases for the lower values of $G_p$ (Figs. 9-C2, dashed)	
	$f_{phas,\Delta \Phi }$ increases (Figs. 9-C2)	$f_{phas,\Delta \Phi }$ increases* (Figs. 11-C2)
X = $G_p$	K(f) is amplified (Figs. 9-C1)	K(f) is amplified (Figs. 11-C1)
	$$ f_{res,K}$$ decreases (Figs. 9-C1)	$$ f_{res,K}$$ increases (Figs. 11-C1)
	$\Delta \Phi (f)$ increases (Figs. 9-C2)	$\Delta \Phi (f)$ decreases for the lower values of f and increases for the higher values of f (Figs. 11-C2)
	$f_{phas,\Delta \Phi }$ decreases (Figs. 9-C2)	$f_{phas,\Delta \Phi }$ increases* (Figs. 11-C2)

**Fig. 12:**
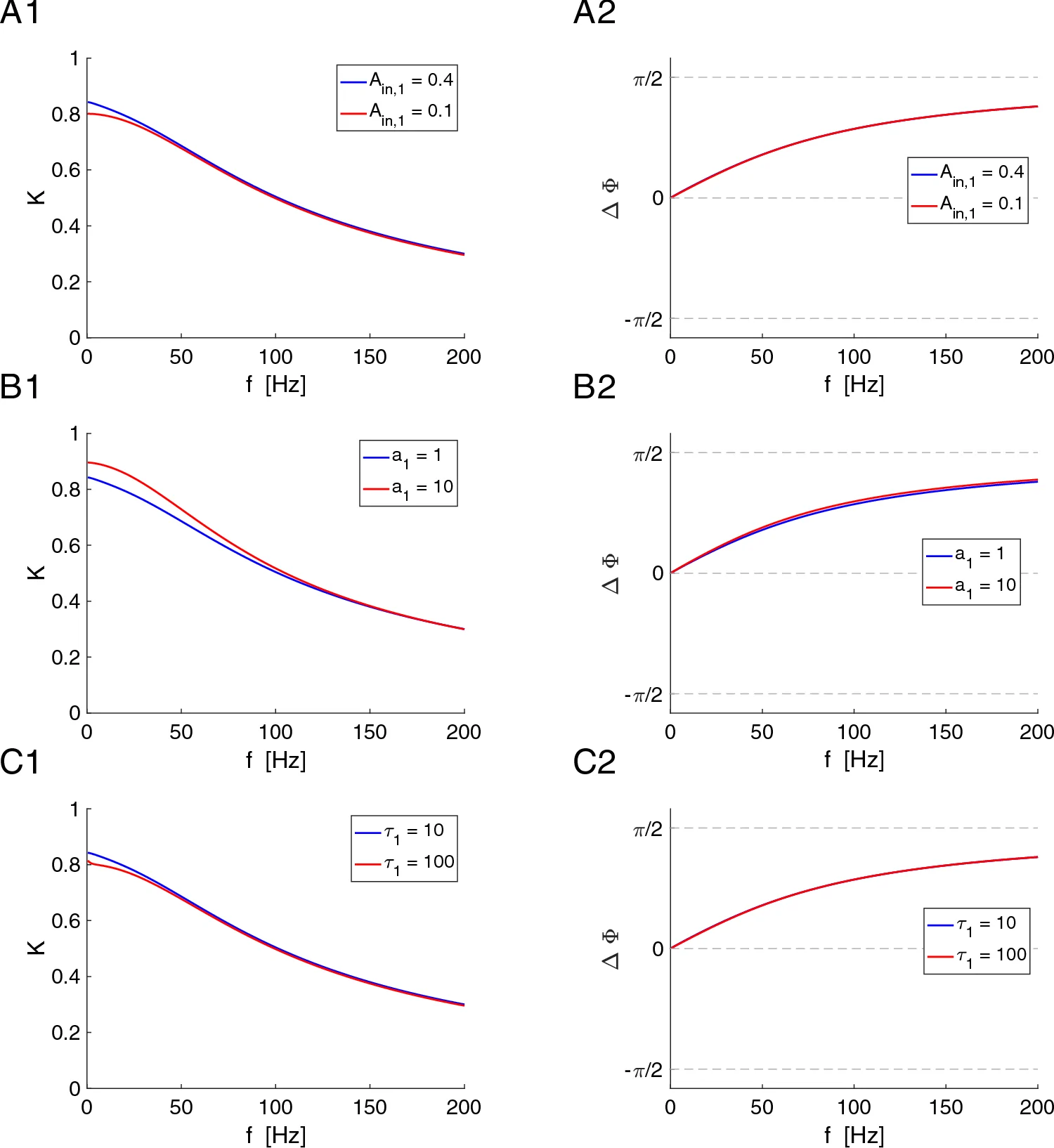
Electrically coupled quadratic 1D model: Effects of the pre-J cell’s properties on the response profiles. We used the model (31) for the electrically coupled pre- and post-J cells with quadratic nonlinearities (cell 1 is the pre-J cell; $$ A_{in,2} = 0 $$) mediated by gap junctions ($$ g_c = 0.5$$). The parameter values for the two cells are identical, except for the ones indicated in the legends. **Left column.** K profiles **Right column.** $$ \Delta \Phi $$ profiles **A.** $$ a_1 = a_2 = 1 $$, $$ \tau _1 = \tau _2 = 10 $$. **B.** $$ \tau _1 = \tau _2 = 10 $$, $$ A_{in,1} = 0.4 $$. **C.** $$ a_1 = 1 = a_2 = 1 $$, $$ A_{in,1} = 0.4 $$. We used the following additional parameter values: $$ I_1 = I_2 = -0.25 $$ and $$ g_c = 4 $$

### The $\mathbf{I_{Nap}}$ + $\mathbf{I_h}$ and $\mathbf{I_{Nap}}$ + $\mathbf{I_{Ks}}$ conductance-based models

The $$I_{Nap} $$ + $$ I_h $$ and $$I_{Nap} $$ + $$ I_{Ks} $$ models (Rotstein 2017a, 2015) are conductance-based models of Hodgkin-Huxley type (Hodgkin and Huxley 1952) in the subthreshold voltage regime that involve the interaction of the two active ionic currents that define them. Both models include two variables: the voltage V and a gating variable associated to the slower $$ I_h $$ or $$ I_{Ks} $$ currents. The dynamics of $$ I_{Nap} $$ is assumed to be instantaneously fast (slaved to voltage). The mathematical formulation is presented in the Appendix A.1 together with the parameter values we use in this paper. The two models are adapted versions of the models presented in Acker et al. (2003); Rotstein et al. (2006) (see also Rotstein (2015, 2017a, 2017b); Turnquist and Rotstein (2018); Chialva et al. (2023)). The parameter values are within the physiological range.

**Fig. 13:**
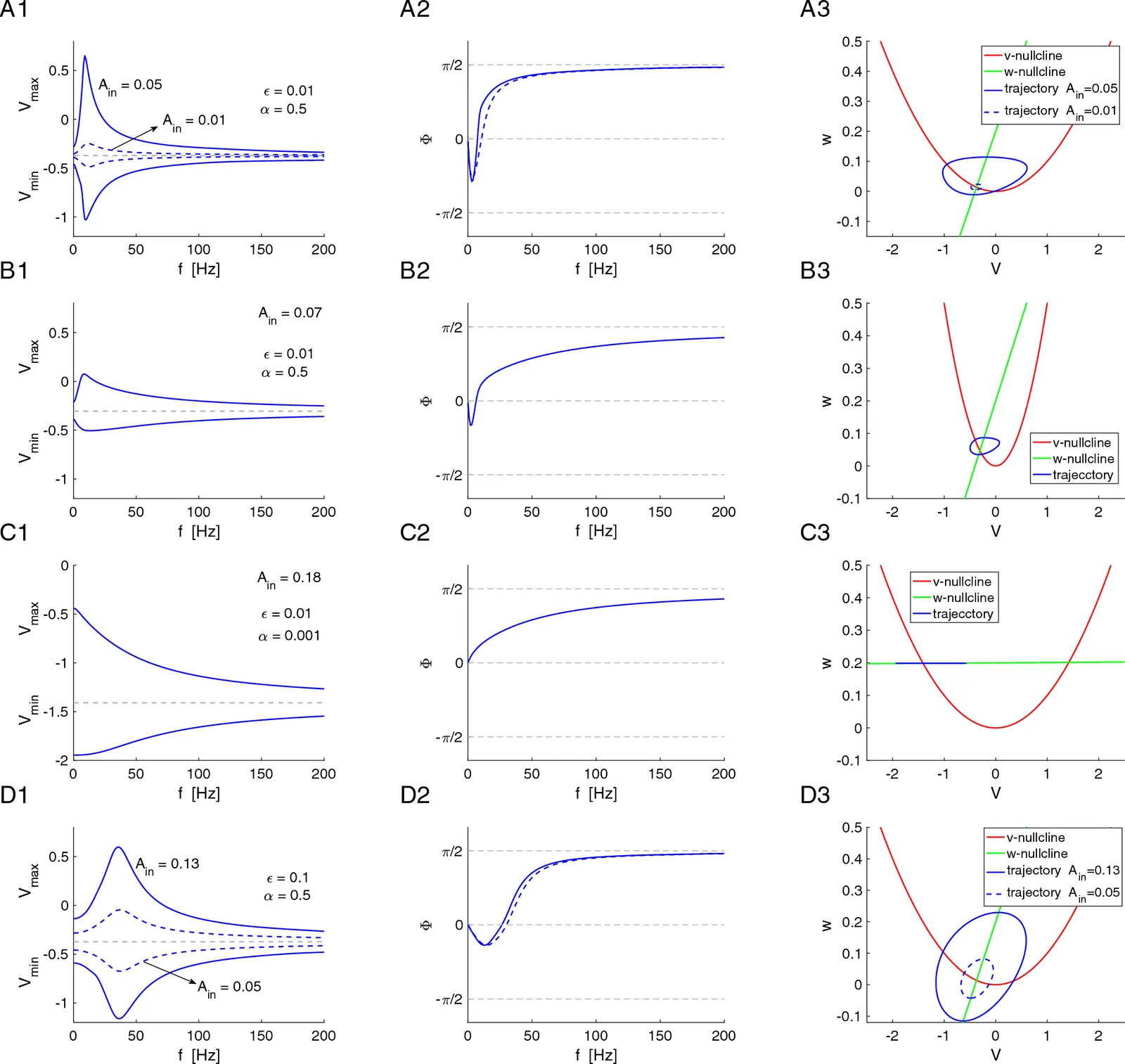
Response of the individual quadratic 2D cells to oscillatory inputs: Representative nonlinear BPFs and LPFs. We used the quadratic model for an individual cell (3)-(4) (k=1, $g_c = 0$). **Left column.** Peak ($V_{max}$) and trough ($V_{min}$) envelope profiles **Middle column.** Phase (Φ) profiles **Right column.** Phase-space diagrams for the peak / resonant frequency. The v- and w-nullclines are given by $w = a\, v^2$ and $w = \alpha \, v - \lambda$, respectively. The response trajectory is a projection of the three-dimensional space onto the v-w plane. **A.** α=0.5, ϵ=0.01, a=0.1, $A_{in} = 0.05$ (solid) and $A_{in} = 0.01$ (dashed). The dashed curve is presented for comparison between the responses of the nonlinear (solid) and quasi-linear (dashed) systems for low enough values of $A_{in}$. The solid curve corresponds to approximately the highest value of $A_{in}$ for which the response remains within the subthreshold regime for all input frequencies.. **B.** α=0.5, ϵ=0.01 a=0.5 and $A_{in} = 0.07$. The parabolic nonlinearity is more pronounced and the response nonlinear. **C.** α=0.001, ϵ=0.01, a=0.1 and $A_{in} = 0.18$. The system is quasi-1D as the result of the disruption of the activation of the negative feedback term. For lower values of $A_{in}$ (e.g., $A_{in} = 0.05$ as the one in panel A), the LPF is quasi-symmetric reflecting the response of a quasi-linear system (not shown). **D.** α=0.5, ϵ=0.1, a=0.1, $A_{in} = 0.13$ (solid) and $A_{in} = 0.05$ (dashed). The BPF is quasi-symmetric reflecting the underlying quasi-linear dynamics. The dashed curve is presented for comparison with the BPF in panel A. We use the following additional parameter value: λ=-0.2

**Fig. 14:**
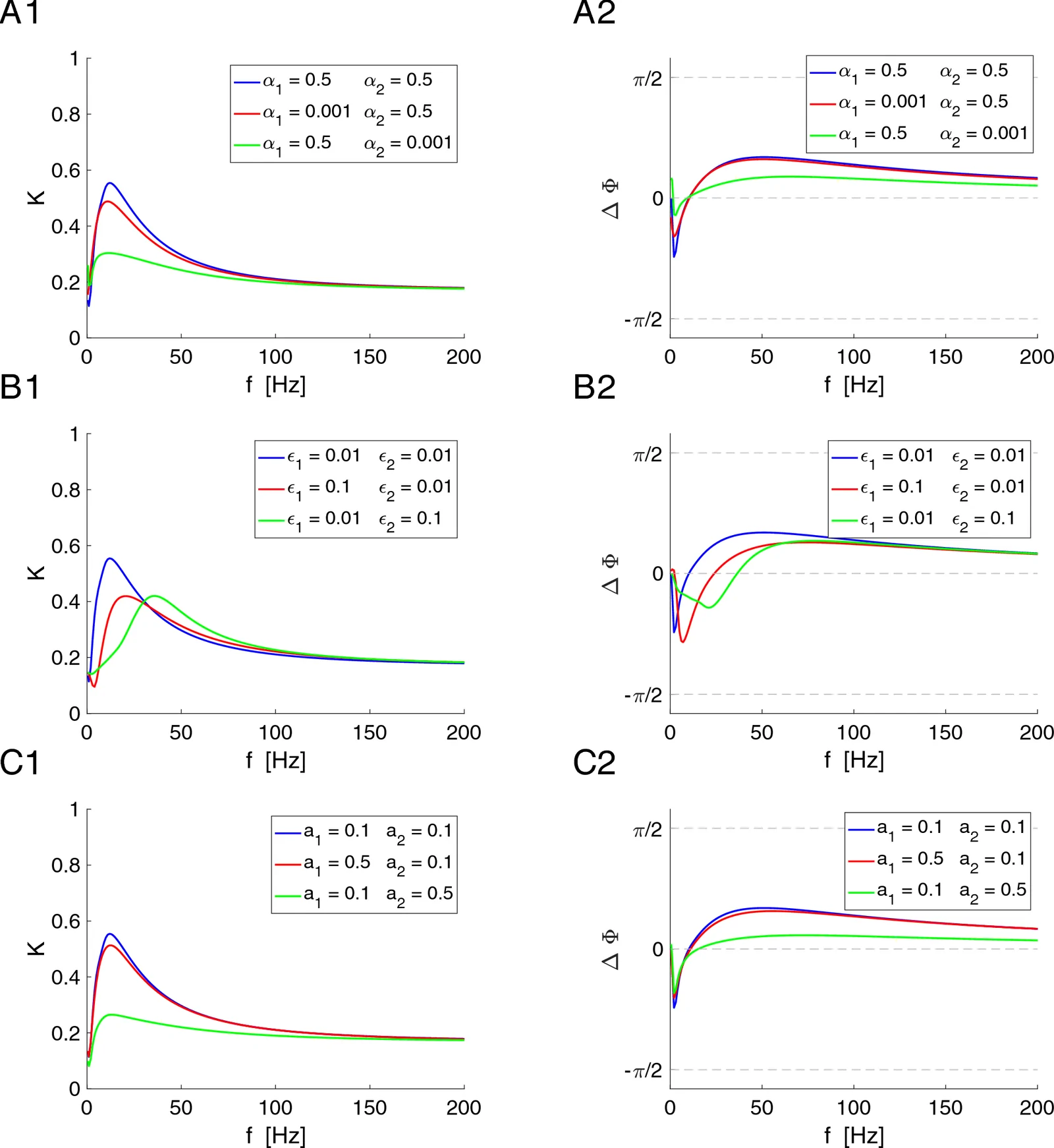
Electrically coupled quadratic 2D model: Effects of the pre-J and post-J cells’ properties on the response profiles. We used the quadratic model (3)-(4) for the electrically coupled pre- and post-J cells with quadratic nonlinearities mediated by gap junctions (cell 1 is the pre-J cell; $A_{in,2} = 0$). The parameter values for the two cells are identical, except for the ones indicated in the legends. **Left column.** K profiles **Right column.** $\Delta \Phi$ profiles **A.** Effect of the α (negative feedback / resonant current strength). We used $a_1 = a_2 = 0.1$, $\epsilon _1 = \epsilon _2 = 0.01$. **A-blue.** The pre-J and post-J cells are identical BPFs (Fig. 13-A). **A-red.** The pre-J cell is a LPF (Fig. 13-C) and the post-J cell is a BPF (Fig. 13-A). **A-green.** The pre-J cell is a BPF (Fig. 13-A) and the post-J cell is a LPF (Fig. 13-C). **B.** Effect of ϵ (negative feedback / resonant current time constant). We used $a_1 = a_2 = 0.1$, $\alpha _1 = \alpha _2 = 0.5$. The pre-J and post-J cells are both BPFs (Figs. 13-A and -D). **B-blue.** The pre-J and post-J cells are identical BPFs (Fig. 13-A). **B-red.** The resonant frequency of the pre-J cell (Fig. 13-D) is higher than for the post-J cell (Fig. 13-A). **B-green.** The resonant frequency of the pre-J cell (Fig. 13-A) is lower than for the post-J cell (Fig. 13-D). **C.** Effect of a (curvature of the parabolic nonlinearity). We used $\alpha _1 = \alpha _2 = 0.5$, $\epsilon _1 = \epsilon _2 = 0.01$. **C-blue.** The V-nullcline for the pre-J and post-J cells have identical curvatures (Fig. 13-A). **C-red.** The curvature of the V-nullcline for the pre-J cell is larger (Fig. 13-B) than for the post-J cell (Fig. 13-A). **C-green.** The curvature of the V-nullcline for the pre-J cell is smaller (Fig. 13-A) than for the post-J cell (Fig. 13-B). We used the following additional parameter values:, $\lambda _1 = \lambda _2 = -0.2$, $g_c = 0.1$, $A_{in,1} = 0.06$ and $A_{in,2} = 0$

**Fig. 15:**
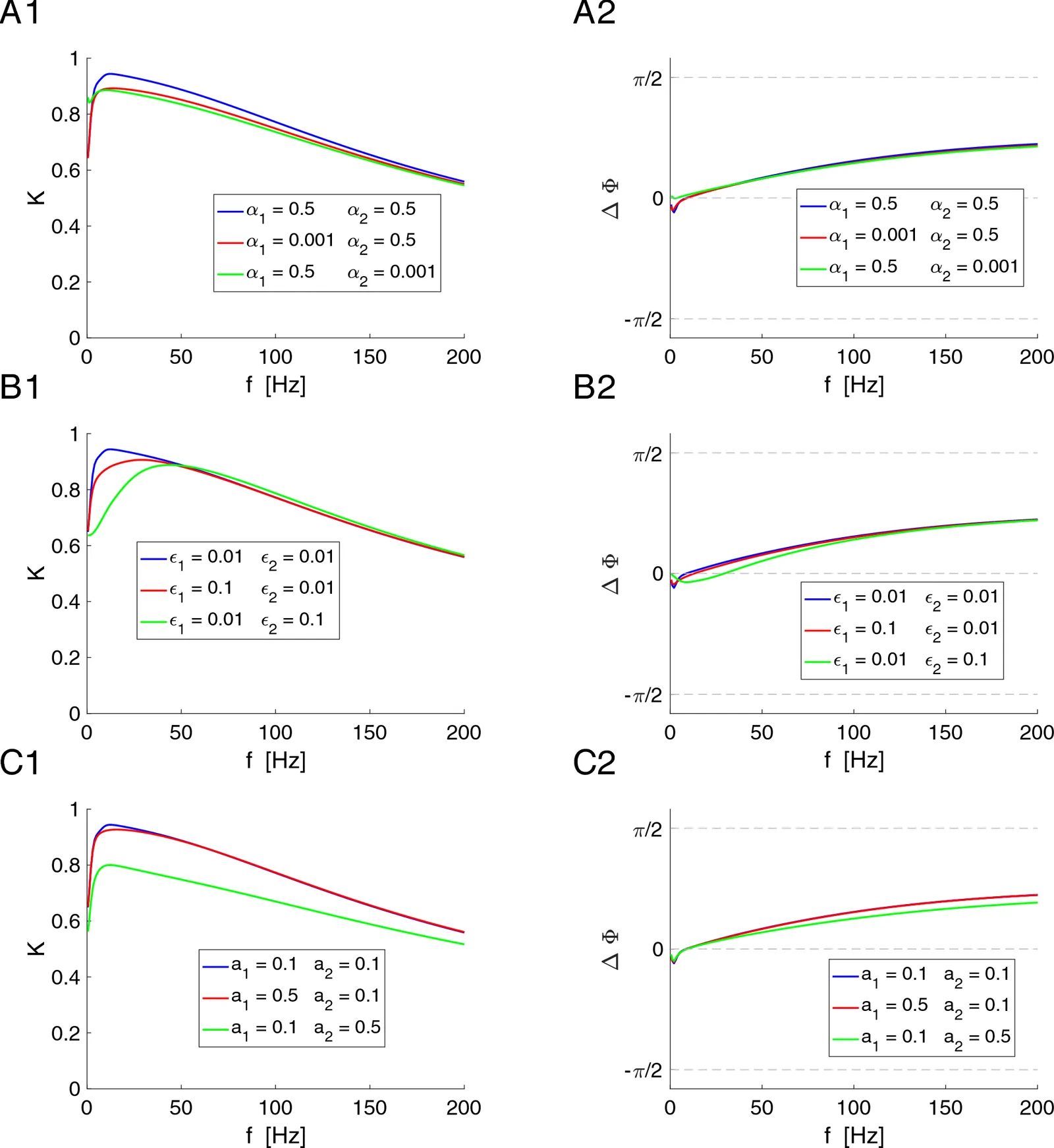
Electrically coupled quadratic 2D model: Effects of the pre-J and post-J cells’ properties on the response profiles. We used the quadratic model (3)-(4) for the electrically coupled pre- and post-J cells with quadratic nonlinearities mediated by gap junctions (cell 1 is the pre-J cell; $A_{in,2} = 0$) . The parameter values for the two cells are identical, except for the ones indicated in the legends. **Left column.** K profiles **Right column.** $\Delta \Phi$ profiles **A.** Effect of the α (negative feedback / resonant current strength). We used $a_1 = a_2 = 0.1$, $\epsilon _1 = \epsilon _2 = 0.01$. **A-blue.** The pre-J and post-J cells are identical BPFs (Fig. 13-A). **A-red.** The pre-J cell is a LPF (Fig. 13-C) and the post-J cell is a BPF (Fig. 13-A). **A-green.** The pre-J cell is a BPF (Fig. 13-A) and the post-J cell is a LPF (Fig. 13-C). **B.** Effect of ϵ (negative feedback / resonant current time constant). We used $a_1 = a_2 = 0.1$, $\alpha _1 = \alpha _2 = 0.5$. The pre-J and post-J cells are both BPFs (Figs. 13-A and -D). **B-blue.** The pre-J and post-J cells are identical BPFs (Fig. 13-A). **B-red.** The resonant frequency of the pre-J cell (Fig. 13-D) is higher than for the post-J cell (Fig. 13-A). **B-green.** The resonant frequency of the pre-J cell (Fig. 13-A) is lower than for the post-J cell (Fig. 13-D). **C.** Effect of a (curvature of the parabolic nonlinearity). We used $\alpha _1 = \alpha _2 = 0.5$, $\epsilon _1 = \epsilon _2 = 0.01$. **C-blue.** The V-nullcline for the pre-J and post-J cells have identical curvatures (Fig. 13-A). **C-red.** The curvature of the V-nullcline for the pre-J cell is larger (Fig. 13-B) than for the post-J cell (Fig. 13-A). **C-green.** The curvature of the V-nullcline for the pre-J cell is smaller (Fig. 13-A) than for the post-J cell (Fig. 13-B). We used the following additional parameter values:, $\lambda _1 = \lambda _2 = -0.2$, $g_c = 1$, $A_{in,1} = 0.08$ and $A_{in,2} = 0$

**Fig. 16:**
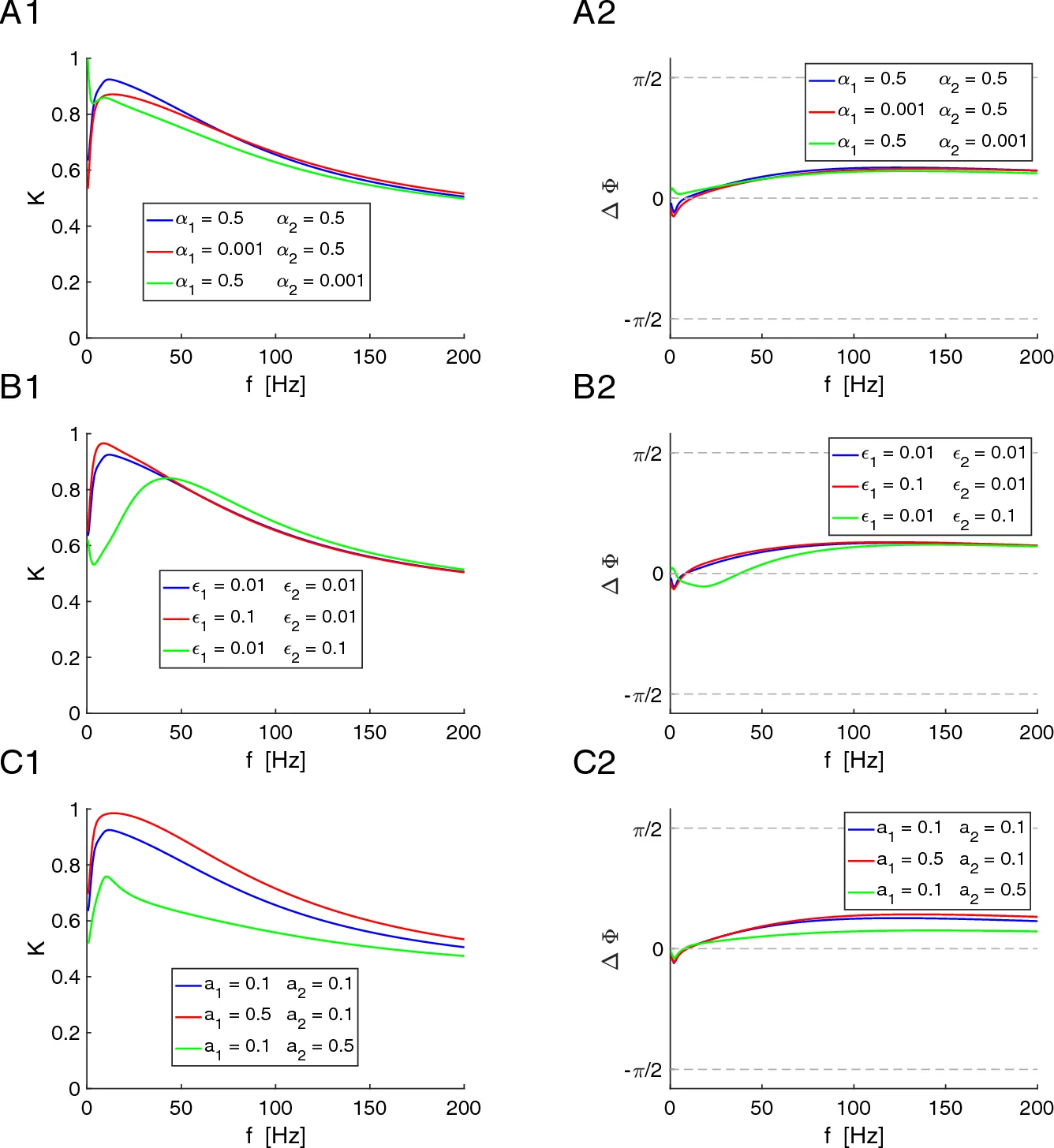
Two-compartment model with quadratic 2D dynamics : Effects of the pre-J and post-J compartments’ properties on the response profiles. We used the quadratic model (3)-(4) for the electrically coupled pre- and post-J compartments with quadratic nonlinearities where the coupling parameter $g_{c,k}$ was substituted by $g_c / \sigma _k$ with $\sigma _1 = 0.8$ and $\sigma _2 = 0.2$ (compartment 1 is the pre-J compartment; $A_{in,2} = 0$) . The parameter values for the two compartments are identical, except for the ones indicated in the legends. **Left column.** K profiles **Right column.** $\Delta \Phi$ profiles **A.** Effect of the α (negative feedback / resonant current strength). We used $a_1 = a_2 = 0.1$, $\epsilon _1 = \epsilon _2 = 0.01$. **A-blue.** The pre-J and post-J compartments are identical BPFs (Fig. 13-A). **A-red.** The pre-J compartment is a LPF (Fig. 13-C) and the post-J compartment is a BPF (Fig. 13-A). **A-green.** The pre-J compartment is a BPF (Fig. 13-A) and the post-J compartment is a LPF (Fig. 13-C). **B.** Effect of ϵ (negative feedback / resonant current time constant). We used $a_1 = a_2 = 0.1$, $\alpha _1 = \alpha _2 = 0.5$. The pre-J and post-J compartments are both BPFs (Figs. 13-A and -D). **B-blue.** The pre-J and post-J compartments are identical BPFs (Fig. 13-A). **B-red.** The resonant frequency of the pre-J compartment (Fig. 13-D) is higher than for the post-J compartment (Fig. 13-A). **B-green.** The resonant frequency of the pre-J compartment (Fig. 13-A) is lower than for the post-J compartment (Fig. 13-D). **C.** Effect of a (curvature of the parabolic nonlinearity). We used $\alpha _1 = \alpha _2 = 0.5$, $\epsilon _1 = \epsilon _2 = 0.01$. **C-blue.** The V-nullcline for the pre-J and post-J cells have identical curvatures (Fig. 13-A). **C-red.** The curvature of the V-nullcline for the pre-J cell is larger (Fig. 13-B) than for the post-J cell (Fig. 13-A). **C-green.** The curvature of the V-nullcline for the pre-J cell is smaller (Fig. 13-A) than for the post-J cell (Fig. 13-B). We used the following additional parameter values:, $\lambda _1 = \lambda _2 = -0.2$, $g_c = 0.05$, $A_{in,1} = 0.06$ and $A_{in,2} = 0$

**Fig. 17:**
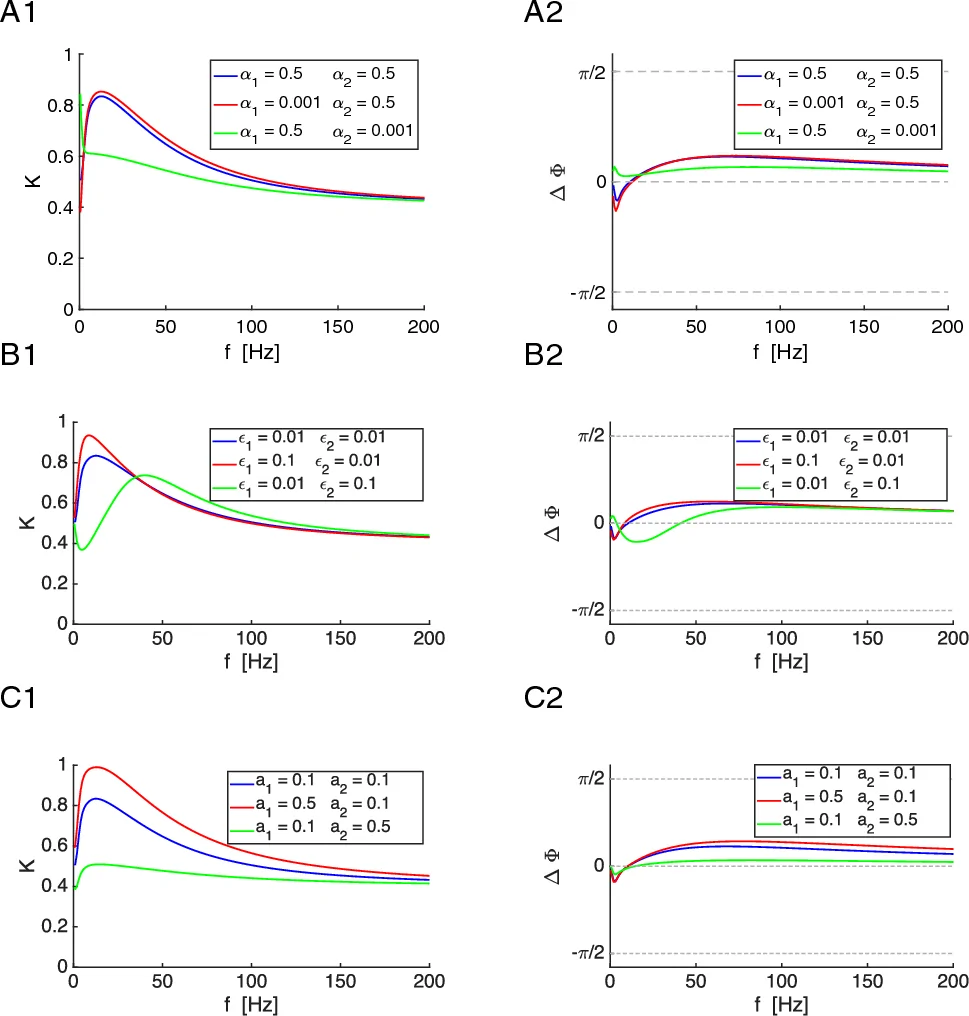
Two-compartment model with quadratic 2D dynamics : Effects of the pre-J and post-J compartments’ properties on the response profiles. We used the quadratic model (3)-(4) for the electrically coupled pre- and post-J compartments with quadratic nonlinearities where the coupling parameter $g_{c,k}$ was substituted by $g_c / \sigma _k$ with $\sigma _1 = 0.2$ and $\sigma _2 = 0.8$ (compartment 1 is the pre-J compartment; $A_{in,2} = 0$) . The parameter values for the two compartments are identical, except for the ones indicated in the legends. **Left column.** K profiles **Right column.** $\Delta \Phi$ profiles **A.** Effect of the α (negative feedback / resonant current strength). We used $a_1 = a_2 = 0.1$, $\epsilon _1 = \epsilon _2 = 0.01$. **A-blue.** The pre-J and post-J compartments are identical BPFs (Fig. 13-A). **A-red.** The pre-J compartment is a LPF (Fig. 13-C) and the post-J compartment is a BPF (Fig. 13-A). **A-green.** The pre-J compartment is a BPF (Fig. 13-A) and the post-J compartment is a LPF (Fig. 13-C). **B.** Effect of ϵ (negative feedback / resonant current time constant). We used $a_1 = a_2 = 0.1$, $\alpha _1 = \alpha _2 = 0.5$. The pre-J and post-J compartments are both BPFs (Figs. 13-A and -D). **B-blue.** The pre-J and post-J compartments are identical BPFs (Fig. 13-A). **B-red.** The resonant frequency of the pre-J compartment (Fig. 13-D) is higher than for the post-J compartment (Fig. 13-A). **B-green.** The resonant frequency of the pre-J compartment (Fig. 13-A) is lower than for the post-J compartment (Fig. 13-D). **C.** Effect of a (curvature of the parabolic nonlinearity). We used $\alpha _1 = \alpha _2 = 0.5$, $\epsilon _1 = \epsilon _2 = 0.01$. **C-blue.** The V-nullcline for the pre-J and post-J cells have identical curvatures (Fig. 13-A). **C-red.** The curvature of the V-nullcline for the pre-J cell is larger (Fig. 13-B) than for the post-J cell (Fig. 13-A). **C-green.** The curvature of the V-nullcline for the pre-J cell is smaller (Fig. 13-A) than for the post-J cell (Fig. 13-B). We used the following additional parameter values:, $\lambda _1 = \lambda _2 = -0.2$, $g_c = 0.05$, $A_{in,1} = 0.06$ and $A_{in,2} = 0$

Both the $$ I_{Nap} $$ + $$I_h$$ and $$ I_{Nap} $$ + $$I_{Ks}$$ models involve the interaction of a restorative current, $$I_h $$ and $$ I_{Ks} $$, having a resonant gating variable, and the same regenerative current, $$ I_{Nap} $$, having an amplifying gating variable. Because all these currents have a single gating variable, we refer to them as resonant and amplifying currents, accordingly. They provide a positive ($$ I_{Nap} $$) and slower negative ($$I_h $$ and $$ I_{Ks} $$) feedback effects, favoring and opposing changes in voltage, respectively.

The two models have V-nullclines of quadratic type or are quasi-linear in the subthreshold regime for the parameter sets considered in this paper (Figs. 20-A1 and -B1) (see also Turnquist and Rotstein (2018), Fig. 2, and Chialva et al. (2023), Fig. 7, Bel et al. (2025), Figs. Figs. S4 and S5). Note that despite their biophysical differences, the nullclines are qualitatively a mirror image of each other: the V-nullcline is concave down for the $$I_{Nap} $$ + $$ I_h $$ model (Fig. 20-A1) and and concave up for the $$I_{Nap} $$ + $$ I_{Ks} $$ model (Figs. 20). The quadratization process (see Section A.4) uncovers the qualitative similarity of the phase-plane diagrams for both models (Fig. 20). The model describes the onset of spikes. When the models are supplemented with a return mechanism to the subthreshold regime, they are referred to as models of quadratic integrate-and-fire type with adaptation (Chialva et al. 2023; Izhikevich 2006, 2010). In other parameter regimes (e.g., lower levels of $$ I_{Nap}$$), the two $$I_{Nap} $$ + $$ I_h $$ and $$I_{Nap} $$ + $$ I_{Ks} $$ models can be quasi-linear (Bel et al. 2025) or have cubic nonlinearities (Rotstein 2017a).

**Fig. 18:**
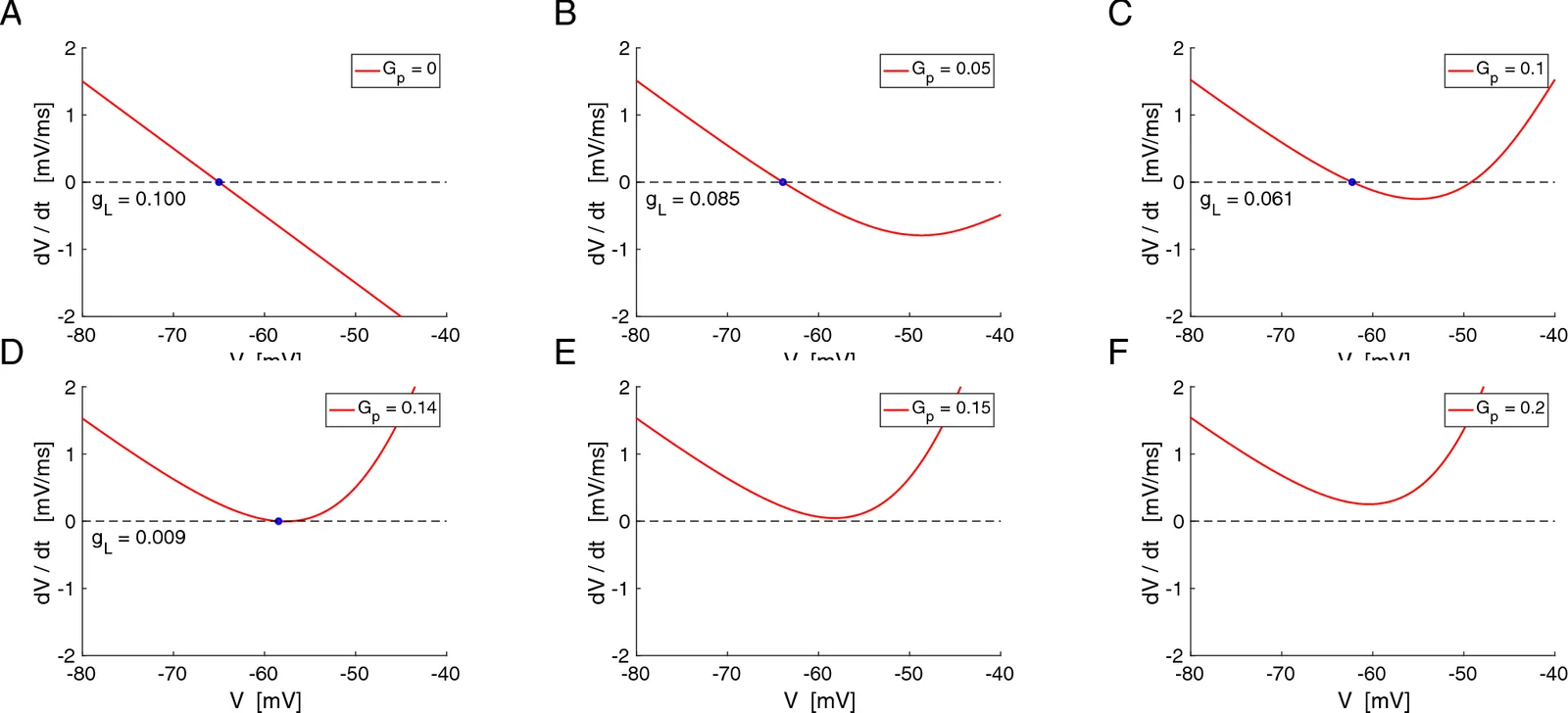
Phase-space diagrams for the $I_{Nap}$ model (subthreshold regime) for representative parameter values. dV/dt vs. V curves for representative parameter values of $G_p$ and $G_L$ and $I_{app} = 0$. We used the parameter values presented in the Appendix A.2. This model can be thought of as a 1D reduced version of the 2D models illustrated in Fig. 20. See additional details there. The trajectories (not shown) move along the V axis and converge to the stable fixed points (blue dots) (A - D) or escape the subthreshold regime (E, F). For $G_p = 0$ the modes is a passive cell, which is linear (A). As $G_p$ increases, nonlinearities of parabolic type develop and become more pronounced in a vicinity of the stable fixed-point (B-D). The unstable fixed-point serves as a threshold for spike generation. If the model is supplemented with a return mechanism to the subthreshold regime, it becomes a model of quadratic integrate-and-fire type (Chialva et al. 2023). For the saddle-node bifurcation value of $G_p$, the fixed-points cease to exist (E, F). The associated quadratic integrate-and-fire model produces periodic spiking

The $I_{Nap}$ model is a 1D reduced version of the 2D models discussed above, which does not include any slow ionic current. It is described in Section A.2 by eqs. (A-1) with $I_1 = 0$ and $I_2 = I_{Nap}$ eq. (A-3) and its dynamics are illustrated in Fig. 18. Analogously to the other two models, it has parabolic-like or quasi-linear speed (dV/dt vs. V) curve in the subthreshold regime and it describes the onset of spikes. When supplemented with a return mechanism to the subthreshold regime it becomes a model of quadratic integrate-and-fire type (Chialva et al. 2023) (the well-known quadratic integrate-and-fire model (Latham et al. 2000; Hansel and Mato 2001) has a purely parabolic speed curve).

### Single cell voltage response to oscillatory inputs: low- and band-pass filters

The voltage response of a neuron receiving an oscillatory input current (with frequency f) is typically measured in terms of the impedance $\textbf{Z}(f)$, a complex quantity with amplitude $Z(f) = |\textbf{Z}(f)|$ and phase Φ(f). Following others, we refer to Z(f) simply as the impedance. Passive cells are low-pass filters (LPFs; e.g., Figs. 1-A1) and exhibit a lagged response (e.g., Figs. 1-C1). More complex cells can be band-pass filters (BPFs; e.g., Figs. 1-A2) and exhibit a lagged/leading response (e.g., Figs. 1-C2). A system exhibits *resonance* if Z(f) peaks at a non-zero (resonant) frequency $f_{res}$ (e.g., Figs. 1-A2) and *phasonance* if Φ(f) vanishes at a non-zero (phasonant) frequency $f_{phas}$ (e.g., Figs. 1-C2).

The generation of cellular resonance requires the interplay of negative and positive feedback effects mediated by the gating variables associated to the intrinsic, resonant (e.g., $$ I_h $$, $$ I_{Ks}$$) and amplifying ($$I_{Nap}$$) ionic currents, respectively.

The resonant properties of 2D linear and nonlinear neuronal systems, including their relationship between the cellular intrinsic biophysical properties and the dynamic mechanism of generation of resonance have been investigated extensively by us and other authors Richardson et al. (2003); Rotstein and Nadim (2014); Rotstein (2014, 2015). In the Appendix B we present the analytical expressions of Z(f) and Φ(f) for linear (1D and 2D) neuronal models receiving a sinusoidal input current of the form

5 $$\begin{aligned} I_{in}(t) = A_{in}\, \sin (\Omega \, t) \ \ \ \ \ \ \ \ \ \ \text{ with } \ \ \ \ \ \ \ \ \ \ \Omega = \frac{2 \pi f}{1000}. \end{aligned}$$

For nonlinear systems we define the peak envelope impedance profile (Fig. 1-B) (Pena and Rotstein 2022a; Rotstein 2014) as

6 $$\begin{aligned} Z^{ENV}(f) = \frac{V_{max}(f)-V_{min}(f)}{2 A_{in}} \end{aligned}$$

where $V_{max}(f)$ and $V_{min}(f)$ are the peak and trough envelope profiles, respectively, defined as the maximum and minimum values, respectively, of the steady-state oscillatory voltage $V_{out}(f)$ as a function of the input frequency f. For linear systems, $Z^{ENV}(f) = Z(f)$. Similarly, the phase Φ is computed as the distance between the peaks of the output and closest input normalized by period, and $f_{phas}$ is the non-zero frequency where Φ vanishes. These quantities extend the concept of impedance and phase to nonlinear systems under certain conditions (the input and output frequencies coincide and the output amplitude is uniform across cycles for a given input with constant amplitude).

### Network voltage response to oscillatory inputs

We now consider a two-cell network (Fig. 2) receiving sinusoidal inputs of the form (5) $I_{in,k}(t)=A_{in,k} \sin (\Omega \, t)$ (k=1,2). In this paper, we consider inputs to only one cell (cell 1); i.e., $I_{in,1}(t)=A_{in} \sin (\Omega \, t)$ and $I_{in,2}(t)=0$. For generality, in the Appendix C we provide the calculations using linear networks of N cells, each having 2D linear dynamics and sinusoidal inputs to all cells in the network.

#### Networks of linear nodes

For networks having linear nodes and linear connectivity, the voltage response of each cell has the form $V_{out,k}(t;f)=A_k(f)\sin (\Omega \, t - \Phi _{k}(f))$. The amplitude $A_k(\Omega )$ and phase $\Phi _{k}(\Omega )$ are the component of the complex network voltage amplitude response $\textbf{A}_k(f)$ derived in the Appendix C

7 $$\begin{aligned} \textbf{Z}_{1}(\Omega ) = \frac{\mathbf{A_1}(\Omega )}{A_{in,1}} = \frac{1}{1 - g_c^2\, {\hat{\textbf{Z}}_1^0}(\Omega )\, {\hat{\textbf{Z}}_2^0}(\Omega )}\, {\hat{\textbf{Z}}_1^0}(\Omega ) \end{aligned}$$

and

8 $$\begin{aligned} \textbf{Z}_{2}(\Omega ) = \frac{\mathbf{A_2}(\Omega )}{A_{in,1}} = \frac{g_c\, {\hat{\textbf{Z}}_1^0}(\Omega )}{1 - g_c^2\, {\hat{\textbf{Z}}_1^0}(\Omega )\, {\hat{\textbf{Z}}_2^0}(\Omega )}\, {\hat{\textbf{Z}}_2^0}(\Omega ), \end{aligned}$$

where $${\hat{\textbf{Z}}_k^0}(\Omega )$$ (k=1,2) are the extended (complex) impedances of the individual (disconnected) nodes in the network obtained from eq. (A-21) by substituting the parameters $$ g_{L,k} $$ by $$ g_{L,k} + g_c$$ (Bel et al. 2025)

9 $$\begin{aligned} {\hat{\textbf{Z}}}_k^0(f) = \frac{\textbf{Q}_k(\Omega )}{\textbf{P}_{k,c}(\Omega )} \end{aligned}$$

where

10 $$\begin{aligned} \textbf{P}_{k,c}(\Omega )&= P_k(\Omega ;g_c) = g_{L,k} + g_k + g_c - C_k \tau _k \Omega ^2 \nonumber \\&\quad + i\, \Omega \, (g_{L,k} \tau + g_c \tau + C_k) \end{aligned}$$

and

11 $$\begin{aligned} \textbf{Q}_k(\Omega ) = 1 + i\, \Omega \, \tau _k, \end{aligned}$$

for k=1,2. This captures the fact that the gap junction current includes a “self-connectivity" term, which is incorporated into the autonomous part of the corresponding nodes k.

#### Networks of nonlinear nodes: network impedance and peak envelope profiles

For nonlinear networks we adapt the tools defined in the previous section by adding the appropriate indices and referring to the corresponding quantities for the individual (disconnected) cells by adding a superscript "0". In contrast to linear systems, the voltage response for nonlinear systems is not symmetric. Therefore, while the impedance amplitude captures the frequency content of the voltage response signal, it does not necessarily describe the frequency-dependent prope rties of the peak and trough envelope profiles. Because neuronal signal occurs via voltage threshold-like mechanisms, we will look at the peak envelope profiles $V_{max,k}(f)$ for k=1,2

### Coupling coefficient: Frequency-dependent profiles and preferred communication frequencies

#### Amplitude and phase

We extend the notion of the (steady state) coupling coefficient in response to constant (DC) inputs (Shimizu and Stopfer 2013, García-Perez et al. 2004, Hoge et al. 2011, Pereda et al. 1828, Gibson et al. 2005) to include the frequency-dependent effects in response to oscillatory inputs by defining the amplitude coupling coefficient

12 $$\begin{aligned} K(f) = \frac{V_{max,2}(f)-\bar{v}_2}{V_{max,1}(f)-\bar{v}_1} \end{aligned}$$

where $(\bar{v}_1,\bar{v}_2)$ is the fixed point of system (1)-(2), when unperturbed and the phase-difference coupling coefficient

13 $$\begin{aligned} \Delta \Phi (f) = \Phi _{2}(f)-\Phi _{1}(f). \end{aligned}$$

For linear systems ($\bar{v}_1 = \bar{v}_2 = 0$), K(f) and $\Delta \Phi (f)$ are the components of the following complex quantity

14 $$\begin{aligned} \textbf{K}(f)&= \frac{\textbf{Z}_{2}(f)}{\textbf{Z}_{1}(f)} = \frac{Z_{2}(f)}{Z_{1}(f)}\, \, e^{-i\, [\, \Phi _{2}(f) - \Phi _{1}(f)\, ]} \nonumber \\&= \frac{A_{2}(f)}{A_{1}(f)}\, \, e^{-i\, [\, \Phi _{2}(f) - \Phi _{1}(f)\, ]} = K(f)\, e^{-i\, \Delta \Phi (f)}. \end{aligned}$$

From (7)-(8),

15 $$\begin{aligned} K(f) = g_c\, \hat{Z}_2^0(f) \text{ and } \Delta \Phi = \hat{\Phi }_2^0(f). \end{aligned}$$

#### K-resonance and $\Delta \Phi$-phasonance

We define K-resonance as the ability of the coupling coefficient K(f) to exhibit a peak at a non-zero input frequency $f_{res,K}$, and

$\Delta \Phi$-phasonance as the ability of the frequency-dependent coupling phase $\Delta \Phi (f)$ to vanish for a nonzero input frequency $f_{phas,\Delta \Phi }$.

From eq. (12), a necessary condition for the existence of K-resonance (or K-antiresonance) is given by

16 $$\begin{aligned} V'_{max,2}(f)(V_{max,1}(f){-}\bar{v}_1){-}V'_{max,1}(f)(V_{max,2}(f){-}\bar{v}_2){=}0, \end{aligned}$$

for some value f>0.

We characterize the K(f) profile by using two attributes: (i) the resonant frequency $$f_{res,K}$$, defined as the peak frequency of K(f), and (ii) the resonance amplitude $$Q_K = K_{max} - K(0)$$. If the K profile does not exhibit resonance, then $$f_{res,K} = Q_K = 0 $$. We characterize the $$ \Delta \Phi (f) $$ profile by using the phasonant frequency $$ f_{phas,\Delta \Phi } $$.

### Electrically coupled neuronal compartments

The mathematical formulation of two-compartment models of spatially extended cells (e.g., dendritic, somato-dendritic) having linear dynamics is as described in Sections 2.1 and 2.2 with $g_c$ substituted by $g_c / \sigma _k$ (k=1,2) with $\sigma _1 + \sigma _2 = 1$, where $\sigma _k$ represents the fraction of the total cell area taken up by the corresponding compartment (Pinsky and Rinzel 1994; Kepecs et al. 2002, 2003). For linearized models, because of the possible asymmetry between the two compartments, there is a constant membrane potential effect due to the differences in the resting potential of the two compartments.

For two-compartment models of linear nodes where the input arrives only to one of the compartments (compartment 1), the complex network voltage amplitude response presented in Section 2.5.1 extends to (see also Section C)

17 $$\begin{aligned} \mathbf{A_1}(\Omega ) = \frac{\sigma _1 \sigma _2}{\sigma _1 \sigma _2 - g_c^2\, {\hat{\textbf{Z}}_1^0}(\Omega )\, {\hat{\textbf{Z}}_2^0}(\Omega )}\, {\hat{\textbf{Z}}_1^0}(\Omega )\, A_{in,1} \end{aligned}$$

and

18 $$\begin{aligned} \mathbf{A_2}(\Omega ) = \frac{g_c\, \sigma _1\, {\hat{\textbf{Z}}_1^0}(\Omega )}{\sigma _1 \sigma _2 - g_c^2\, {\hat{\textbf{Z}}_1^0}(\Omega )\, {\hat{\textbf{Z}}_2^0}(\Omega )}\, {\hat{\textbf{Z}}_2^0}(\Omega )\, A_{in,1}, \end{aligned}$$

where ${\hat{\textbf{Z}}_k^0}(\Omega ) = {\hat{\textbf{Z}}_k^0}(\Omega ;g_c/\sigma _k)$ (k=1,2) are the extended (complex) impedances of the individual (disconnected) nodes in the network obtained from eq. (A-21) in the Appendix B by substituting the parameters $g_{L,k}$ by $g_{L,k} + g_c / \sigma _k$. This can be straightforwardly extended to more detailed formulations involving the geometric (length, area) and biophysical properties of neuronal compartments and a larger number of compartments (Dayan and Abbott 2001; Ermentrout and Terman 2010).

It follows that

19 $$\begin{aligned} K(f;g_c/\sigma _2)&= \frac{g_c}{\sigma _2}\, \hat{Z}_2^0(f;g_c/\sigma _2) \nonumber \\ \text{ and } \Delta \Phi (f;g_c/\sigma _2)&= \hat{\Phi }_2^0(f;g_c/\sigma _2). \end{aligned}$$

### Numerical simulations

To compute the numerical solutions we used a Runge-Kutta method of order 4 (Burden and Faires 1980) with a time step Δt=0.01 ms. Smaller values of Δt have been used to check the accuracy of the results. All neural models and metrics, including phase-plane analysis, were implemented in MATLAB (The Mathworks, Natick, MA). The codes are available at https://github.com/BioDatanamics-Lab/Coupling_Coefficient-25_05.

## Results

### Steady-state coupling coefficient K in response to constant inputs

#### Linear models: revisited

The steady-state response of the electrically coupled linear cells (1)-(2) to a constant (DC) inputs $I_1$ ($I_2= 0$) is given by

20 $$\begin{aligned} \bar{v}_1 = \frac{(g_{L,2}+g_2+g_c)\, I_1}{(g_{L,1}+g_1+g_c)\, (g_{L,2}+g_2+g_c) - g_c^2} \end{aligned}$$

and

21 $$\begin{aligned} \bar{v}_2 = \frac{g_c\, I_1}{(g_{L,1}+g_1+g_c)\, (g_{L,2}+g_2+g_c) - g_c^2} \end{aligned}$$

for the pre-J and post-J cells, respectively.

The coupling coefficient is therefore given by

22 $$\begin{aligned} K = \frac{v_2}{v_1} = \frac{g_c}{g_{L,2}+g_2+g_c}. \end{aligned}$$

As reported before for networks of passive cells ($g_2 = 0$) (Curti and O’brien 2016; Curti et al. 2022), K is independent of the input current and the intrinsic properties of the pre-J cell, and varies between 0 and 1. For networks of 2D linear cells, K is affected by $g_2$ in addition to$g_{L,2}$ and it remains independent of the intrinsic properties of the pre-J cell.

Note that if the connectivity coefficients are different, as it is usually the case for two-compartment models, then

23 $$\begin{aligned} \bar{v}_1 = \frac{(g_{L,2}+g_2+g_{c,12})\, I_1}{(g_{L,1}+g_1+g_{c,12})\, (g_{L,2}+g_2+g_{c,21}) - g_{c,12} g_{c,21}} \end{aligned}$$

and

24 $$\begin{aligned} \bar{v}_2 = \frac{g_{c,12}\, I_1}{(g_{L,1}+g_1+g_{c,12})\, (g_{L,2}+g_2+g_{c,21}) - g_{c,12} g_{c,21}} \end{aligned}$$

for the pre-J and post-J cells, respectively, and

25 $$\begin{aligned} K = \frac{v_2}{v_1} = \frac{g_{c,21}}{g_{L,2}+g_2+g_{c,21}}. \end{aligned}$$

#### Quadratic models

Our analytical results for networks of weakly nonlinear cells (Appendix D) show that as soon as nonlinearities are present, K is no longer independent of $I_1$ and is (weakly) affected by the intrinsic properties of both the pre- and post-J cells (Appendix D.1). Here, we investigate these dependencies for the quadratic model (3)-(4).

The dynamics of the quadratic model is illustrated in Fig. 3. Changes in the values of the constant (DC) input current are monotonically reflected in the parameter λ. In our simulations, we first determined a baseline value of λ, $\lambda _{base}$ and computed the stationary voltage response $v_{ss,base}$ of the individual cells to this value. We then computed λ as $\lambda = \lambda _{base} + \Delta \lambda$ and used $v_{ss,base}$ as the initial condition in our simulations (blue dots in Fig. 3, right column).

Fig. 4-A illustrates the response of the pre-J ($v_1$) and post-J ($v_2$) cells for values of $g_c$ that increase from panel A1 to panel A2. Fig. 4-B shows curves of K as a function of $\Delta \lambda$ for representative scenarios. In contrast to linear models, there is a dependence of K on λ, which is more pronounced for the larger values of $g_c$. Comparison between panels B1 and B2 illustrate that the dependence of K on λ is independent on ϵ. Comparison between panels B1 and B3 illustrate that the dependence of K on λ is more pronounced and the values of K are larger the smaller α (all other parameters fixed). Finally, comparison between panels B1 and B4 illustrate that the dependence of K on λ is slightly more pronounced and the values of K are larger the smaller a (all other parameters fixed).

### Response to oscillatory inputs: Electrically coupled linear cells

Linear models of single neurons exhibit a variety of amplitude and phase profiles in response to oscillatory inputs (e.g., Fig. 1) (Hutcheon and Yarom 2000; Richardson et al. 2003; Rotstein and Nadim 2014; Rotstein 2017, 2018a, b) (see Appendix B) as the result of the interaction of negative and positive feedback effects. However, purely linear models are agnostic to the underlying biological process. Neuronal linearized models (see Appendix A.3) encode information about the inherently nonlinear biophysical properties of neurons and networks and provide a biophysical interpretation to the linear model parameters and the emerging neuronal linear filters (Rotstein and Nadim 2014).

Here we analyze the response of electrically coupled linear cells to oscillatory inputs. We use these results in the next section to analyze, to the linearized level of description, the dependence of the K(f) and $\Delta \Phi (f)$ on the biophysical ionic currents that provide the necessary negative and positive feedback effects.

For purely linear networks (linear nodes and linear connectivity) of the form (1)-(2), the network complex amplitude response to oscillatory inputs for the pre-J ($\textbf{A}_1$) and post-J ($\textbf{A}_2$) cells are given by eqs. (7) and (8), respectively (see Appendix C for details on the formulas and their derivation). The numerators describe the response of the directly activated pre-J cell and the indirectly activated post-J cell, respectively, modulated by the presence of the gap junction via de extended impedances (${\hat{\textbf{Z}}}_1$ and ${\hat{\textbf{Z}}}_2$). The denominators, describe the interaction between the two cells in response to the oscillatory input.

From eqs. (15), the CC K(f) for linear systems is proportional to the extended impedance of the post-J cell and the phase-difference coefficient $\Delta \Phi (f)$ is equal to the extended phase of the post-J cell. Both quantities are independent of the input amplitude ($A_{in}$) to the pre-J cell and of the intrinsic properties of the pre-J cell. While the formulas are valid for both cells, here we focus on $g_{L,2}$ and $g_2$ for the post-J cell since the K(f)- and $\Delta \Phi (f)$-profiles depend on these parameters.

#### Passive cells

For two electrically coupled passive cells (1)-(2) ($$ g_k = 0, k = 1, 2$$) (Fig. 2-A), the coupling and phase-difference coefficient profiles are given, respectively, by

26 $$\begin{aligned} K(f;g_c) = \frac{g_c}{\sqrt{(g_{L,2}+g_c)^2+\Omega ^2 }} \end{aligned}$$

and

27 $$\begin{aligned} \Delta \Phi (f;g_c) = \tan ^{-1} \left( \frac{\Omega }{g_{L,2}+g_c} \right) . \end{aligned}$$

The K(f)-profiles are LPFs (Fig. 5, left) and the $\Delta \Phi (f)$ profile are monotonically increasing (Fig. 5, right), indicating that the lag difference between the two cells is monotonically increasing. Therefore, the results from this Section remain true when the pre-J cells is a resonator (Fig. 2-D).

Fig. 5-A illustrates the dependence of the K(f)- and $\Delta \Phi (f)$-profiles on $g_c$ and $g_{L,2}$. The network A-profiles (individual cells) are LPFs (Fig. 5-A1). Increasing values of $g_{L,2}$ (for fixed values of $g_c$) attenuate the A-profiles and make them shallower. Increasing values of $g_c$ (for fixed values of $g_{L,2}$) also make the K(f)-profiles shallower, but amplify them, as expected. In the first case, the recruitment of the post-J cell is stronger, which in turn weakens the response of the pre-J cell (Fig. 5-B1), while in the second case, the response of the two cells is weaker as $g_{L,2}$ increases (Fig. 5-C1).

The network Φ-profiles are monotonically increasing (Fig. 5-A2). Increasing values of $g_{L,2}$ (for fixed values of $g_c$) and increasing values of $g_c$ (for fixed values of $g_{L,2}$) reduce the rate of increase of the $\Delta \Phi (f)$ profiles, indicating that the response oscillations’ peaks for the two cells are closer the larger the input frequency. The stronger recruitment of the post-J cell as $g_c$ increases (Fig. 5-B1) is accompanied by a reduction in both the network phases and phase-difference for each input frequency f (Fig. 5-B2). A similar effect is produced by increasing values of $g_{L,2}$.

#### Linear (2D) resonators: Interplay of positive and negative feedback effects

Resonators refer to cells that exhibit resonance (Hutcheon and Yarom 2000). For simplicity, here, we include in this category cells that exhibit resonance both in the absence or presence of damped subthreshold oscillations (Richardson et al. 2003; Rotstein and Nadim 2014) where the fixed-points are nodes and foci, respectively (i.e., we do not distinguish between resonators and damped oscillators). Resonators may also show phasonance (Richardson et al. 2003; Rotstein and Nadim 2014). Resonance, phasonance and oscillations require the interplay of positive and slower negative feedback effects that favor and oppose changes of voltage, respectively. In the linearized models (1)-(2), the negative feedback is represented by $$ g_k > 0$$ (k=1,2) and the positive feedback is embedded in the parameters $$ g_{L,k}$$.

For two electrically coupled 2D linear cells (1)-(2) ($$ g_k > 0, k = 1, 2$$) (Fig. 2-B), the coupling and phase-difference coefficient profiles are given, respectively, by

28 $$\begin{aligned} K(f;g_c) = g_c\, \sqrt{\frac{1+\tau _2^2\, \Omega ^2}{(g_{L,2} + g_2 + g_c - \tau _2\, \Omega ^2)^2 + (g_{L,2}\, \tau _2 + g_c \tau _2+ 1)^2\, \Omega ^2}} \end{aligned}$$

and

29 $$\begin{aligned} \Delta \Phi (f;g_c) = \tan ^{-1}\left( \frac{1-\tau _2 g_2 +(\tau _2\, \Omega )^2}{g_{L,2} + g_2 +g_c +(g_{L,2}+g_c) (\tau _2\, \Omega )^2 }\Omega \right) . \end{aligned}$$

Fig. 6 illustrates the K(f) and $\Delta \Phi (f)$ profiles for representative scenarios. We focus on individual cells that exhibit resonance (e.g., Fig. 6-D1, dashed-blue) and phasonance (e.g., Fig. 6-D2, dashed-blue). The K(f) profiles are BPFs (Fig. 6-A1 to -C1), exhibiting K-resonance, and the $\Delta \Phi (f)$ profiles show $\Delta \Phi$-phasonance (e.g., Fig. 6-A2 to C2) in most cases considered. As mentioned above, these profiles are determined by the parameter of the post-J cell and $g_c$, and are independent of the input amplitude and the properties of the pre-J cell.

Figs. 6-A1 to -C1 show the dependence of the K(f)-profiles on the resonant linearized conductance $g_2$, the time constant $\tau _2$ of the resonant gating variable, the linearized leak conductance $g_{L,2}$ and the gap junction coefficient $g_c$. Increasing values of $g_2$ attenuate the K(f) profiles and increase the K-resonant frequency $f_{res,K}$ (Fig. 6-A1 to -C1). Increasing values of $g_c$ attenuate the K(f) profile with no significant change in $f_{res,K}$ (Fig. 6-A1 to -C1). Increasing values of $\tau _2$ shift $f_{res,K}$ to lower values, amplify the K(f) profiles and make the peakier (compare (Figs. 6-A1 and B1). Increasing values of $g_{L,2}$ attenuate the K(f) profiles and make them shallower, with no significant differences in $f_{res,K}$ (compare (Figs. 6-A1 and C1).

Figs. 6-B1 to -D1 show the dependence of the $\Delta \Phi$-profiles on $g_2$, $\tau _2$, $g_{L,2}$ and $g_c$. Increasing values of $g_2$, shift the phasonant frequency $f_{phas}$ to the right and make the changes in the $\Delta \Phi$-profiles more pronounced, particularly for the lower frequencies (Fig. 6-A2 to -C2). For the parameter values in Fig. 6-A and $g_2 = 0.1$, the system exhibits K-resonance, but not $\Delta \Phi$-phasonance (solid blue). Increasing values of $g_c$ decreases the rate of change of the $\Delta \Phi$ profiles. Increasing values of $\tau _2$ shift $f_{phas}$ to lower values (compare (Figs. 6-A2 and B2). Increasing values of $g_{L,2}$ has a similar effect as increasing the values of $g_c$ (compare (Figs. 6-A2 and C2).

### Response to oscillatory inputs: Electrically coupled linearized $\mathbf{I_{Nap}}$ + $\mathbf{I_h}$ and $\mathbf{I_{Nap}}$ + $\mathbf{I_{Ks}}$ cells

We now turn to the analysis of the dependence of the K(f) and $$ \Delta \Phi (f) $$ profiles of biophysically plausible electrically coupled (nonlinear) cells on their biophysical constitutive properties by using the results for the electrically coupled linear resonators discussed above. We primarily focus on the K-resonant and $$ \Delta \Phi $$-phasonant properties. The $$ I_{Nap} $$ + $$I_h$$ and $$ I_{Nap} $$ + $$I_{Ks}$$ models and their dynamics are described in Section 2.3 (see also Appendix A.2 and (Bel et al. 2025), Fig. S4 and S5). The linearized $$ I_{Nap} $$ + $$ I_h$$ and $$ I_{Nap} $$ + $$ I_{Ks}$$ models have the form (1)- (2) with $$ g_L $$ and g ($$g_{L,k}$$ and $$ g_k$$) defined in terms of the biophysical parameters of the corresponding nonlinear models by eqs. (A-9) (Appendix A.3). The linearized parameters are recalculated for each set of biophysical parameters. Linearized models capture certain nonlinear effects present in the biophysical models in which they originate (Rotstein and Nadim 2014) such as the nonlinear amplification that occurs near the threshold for spike generation. This becomes apparent by examining the responses of the linearized models as the biophysical parameters change within some range.

Our results are presented in Figs. 8 and 10 ($$ I_{Nap} $$ + $$I_h$$ model) and Figs. 10 and 11 ( $$ I_{Nap} $$ + $$I_{Ks} $$ model). Fig. 7 shows the results for the $$I_{Nap} $$ model, a reduced model lacking the slower negative feedback currents.

While both $$I_h $$ and $$ I_{Ks} $$ are resonant, $$ I_h $$ is hyperpolarization-activated and depolarizing, whereas $$ I_{Ks} $$ is depolarization-activated and hyperpolarizing (Fig. 20-C). These differences are reflected in the geometry of the phase-plane diagram, which are qualitatively mirror images of each other (Figs. 20-A1 and -B1) (see also Bel et al. (2025), Figs. S4 and S5). The parabolic V-nullclines are concave up for the $$ I_{Nap} $$ + $$I_h$$ model (e.g., Fig. 20-A1) and concave down for the $$ I_{Nap} $$ + $$I_{Ks}$$ model (e.g., Fig. Fig. 20-B1). In previous work (Rotstein and Nadim 2014; Rotstein 2017a), we showed that these differences translate to differences in some of the models’ oscillatory properties. Here we examine the K(f) and $$ \Delta \Phi (f) $$ profiles for the two models when electrically coupled and their dependence on the model parameters, and uncover the differences between the two models particularly in the parabolic-like nonlinear regimes.

The excitability level is determined by a combination of the model parameters as described in Bel et al. (2025). Briefly, for relatively low excitability levels, the fixed-point is relatively far away from the knee of the V-nullcline and the system is in a quasi-linear regime As the excitability level increases, the fixed-point first moves towards the knee of the V-nullcline and the parabolic-like nonlinearities become increasingly dominant, particularly so in a close vicinity of the bifurcation that produces the onset of spikes. This is reflected in the linearized conductances $$ g_L $$ and g ($$g_{L,2}$$ and $$ g_2$$) both explicitly and implicitly through the equilibrium $$ \bar{V} $$. Importantly, changes in $$ G_h $$ or $$ G_q $$ (maximal conductances associated to the resonant currents) explicitly affect both linearized conductances, while changes in $$ G_p $$ (maximal conductance associated to the amplifying current) explicitly affects only the linearized leak conductance $$ g_L $$.

#### Linearized $\mathbf{I_{Nap}}$ model

The $I_{Nap}$ model (Sections 2.3 and Appendix A.2) is a nonlinear 1D model extending the passive cell to include $I_{Nap}$ (nonlinear, instantaneously fast).

The passive cell can be thought of as the linearization of the $I_{Nap}$ model with

30 $$\begin{aligned} g_L = G_L + G_p\, p_{\infty }(\bar{V}) + G_p\, p_{\infty }'(\bar{V})\, (\bar{V} - E_{Na}) \end{aligned}$$

where $G_L$ and $G_p$ are the leak and persistent sodium maximal conductances of the original biophysical model and $\bar{V}$ is the equilibrium voltage in the subthreshold regime (if it exists). The last term in eq. (30) is negative, characterizing the amplifying nature of the $I_{Nap}$ gating variable.

We analyze the dependence of the K- and $\Delta \Phi$-profiles as well as cells’ response profiles on the model parameters $G_L$ and $G_p$ (to the linear level of approximation) by analyzing the dependence of $g_L$ on these parameters both directly and indirectly through the changes they produced on the equilibrium $\bar{V}$.

The dependence of $$ g_L $$ on $$ G_L $$ is straightforward. The dependence of $$ g_L $$ on $$ G_p$$ involves two terms with opposite signs, positive (second) and negative (third). While $$ g_L $$ increases linearly with $$ G_L $$, it decreases with increasing values of $$ G_p $$ (Fig. 7) because of the dominance of the last term over the second one. Geometrically, the dV/dt vs. V curve becomes shallower as $$ G_p $$ increases. Therefore, increasing values of $$ G_p$$ has the same effect on the network response profiles as decreasing values of $$ g_L $$ in Fig. 3. In particular, increasing values of $$ G_p$$ produces the expected amplification of the K-profiles and cause the increase of the $$ \Delta \Phi $$ profiles to be more pronounced.

#### Linearized $\mathbf{I_{Nap}}$ + $\mathbf{I_h}$ model

A summary of our findings is presented in Tables 2 and 3 (middle columns). Briefly, increasing levels of $$ I_{app} $$ cause the model to transition from the quasi-linear to the parabolic-like regimes as the fixed-point moves up along the left branch of the V-nullcline towards its knee. Both $$ g_L $$ and g are decreasing functions of $$ I_{app} $$ (Figs. 8-B and -C) for fixed values of $$ G_h $$ and $$ G_p $$. Increasing values of $$ I_{app} $$ cause an amplification of the K(f) profiles (Figs. 9-A1 and -B1), a decrease in the K-resonant frequencies, and a slight increase in the $$ \Delta \Phi (f) $$ profiles (Figs. 9-A2 and -B2). This is more pronounced for $$ G_p = 0.5 $$ than for $$ G_p = 0.1$$ (compare Figs. 9-A1 and -B1). Both $$ g_L $$ and g are decreasing functions of $$ G_p $$ (Figs. 8-A) and therefore the effects are similar to increasing values of $$ I_{app} $$ (compare Figs. 9-A and -B).

The nonlinear effects become apparent for the largest values of $$ I_{app} $$ and $$ G_p$$ for which the fixed-point of the individual cells is located in a close vicinity of the knee of the parabolic-like nullcline, while for lower values of $$ I_{app} $$ and $$ G_p $$ the system is in a quasi-linear regime. The dependence of the linearized conductances on $$ G_h $$ is more complex. g is an increasing function of $$ G_h$$ for all values of $$ G_p $$ as expected from a resonant conductance (a maximal conductances associated to a resonant current) (Fig. 8-A2). However, the dependence of $$ g_L $$ on $$ G_h $$ switches for some intermediate value of $$ G_p $$.

For the higher values of $$ G_p $$, $$ g_L $$ is a decreasing function of $$ G_h $$ (from light-salmon to blue in Fig. 8-A1), while for the lower values of $$ G_p $$, $$ g_L $$ is a decreasing function of $$ G_h $$. This implies that the K-profile is nonlinearly amplified by increasing values of $$ G_h $$ for the higher values of $$ G_p $$ (Fig. 9-C1, solid), while it is attenuated by increasing values of $$ G_h $$ for the lower values of $$ G_p $$ where the model is quasi-linear (Fig. 9-C1, dashed). Similarly, the K-resonant frequency is a decreasing function of $$ G_h $$ for the higher values of $$ G_p $$ and an increasing function of $$ G_h $$ for the lower values of $$ G_p$$. A qualitatively switch in behavior also occurs for the $$ \Delta \Phi $$-profiles (Fig. 9-C2).

In other words, the presence of $I_{Nap}$ in the nonlinear regime causes the resonant current $I_h$ to amplify the K-profile, while it would otherwise attenuate it in the quasi-linear regime where the levels of $I_{Nap}$ are lower.

#### Linearized $$ I_{Nap} $$ + $$I_{Ks} $$ model

A summary of our findings is presented in Tables 2 and 3 (right columns). Briefly, increasing levels of $I_{app}$ cause the individual neurons to transition from quasi-linear to parabolic-like as the fixed-point moves down along the left branch of the V-nullcline towards its knee. As this happens, $g_L$ decreases for the higher values of $G_p$ (Fig. 10-B1) and increases for the lower values of $G_p$ (Fig. 10)-C1), while g increases for all values of $G_p$ (Fig. 10)-B2 and -C2). This behavior is qualitatively similar for fixed representative values of $G_q$ and $G_p$ although in some cases the dependencies with $I_{app}$ are weak. As $I_{app}$ increases for the higher values of $G_p$, the K-profiles are amplified for the higher values of $G_q$ (Fig. 11-A1, solid) and attenuated for the lower values of $G_q$ (Fig. 11-A1, dashed). In contrast, for the lower values of $G_p$, the K-profiles are attenuated for all values of $G_q$ as $I_{app}$ increases (Fig. 11-B1). In both cases, the K-resonant frequency increases with increasing values of $I_{app}$. A similar type of switch can be observed in the $\Delta \Phi$-profiles although they are minimally affected and the differences are almost imperceptible (Figs. 11-A2 and -B2).

For fixed values of $I_{app}$ and $G_p$, both $g_L$ and g increase with increasing values of $G_q$ (Fig. 10-A to -C). The K-profiles are attenuated and the K-resonant frequency increases with increasing values of $G_q$ (Fig. 11-C1). For fixed values of $I_{app}$ and $G_q$, both $g_L$ and g increase with increasing values of $G_p$ (Fig. 10-A1). As expected, the K-profiles are amplified by increasing values of $G_p$ (Fig. 11-C1).

Note that for certain parameter regimes the $\Delta \Phi (f)$ profile is always positive and therefore the network does not exhibit $\Delta \Phi$-phasonance (e.g, Fig. 11-B2).

#### Linearized models capture the different response properties of the electrically coupled $\mathbf{I_{Nap}}$ + $\mathbf{I_h}$ and $\mathbf{I_{Nap}}$ + $\mathbf{I_{Ks}}$ to oscillatory inputs

Together, the results of the previous two sections highlight the fact that although the two biophysical models involve the interplay of resonant and amplifying currents, there exist significant qualitative differences between the models’ K(f) and $\Delta \Phi (f)$ profiles and their dependence on the model parameters and levels of excitability. As expected, the two biophysical models have a relatively similar dependence on the levels of the participating currents (model parameters) in the quasi-linear regimes where the excitability levels are relatively low and the behavior is dominated by the resonant currents ($I_h$ and $I_{Ks}$). However, this changes significantly when the excitability levels are higher and the models are in a nonlinear, parabolic-like regime in the presence of higher levels of $I_{Nap}$. In these regimes, the synergistic interaction between the resonant and amplifying currents produce unexpected results that cannot be predicted from the quasi-linear behavior nor from the nature of the resonant currents such as the amplifying effects of the resonant current $I_h$. The similarities and differences between the two models are summarized in Tables 2 and 3.

### Electrically coupled two-compartmental models of spatially extended neurons: Effects of asymmetry in the connectivity

Here, we extend our investigation to two-compartment neuron models having asymmetric connectivity and network heterogeneity. More specifically, we consider two-compartment models of linear or linearized cells where the oscillatory input arrives only to one of the compartments (indexed by 1). The coupling coefficient $K(f;g_c/\sigma _2) = g_c/\sigma _2\, \hat{Z}_2^0(f;g_c/\sigma _2)$ and the phase-difference coefficient $\Delta \Phi (f;g_c/\sigma _2) = \hat{\Phi }_2^0(f;g_c/\sigma _2)$ for two-compartment linear models are presented in Section 2.7 where $\hat{Z}_2^0$ and $\hat{\Phi }_2^0$ are the extended impedances of the individual (disconnected) compartments. Both depend on the ratio of $g_c / \sigma _2$ where $g_c$ represents the factor in the coupling strength between the two compartments that is common to both and $\sigma _2$ represents the proportion of the total cell are taken up by compartment 2. Specific expressions for these quantities can be computed from eqs. (26) and (27), respectively, when compartment 2 is passive, and by eqs. (28) and (29), respectively when compartment 2 is a 2D resonator.

Because these quantities depend on the quotient $g_c / \sigma _2$, it is enough to examine $g_c\, \hat{Z}(f;g_c)$ and $\hat{\Phi }(f;g_c)$ for varying values of $g_c$ and arbitrary extended shapes of the impedance and phase profiles. As mentioned above, the dependence of these expressions on $g_c$ consist of a deformation and an additional rescaling by the same quantity ($g_c$) for the firsts one. The results of the previous sections for electrically coupled cells extend to two-compartment models in a straightforward way by performing these operations. They do not affect the relative values of the profiles nor the mechanisms by which they are generated.

The primary effect of increasing $g_c$ on these extended quantities is to attenuate the impedance profile and make it shallower, and to decrease (in absolute value) the phase profile (Bel et al. 2025).

As $g_c$ increases, $g_c\, \hat{Z}(f;g_c)$ is amplified and becomes shallower and $\hat{\Phi }(f;g_c)$ decreases in absolute value. The dependence of these quantities on $\sigma _2$ can be inferred from these results for fixed values of $g_c$ as $\sigma _2$ decreases.

### Nonlinear models: The coupling and phase-difference coefficients depend on the interplay of both the pre- and post-J cells

**Fig. 19:**
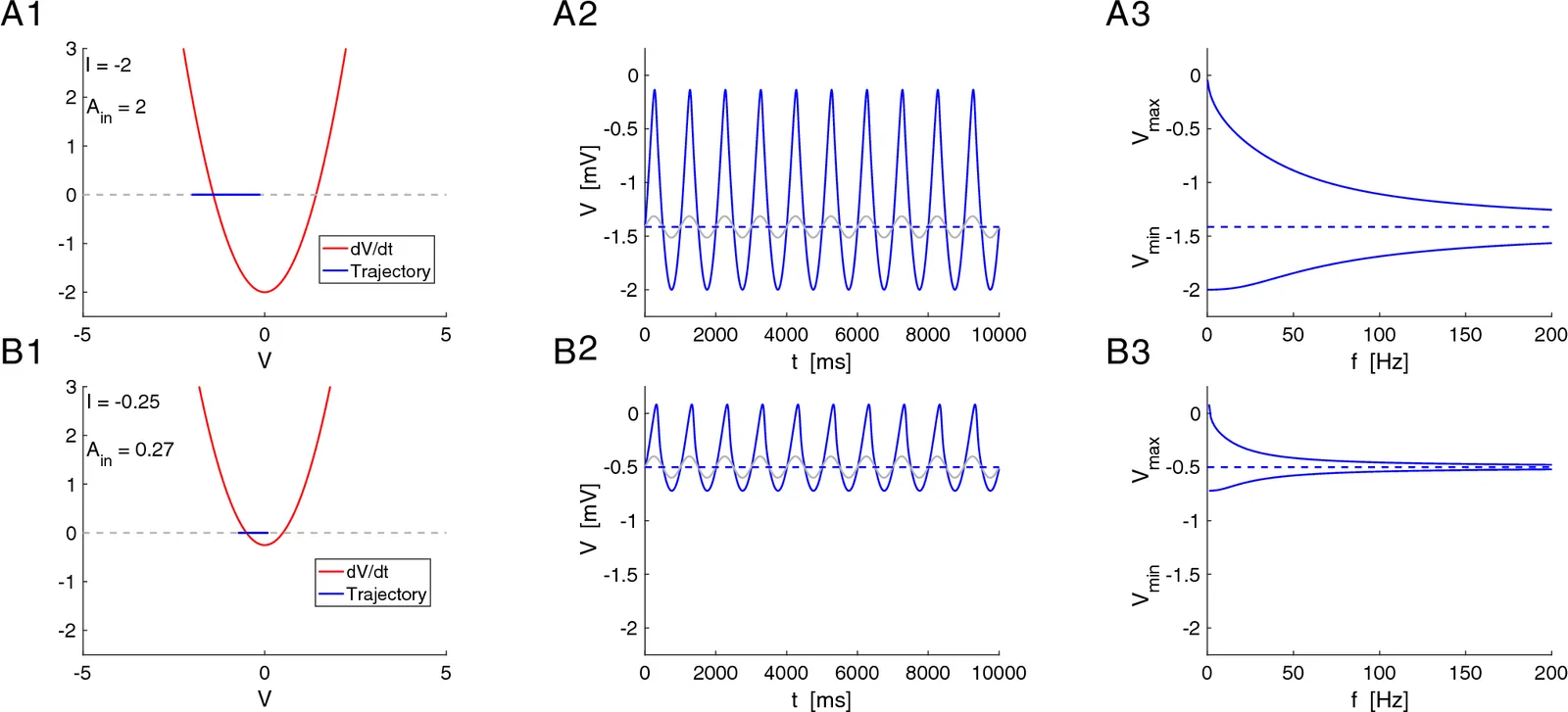
Quadratic 1D model (subthreshold regime) for representative parameter values. We used the model given by eq. (31) with a=1, τ=10 and $g_c = 0$ (isolated cell). **Left column.** Phase-space diagrams: dV/dt vs. V curves (red) and voltage trajectory corresponding to the voltage trace in the middle column (blue). **Middle column.** Voltage trace (blue) in response to an oscillatory input of frequency equal to 1 and amplitude $A_{in}$ (gray; the input signal is rescaled). The value of $A_{in}$ is the threshold value above which v increases unboundedly (escapes the subthreshold regime). **Right column.** Voltage envelope profile in response to oscillatory inputs with the same amplitude as in the left and middle panels

**Fig. 20:**
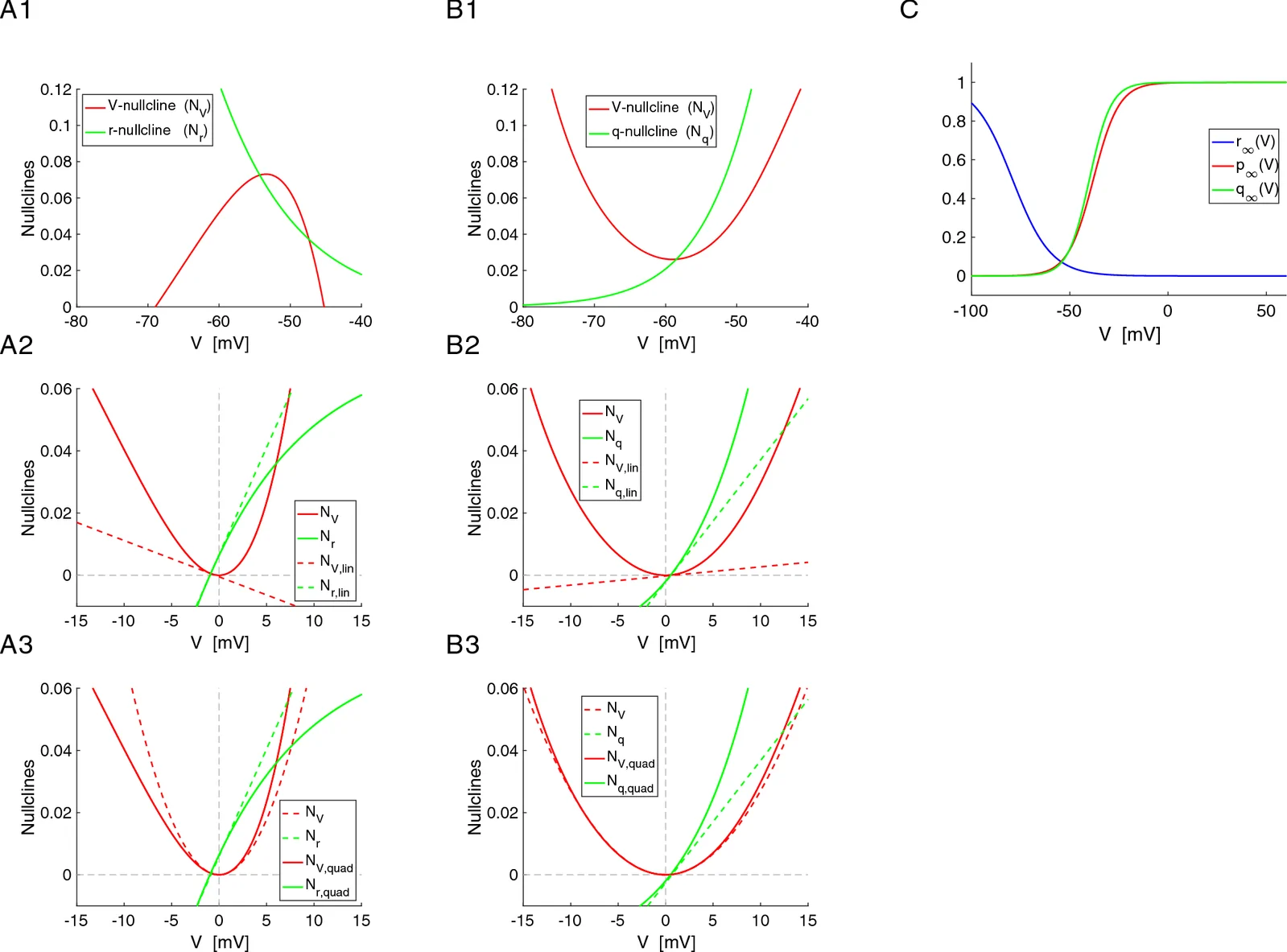
Linearization and quadratization of the $$ I_{Nap} $$+$$I_h$$ and $$ I_{Nap} $$+$$I_{Ks}$$ models with voltage nonlinearities of parabolic type in the subthreshold regime. **A.** $$ I_{Nap} $$+$$I_h$$ model. **B.** $$ I_{Nap} $$+$$I_{Ks}$$. **C.** Activation and inactivation curves for the $$ I_{Nap} $$ + $$ I_h $$ and $$ I_{Nap} $$ + $$I_{Ks} $$ models. We used the parameter values presented in Section A.2. A detailed description of the process for 3D models of HH type including time-dependent external currents and synaptic inputs is presented in Turnquist and Rotstein (2018) and also discussed in Chialva et al. (2023) in the context of reduced neuronal models. We note that while the presence of voltage nonlinearities of parabolic type are a rather general property of models of HH type having regenerative (amplifying) ionic currents (e.g., $$ I_{Nap} $$ or inward-rectifying potassium $$ I_{Kir}$$) (Izhikevich 2006; Rotstein 2017a, b)), in other, also rather general parameter regimes, both models can have V-nullclines of cubic-type (Rotstein 2017a, b; Remme et al. 2012) around which, not around the parabolic component of the cubic-like nullcline relevant subthreshold behavior such as subthreshold oscillations are generated

For linear models, both the K(f) and $\Delta \Phi (f)$ profiles depend on the properties of the post-J cell, and are independent of the properties of the pre-J cell and the input amplitude. Our analytical results for electrically coupled weakly nonlinear cells show that both the pre- and post-J cell and the input amplitude contribute to shaping these two profiles (see Appendix D). Here we investigate in detail how the K(f) and $\Delta \Phi (f)$ profiles are shaped by the intrinsic nonlinearities of quadratic type (Section 2.2; Fig. 3, Fig. 19) in the pre-J and post-J cells and the input amplitude.

We use the quadratic model (3)-(4) where the connectivity parameters are $g_{c,k} = g_c$ for electrically coupled cells mediated by gap junctions and $g_{c,k} = g_c / \sigma _k$ for two-compartmental models. For the 1D reduced model (no negative feedback), we use a slightly modified version of the model, which is described below. The description of the quadratization process linking the parameters of the quadratic models with these of biophysical models with nonlinearities of parabolic-type in the voltage equation is presented in the Appendix A.4 (Rotstein 2015; Turnquist and Rotstein 2018; Chialva et al. 2023).

#### Electrically coupled 1D quadratic cells: interplay of nonlinear LPFs

For cells with 1D quadratic dynamics, the electrically coupled cell model reads

31 $$\begin{aligned} \tau _k\, \frac{dv_k}{dt} = a_k\, v_k^2 + I_k + g_{c,k}\, (v_j - v_k) + I_{in,k}(t) \end{aligned}$$

for k,j=1,2, k≠j where $$ I_k $$ represents the level of excitability, and $$ a_k $$ captures the effect of an instantaneously fast amplifying current (e.g., $$ I_{Nap} $$). As before, the time-dependent oscillatory inputs are given by eq. (5) with $$I_{in,2} = 0 $$.

In the presence of $I_{Nap}$ the cell develops nonlinearities of parabolic-type (Fig. 18). For relatively low levels of $I_{Nap}$, the nonlinearities are away from the equilibrium and therefore the systems is quasi-linear. As the levels of $I_{Nap}$ increase, the equilibrium becomes closer to the knee of the parabolic nullcline and to the unstable fixed-point (who serves as a threshold for spike generation when the model is supplemented with a spiking mechanism). For a critical level of $I_{Nap}$, the two fixed-points collide in a bifurcation and disappear. In the quadratic models, the excitability levels are represented by the DC current $I_k$.

The quadratic 1D model is the subthreshold description of the well-known quadratic integrate-and-fire model (Ermentrout 1996; Latham et al. 2000; Hansel and Mato 2001; Ermentrout and Kopell 1986). Fig. 19 illustrates the dynamics of an individual quadratic cell ($$g_c = 0 $$ and I representing the DC current). As I increases, the speed (dV/dt vs. V) curve increases, the fixed-point moves to the right and the range of values of v for which the response to oscillatory inputs remains in the subthreshold regime shrinks. We choose values of $$ A_{in} $$ so that the response for all input frequencies remains in the subthreshold regime. The effects of the nonlinearities are apparent in the asymmetry, non-sinusoidal shape of the voltage response (compare the response waveforms in Figs. 19-A2 and -B2 and the $$ V_{max} $$ and $$ V_{min} $$ envelope responses in Figs. 19-A3 and -B3).

In Fig. 12 we compare the K(f) and $\Delta \Phi (f)$ profiles for two identical connected cells (blue) and two different connected cells (red) where cell 2 is the same as for the identical pair and cell 1 has the same parameter values as cell 2, except for one that varies across panels. If the model were linear, we would expect the resulting graphs to be superimposed. The differences observed in Fig. 12 indicate that the K(f) and $\Delta \Phi (f)$ profiles are determined by the interplay of both participating cells. These differences are stronger for the K(f) than for the $\Delta \Phi (f)$ profiles. For the K(f) profiles, the differences are stronger for the lower frequencies and decrease as the frequency increases. Because the two cells are network LPFs (not shown) their voltage response range shrinks as the input frequency increases. For the higher frequencies, the response trajectory (e.g., blue line in Fig. 19) reaches the region where the parabolic nonlinearities are prominent. For the lower frequencies, the response trajectory remains in a close vicinity of the fixed point where the dynamics are quasi-linear. These results persists for two-compartment models (not shown).

#### Electrically coupled 2D quadratic cells with negative feedback effects: interplay of nonlinear BPFs, LPFs and time scales

Figures 13 llustrates the nonlinear (amplitude) BPFs (panels A1, C1, D1) and LPFs (panel B1) for the quadratic model for an individual cell for representative parameter values. In addition to resonance, the BPF’s systems exhibit phasonance (panels A2, C2, D2), while the LPF’s system exhibits no phasonance (panel B2). The filters’ nonlinearity is apparent in the lack of symmetry of the peak (upper) and trough (lower) envelopes with respect to the equilibrium (gray dashed horizontal line) and the lack of proportionality with respect to the input amplitude $A_{in}$ (e.g., Fig. 13 -A1, compare the solid-blue response to $A_{in} = 0.05$ and the dashed-blue response for $A_{in} = 0.01$). The effects of the model’s quadratic nonlinearity are reflected in the shapes of the response limit cycles for the resonant frequency (Figs. 13-A3, solid) (Rotstein 2015). For smaller values of the input amplitude $A_{in}$, the response limit cycle remains in a close vicinity of the fixed-point (Figs. 13-A3, dashed) and the peak and trough envelopes are symmetric with respect to the equilibrium (Figs. 13-A1, dashed).

The presence of the model’s nonlinearities does not necessarily imply a strongly nonlinear response such as the one in Fig. 13-A1 (solid) for $$ A_{in} = 0.05 $$. Not only because weakly nonlinear responses (quasi-linear filters) are obtained for lower values of $$ A_{in} $$ that are not necessarily small enough (e.g., Fig. 13-A1, dashed, for $$ A_{in} = 0.01$$), but also because of other factors such as a small enough negative feedback time constant (higher value of ϵ) (e.g., Fig. 13-D1). Comparison between the BPFs in Figs. 13-A1 (solid) and 13-D1 (dashed), for the same value of $$ A_{in} $$ shows that the BPFs are more symmetric and more attenuated, the higher ϵ (Rotstein 2015). This is the result of the interplay of the oscillatory inputs and the underlying vector field (Rotstein 2015) (compare Figs. 13-A3 and -D3). BPFs with a comparable peak value requires a higher value of $$ A_{in} $$ for ϵ=0.1 than for ϵ=0.01. An increase in the parabolic curvature a (Fig. 13-B3) produces a nonlinear response (Fig. 13-B1). However, the filter’s amplitude is smaller as compared to the one for lower values of a (Fig. 13-A1) since the range of voltage values for which the response remains within the subthreshold voltage regime shrinks as a increases.

In our study, we use the parameter values leading to the amplitude BPF in Fig. 13 -A1 as the baseline parameter set (a=0.1, α=0.5 and ϵ=0.01), and we compare a homogeneous network consisting of two identical baseline cells with heterogenous, mixed-cells networks where one cell is a baseline cell and the other has the same parameter values as the baseline cell, except for one. We use the representative parameter values in Figs. 13-B (increase in a, change in the parabolic nonlinearity), 13-C (decrease in α, LPF), and 13-D (increase in ϵ, change in the negative feedback time constant).

**Fig. 21:**
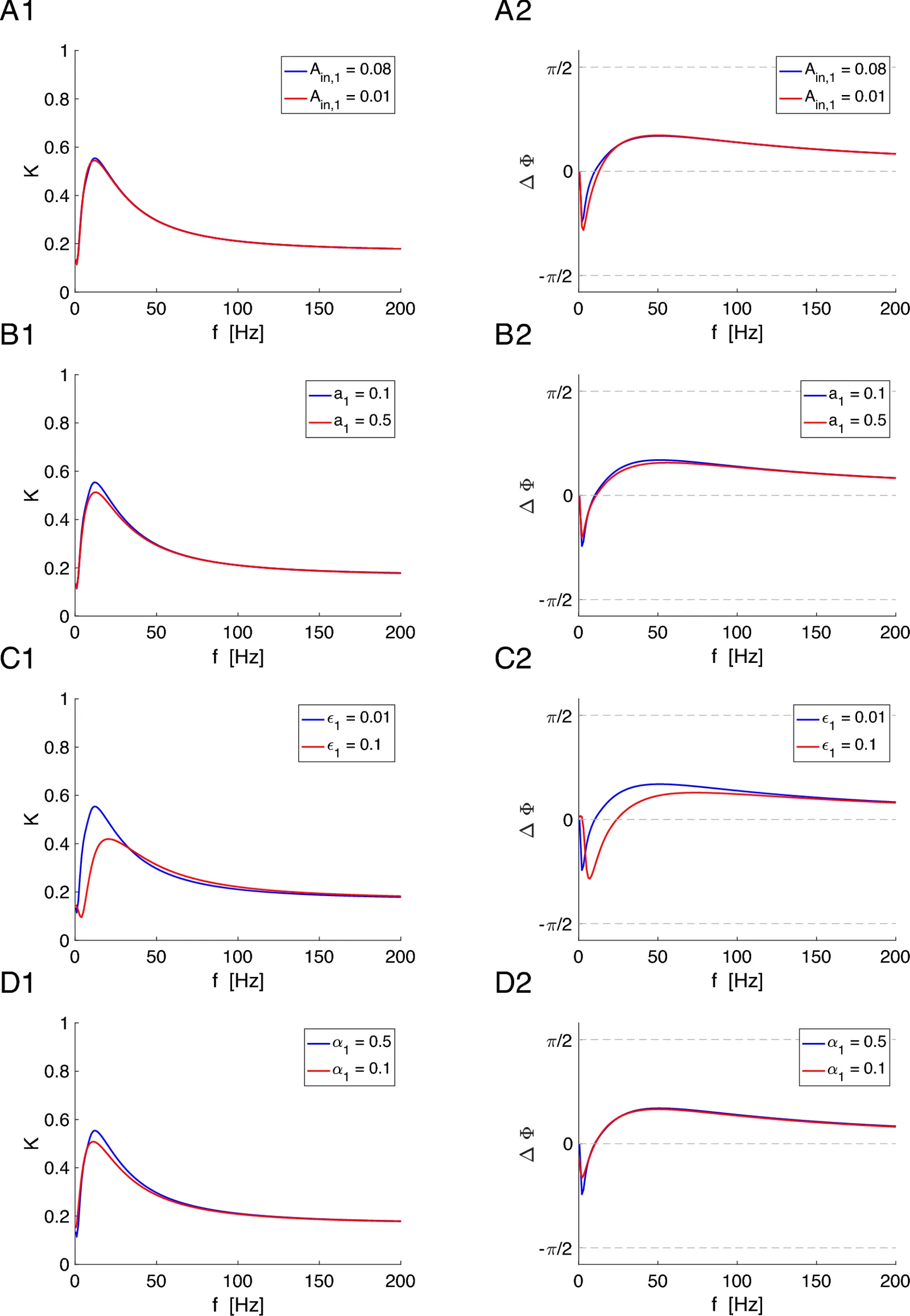
Electrically coupled quadratic 2D model: Effects of the pre-J cell’s properties on the response profiles. We used the quadratic model (3)-(4) for the electrically coupled pre- and post-J cells with quadratic nonlinearities mediated by gap junctions (cell 1 is the pre-J cell; $$ A_{in,2} = 0 $$) . The parameter values for the two cells are identical, except for the ones indicated in the legends. **Left column.** K profiles **Right column.** $$ \Delta \Phi $$ profiles **A.** $$ a_1 = a_2 = 0.1 $$, $$ \alpha _1 = \alpha _2 = 0.5 $$, $$ \epsilon _1 = \epsilon _2 = 0.01 $$. **B.** $$ a_2 = 0.1$$, $$ \alpha _1 = \alpha _2 = 0.5 $$, $$ \epsilon _1 = \epsilon _2 = 0.01 $$, $$ A_{in,1} = 0.08 $$. **C.** $$ a_1 = a_2 = 0.1 $$, $$ \alpha _1 = \alpha _2 = 0.5 $$, $$ \epsilon _2 = 0.01 $$, $$ A_{in,1} = 0.08 $$. **D.** $$ a_1 = a_2 = 0.1 $$, $$ \alpha _2 = 0.5 $$, $$ \epsilon _1 = \epsilon _2 = 0.01 $$, $$ A_{in,1} = 0.08 $$. We used the following additional parameter values: $$ \lambda _1 = \lambda _2 = -0.2 $$, $$ g_c = 0.1 $$ and $$ A_{in,2} = 0 $$

In Fig. 21 we test whether the K(f) and $\Delta \Phi (f)$ profiles depend on the properties of the pre-J cell (Fig. 21-B - D) and the input amplitude $A_{in}$ (Fig. 21-A) in addition to the post-J cell. To this end, we proceed as in the previous section and compare the K(f) and $\Delta \Phi (f)$ profiles of a homogeneous and heterogeneous networks as described above. Our results demonstrate that the K(f) and $\Delta \Phi (f)$ profiles depend on $A_{in}$ and on the properties of the pre-J cell (in addition to the properties of the post-J cell). In some cases, the differences in the profiles between the homogeneous and heterogenous networks is significant, more so than for the quadratic 1D models discussed in the previous section. These differences are more prominent for the lower frequencies and the frequencies in the resonant frequency band where the response amplitude is larger and therefore the response trajectories in the phase-space diagram are able to reach the region of parabolic nonlinearities. For the input frequencies for which the response amplitude is smaller, the trajectories evolve in a closer vicinity of the fixed-point and therefore the dynamics is quasi-linear. In Fig. 13-C we observe that the K(f) profile for the heterogenous network exhibits K-antiresonance in addition of K-resonance, and the $\Delta \Phi (f)$ profile exhibits $\Delta \Phi$-antiphasonance in addition to the $\Delta \Phi$-phasonance. This is the result of a network effect, since neither antiresonance nor antiphasonance are present in any of the participating cells (these phenomena require 3D cellular dynamics).

In Figs. 14 and 15 we extend our results and test the effects of the input location on the K(f) and $\Delta \Phi (f)$ profiles for the heterogeneous networks investigated in Fig. 21. Because in our formulation, the input always arrives to the cell indexed by 1 (the pre-J cell), we switch the parameter values of cells 1 and 2. Specifically, the curves shown in these figures correspond to the homogeneous network of baseline cells (blue), and the two heterogeneous network where cell 2 (cell 1) is a baseline cell and cell 1 (cell 2) has the same parameter values as the baseline cell, except for one, which varies across panels (red and green, respectively). In all three cases in Fig. 14 the K(f) and $\Delta \Phi (f)$ profiles depend on the properties of both the pre-J and post-J cell and are not necessarily dominated by the properties of the post-J cell. This persist for larger values of $g_c$ (Figs. 15) and for two-compartment models where the effects of the compartments’ area renders the connectivity asymmetric (Figs. 16 and 17).

For example, in Figs. 14-A, green, the post-J cell is a LPF and the Φ profile is monotonically increasing, but the K(f) and $\Delta \Phi (f)$ profiles exhibit K-resonance and $\Delta \Phi$-phasonance. On the other hand, in Figs. 14-B (green) the K(f) and $\Delta \Phi (f)$ profiles appear to be dominated by the amplitude and phase profiles of the post-J cell since they are shifted to the right as compared to the other two cases (blue and red). However, the presence of antiresonance and antiphasonance in Figs. 14-B (red) are generated, as mentioned above, by a network mechanism. Similarly, in Figs. 14-C, green, the K(f) and $\Delta \Phi (f)$ profiles are significantly different than the other two cases (blue and red).

**Fig. 22:**
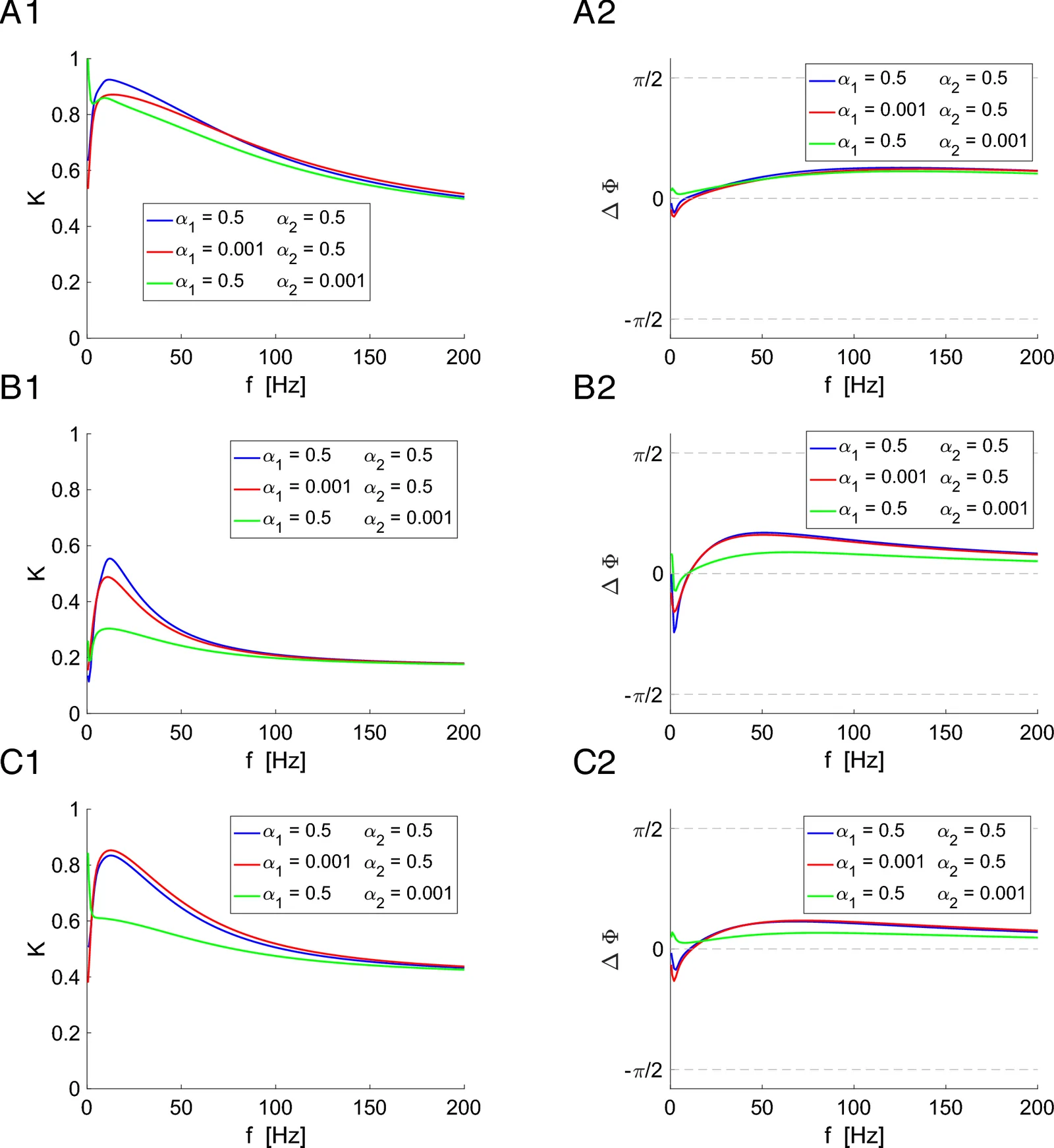
Electrically coupled quadratic 2D model: Effects of the interplay of the biophysical properties of the pre-J and post-J cells and the dendritic geometry on the response profiles. This figure is a rearrangement of Figs. 16-A, 14-A and 17-A to capture the effect of the dendritic geometry for fixed choices of the biophysical parameters. The description of the model is provided in the captions of these figures. We used $a_1 = a_2 = 0.1$, $\epsilon _1 = \epsilon _2 = 0.01$. **A.** Fig. 16-A (σ=0.2). **B.** Fig. 14-A (σ=0.5). **C.** Fig. 17-A (σ=0.8)

**Fig. 23:**
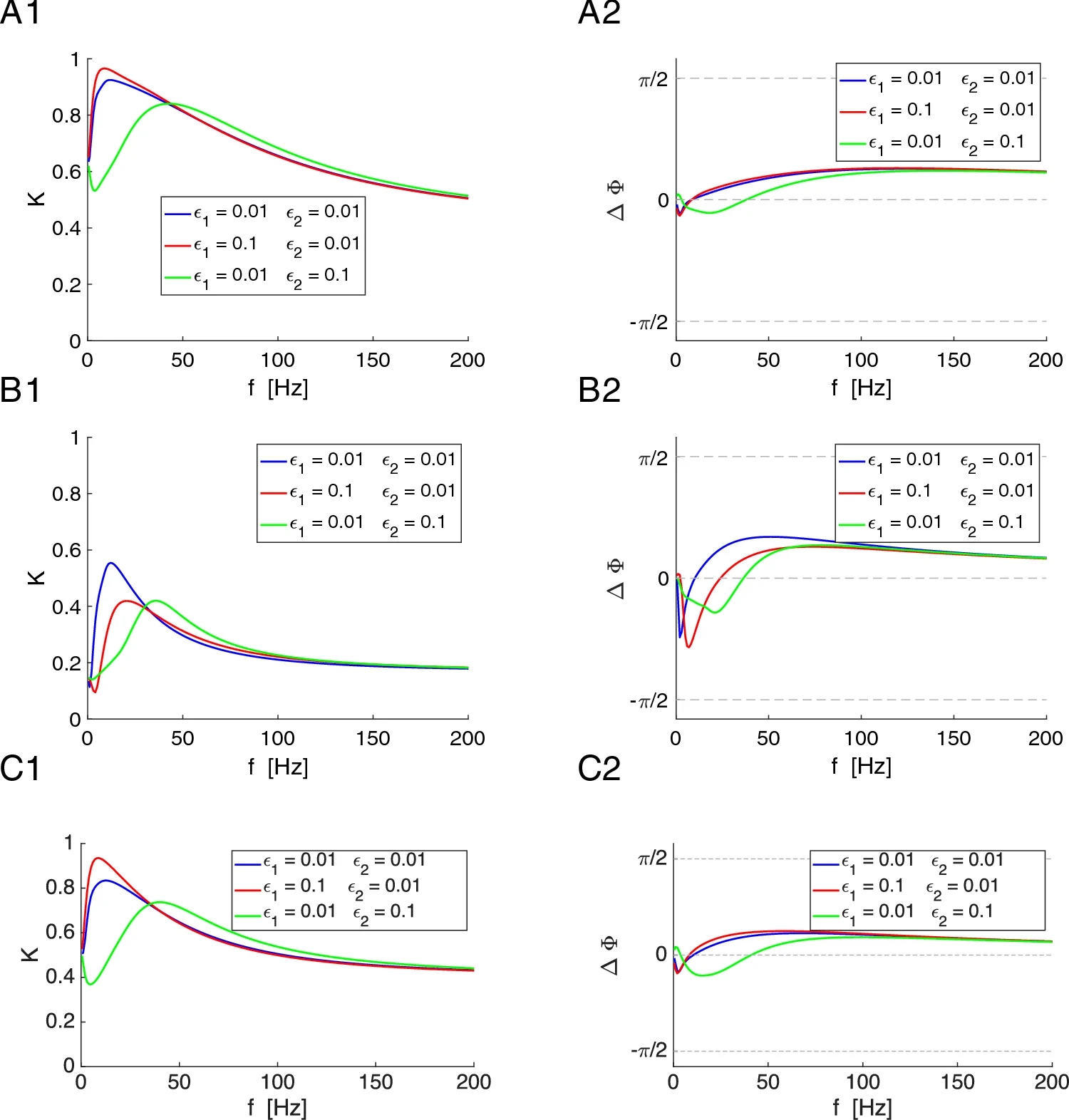
Electrically coupled quadratic 2D model: Effects of the interplay of the biophysical properties of the pre-J and post-J cells and the dendritic geometry on the response profiles. This figure is a rearrangement of Figs. 16-B, 14-B and 17-B to capture the effect of the dendritic geometry for fixed choices of the biophysical parameters. The description of the model is provided in the captions of these figures. We used $a_1 = a_2 = 0.1$, $\alpha _1 = \alpha _2 = 0.5$. **A.** Fig. 16-B (σ=0.2). **B.** Fig. 14-B (σ=0.5). **C.** Fig. 17-B (σ=0.8)

**Fig. 24:**
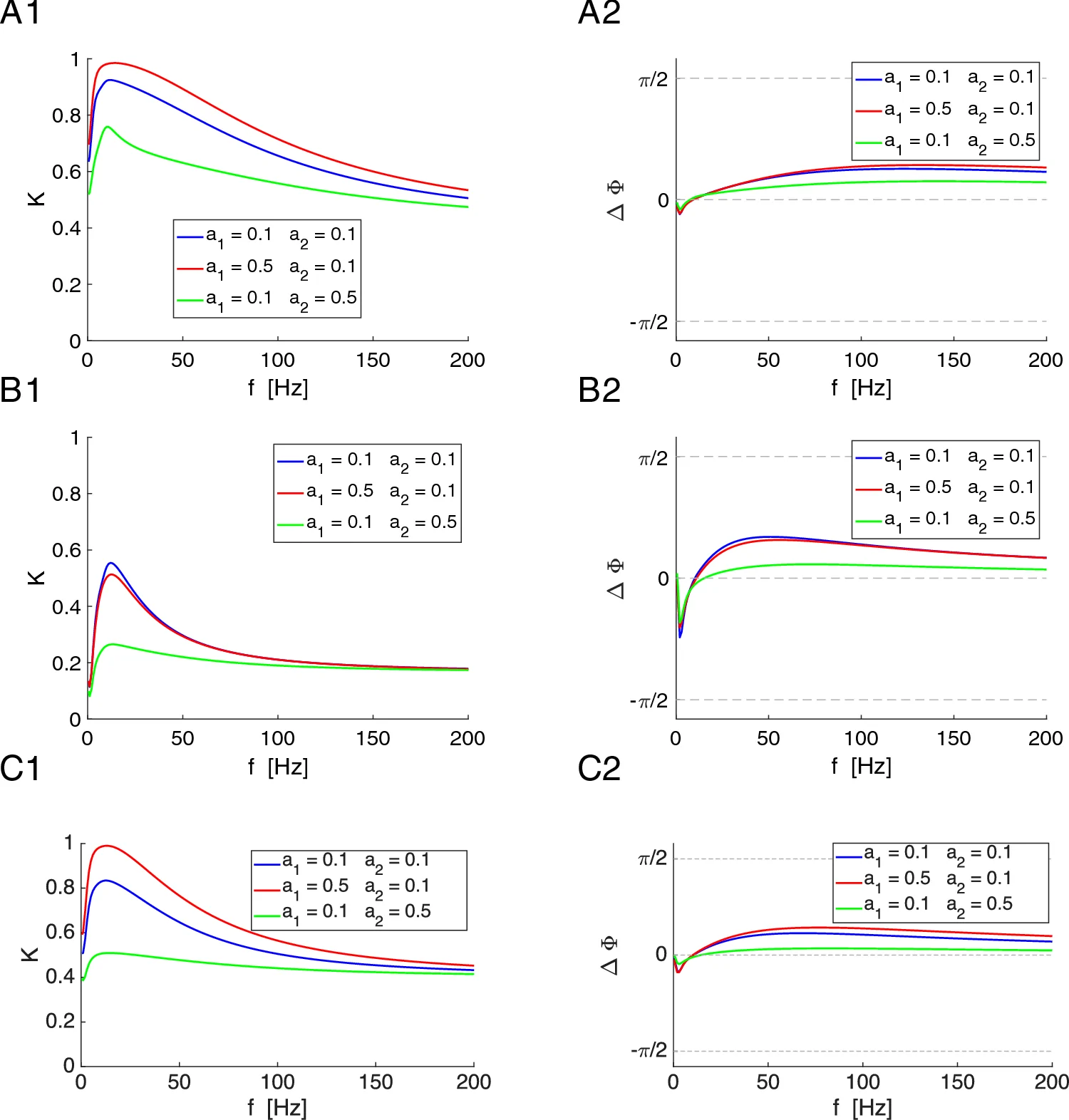
Electrically coupled quadratic 2D model: Effects of the interplay of the biophysical properties of the pre-J and post-J cells and the dendritic geometry on the response profiles. This figure is a rearrangement of Figs. 16-C, 14-C and 17-C to capture the effect of the dendritic geometry for fixed choices of the biophysical parameters. The description of the model is provided in the captions of these figures. We used $\alpha _1 = \alpha _2 = 0.5$, $\epsilon _1 = \epsilon _2 = 0.01$. **A.** Fig. 16-C (σ=0.2). **B.** Fig. 14-C (σ=0.5). **C.** Fig. 17-C (σ=0.8). We used the following additional parameter values:, $\lambda _1 = \lambda _2 = -0.2$, $g_c = 0.05$, $A_{in,1} = 0.06$ and $A_{in,2} = 0$

Comparison among Figs. 14, 16 and 17 provide a picture of how the interplay of the neuronal biophysical properties and dendritic geometry shape the K(f) and $\Delta \Phi (f)$ profiles. To aid in the comparison, we present the rearranged figures in the Appendix E. Figs. 22, 23 and 24 shows a rearrangement of the panels shown in Figs. 14, 16 and 17. Each row in Figs. 22, 23 and 24 correspond to a value of σ for the same fixed set of biophysical parameter values.

## Discussion

The degree to which two nodes in electrically connected networks are coupled is frequency-dependent. This is captured by the K(f) and $\Delta \Phi (f)$ profiles (Curti and O’brien 2016, Galarreta and Hestrin 1999, Curti et al. 2012, García-Perez et al. 2004, Gibson et al. 2005, Curti et al. 2022, Dugué et al. 2009, Curti and Pereda 2004, Davoine et al. 2020, Li et al. 2023), the natural extension of the classical coupling coefficient (CC) (Shimizu and Stopfer 2013, García-Perez et al. 2004, Hoge et al. 2011, Pereda et al. 1828, Gibson et al. 2005, 1977, Bennett 1966, Weizel and Schuster 2019, Carnevale and Johnston 1982, Watanabe and Grundfest 1961, Galarreta and Hestrin 1999, Galarreta and Hestrin 2001, Pernelle et al. 2018, Curti and O’brien 2016, Curti et al. 2022, Hjort et al. 2009, Welzel and Schuster 2019) to account for the response of the pre-J (directly) and post-J (indirectly) cells to oscillatory inputs. Understanding the conditions under which the coupling between pre-J and post-J cells is stronger in amplitude (K-resonance) and tighter in phase ($\Delta \Phi$-phasonance) sheds light into the mechanisms of transmission of information across nodes in electrically coupled networks and the generation of network oscillations.

The K(f) and $\Delta \Phi (f)$ profiles are shaped by the complex interplay of the biophysical and geometric properties of the participating nodes, the connectivity and the oscillatory inputs. The underlying biophysical and dynamic mechanisms have been largely unknown beyond electrically coupled passive cells (but see Curti and O’brien 2016, Curti et al. 2012, Dugué et al. 2009, Curti and Pereda 2004, Davoine et al. 2020, Li et al. 2023). Because they are 1D and linear, their K(f) profiles are LPFs and the $\Delta \Phi$ profiles are positive and monotonically increasing ( Bennett 1977, Bennett 1966, Watanabe 1958, Connors et al. 2010, Connor and Long 2004, Galarreta and Hestrin 1998, Galarreta and Hestrin 1999, Galarreta and Hestrin 2001, Gibson et al. 2005, Landisman et al. 2001, Dugué et al. 2009, Veruki and Hartveit 2002, Shimizu and Stopfer 2013, García-Perez et al. 2004, Pereda et al. 1828, Curti and O’brien 2016, Curti et al. 2022). Moreover, for linear systems, regardless of their complexity and dimensionality, the K(f) and $\Delta \Phi (f)$ profiles are independent of the biophysical properties of the pre-J cell and the input amplitude. How the interplay of cellular intrinsic ionic currents and feedback mechanisms operating in both the pre-J and post-J cells shape the K(f) and $\Delta \Phi (f)$ profiles and under what conditions they exhibit more intricate behavior such as K-resonance and $\Delta \Phi$-phasonance had not been systematically explored.

In this paper we set out to systematically investigate these issues in networks where the pre-J and post-J cells have higher complex dynamics than passive cells. The use of 2D models for the participating cells incorporates the presence of cellular resonance and phasonance and the associated BPFs and mixed leading-lagging phase responses, respectively. The use of nonlinear models incorporates the dependence of the K(f) and $\Delta \Phi (f)$ profiles on the properties of the pre-J cell and the input amplitude in addition to the properties of the post-J cell and the connectivity conductance. We focused on gap junction connected networks and compartmental models of spatially extended neurons, which belong to the same family of electrically coupled network. The connectivity for gap junction networks is symmetric. Asymmetric connectivity arises in two-compartment models with heterogeneous spatial geometry.

We considered minimal network architectures consisting of two electrically connected nodes receiving oscillatory inputs (Fig. 2). The individual nodes (when disconnected) were either LPFs (e.g., passive cells; Fig. 1-A1) or BPFs (e.g., resonators; Fig. 1-A2) and their phase responses were positive (e.g., passive cells; Fig. 1-C1) or mixed negative-positive (e.g., phasonators; Fig. 1-C2). These choices allowed us to systematically investigate the biophysical and dynamic mechanisms that shape the K(f) and $\Delta \Phi (f)$ profiles and determine their preferred coupling frequencies ($f_{res,K}$ and $f_{phas,\Delta \Phi }$) in several representative scenarios where the same or different types of cellular filters interact. While gap junction connected networks typically involve the same type of pre-J and post-J neuron with the same biophysical properties, compartmental models may involve compartments with heterogeneity in their intrinsic properties (Hu et al. 2009; Migliore and Shepherd 2002).

Linear models are amenable to analytical calculations, which we used to understand how the K(f) and $\Delta \Phi (f)$ profiles are shaped by the participating building blocks, negative and positive feedback effects and cellular filtering properties. The K(f) and $\Delta \Phi (f)$ profiles transition to BPFs and mixed leading-lagging responses, respectively, in the presence of an added negative feedback term to the baseline passive cells. The shape of these profiles is modulated by the linearized leak and negative feedback conductances and the negative feedback time constant, which control the corresponding filtering properties of the post-J cell.

Armed with these results and tools, we investigated the dynamics of neurons with biophysically realistic ionic currents to understand, to the linear level of approximation, how the presence and different types of amplifying and resonant ionic processes shape the K(f) and $$ \Delta \Phi (f) $$ profiles. Motivated by experimental results and previous theoretical studies, we used the $$ I_{Nap} $$ + $$ I_h $$ and $$ I_{Nap} $$ + $$ I_{Ks} $$ models (Rotstein and Nadim 2014; Rotstein 2017a, b; Hu et al. 2009) that exhibit both cellular resonance and phasonance (Pike et al. 2000; Zemankovics et al. 2010; Leung and Yu 1998). These two models are representative of the interaction between resonant ($$I_h $$ and $$ I_{Ks} $$) and amplifying ($$I_{Nap}$$) currents. They are biophysically different, because they involve different resonant currents with opposite depolarization properties, but they are dynamically similar, reflected by the fact that their phase-plane diagrams are qualitatively a mirror image of each other (Rotstein 2014). This allowed us to distinguish between biophysical and dynamic effects.

The linearized models we used are linked to the biophysical $I_{Nap}$ + $I_h$ and $I_{Nap}$ + $I_{Ks}$ models: each parameter in the linearized models (linearized parameter) is a function of a set of parameters in the biophysical model (biophysical parameters). The response for each set of linearized parameters is, obviously, linear. However, the dependence of the response of linearized models to changes in the biophysical parameters of the model they represent captures certain aspects of the nonlinear behavior since the nonlinearities are embedded in the linearization process (Rotstein and Nadim 2014). Here, we were interested in processes such as amplification and the modulation of resonance, which materialize when one changes the amplifying and resonant ionic conductances in the biophysical model.

More specifically, in the “linear study" discussed above, we investigated how the K(f) and $\Delta \Phi (f)$ profiles change as we changed the linear parameters independently of any other consideration. In the “linearized" part of the study, in contrast, we changed the biophysical parameters (e.g., ionic conductances) and recalculated the linearized parameters for each set of biophysical parameter values. This process captured some nonlinear effects (to the linear level of approximation) such the qualitative different ways in which the two models ($I_{Nap}$ + $I_h$ and $I_{Nap}$ + $I_{Ks}$) shape the K(f) and Δ(f) profiles and how the preferred frequencies are determined in each case. This is summarized in Tables 2 and 3. One salient difference between the results for the two models is that, in the presence of high enough levels of $I_{Nap}$, increasing levels of $I_h$ amplify the K(f) profile, while increasing values of $I_{Ks}$ attenuate the K(f) profile, while in the presence of low enough levels of $I_{Nap}$, increasing values of both $I_h$ and $I_{Ks}$ attenuate the K(f) profile. A similar effect has been observed for the impedance profile in single cells (Rotstein and Nadim 2014). The effects of asymmetry in the connectivity (e.g., for two-compartment models) was inferred from these results by a set of algebraic operations.

While the linearized models capture certain nonlinear properties of the behavior of the biophysical models, the resulting (“linearized") K(f) and $\Delta \Phi (f)$ profiles remain independent of the properties of the pre-J cells and depend only on the properties of the post-J cell and the connectivity. A set of analytical results using regular perturbation theory for electrically coupled weakly nonlinear cells (Appendix D) showed that as soon as nonlinearities are present, both the pre-J and post-J cells contribute to shaping the K(f) and $\Delta \Phi (f)$ profiles. However, while weakly nonlinear models capture the dynamics of the $I_{Nap}$ + $I_h$ and $I_{Nap}$ + $I_{Ks}$ models for at most low levels of $I_{Nap}$, they fail to do so for the more realistic scenarios with higher levels of $I_{Nap}$ causing the development of voltage nonlinearities of parabolic type in a vicinity of the resting potential. To understand how these nonlinearities affect the properties of the K(f) and $\Delta \Phi (f)$ profiles, we extended our investigation to networks of electrically coupled quadratized cells, which have a parabolic voltage nonlinearities and linear dynamics for the recovery variable. Similarly to linearization, quadratization of biophysical models produces a model (quadratic model) whose parameters (quadratized parameters) are linked to the biophysical parameters of the original, parabolic-like model. In this case, the $I_{Nap}$ + $I_h$ and $I_{Nap}$ + $I_{Ks}$ models.

We analyzed the biophysical mechanisms of generation of the K(f) and Δ(f) in the quadratic models and demonstrated computationally that they depend on the properties of both the pre-J and post-J cells and the network connectivity. In some cases, the contribution of the pre-J cells is relatively small, but in others it is significant, which is missed by using linearized models. Finally, we showed that these results persists for two-compartment models with the additional dependence on the pre-J and post-J geometric properties and for inputs effectively arriving in different compartments. While we limited our study to the effects of the quadratized parameters on the K(f) and Δ(f) profiles, the combination of these results and the results for linearized models discussed above provide a quantitative geometric and biophysical picture of the dependence of the K(f) and Δ(f) on the cellular resonant and amplifying processes. In order to obtain more detailed results, one needs to proceed as for linearized models: recalculate the quadratized parameters for each set of biophysical parameters and compute the K(f) and Δ(f) profiles. We note that in the presence of combinations of constant and oscillatory inputs, the strength of the gap junction is determined by the sum of the stationary and frequency-dependent coupling coefficients. However, the frequency-dependent properties of the gap junction strength are determined only by the latter and independence of the bias DC current. We also note that our definitions of the impedance and peak envelope profiles have been designed to filter possible distortions due to deformations in the response of the quadratic model to sinusoidal inputs. Other types of distortions (e.g., response signals with varying amplitude) have not been observed in the subthreshold voltage regime where instabilities due to the presence of an unstable fixed-point are avoided.

The present study was limited to minimal networks having two nodes (the pre- and post-J cells). However, the ideas and protocols developed in this paper can be extended to electrically coupled networks with a larger number of nodes and possibly multiple inputs, to spatially extended multicompartment neuronal models possibly receiving inputs at different locations (e.g., distal and proximal inhibition by oriens lacunosum-moleculare and fast spiking interneurons, respectively, in hippocampal pyramidal cells), to models having combined amplifying and resonant processes such as $I_{Ca,T}$ activation and inactivation, respectively, and to $I_{Nap}$ + $I_h$ and $I_{Nap}$ + $I_{Ks}$ models having nonlinearities of cubic type, instead of parabolic type, in the subthreshold voltage regime (Rotstein 2014). The present study was also limited to deterministic systems. The presence of noise (e.g., background, ion channel or synaptic noise) would introduce variability in the K(f) and Δ(f) profiles inherited from the variability of the participating cells by mechanisms that involve their transient dynamics (Pena and Rotstein 2022b, a). More research is needed to address these issues and establish the conditions under which the results of this paper persist and to determine the additional phenomena that emerges from the higher dimensionality and complexity of the involved systems and networks. Additional research is needed to understand how the K(f) and Δ(f) profiles relate to experimentally measurable electrical synapse behavior, such as steady- state voltage transfer, signal reliability, timing precision and coincidence detection among others.

Our results advance our knowledge on the response of electrically coupled networks to oscillatory inputs and the frequency-dependent properties of the K(f) and Δ(f) profiles. These results highlight the emergence of preferred coupling frequencies at which K(f) peaks (the coupling strength is maximal at the K-resonant frequency $$ f_{res,K}$$) and for which the pre-J and post-J cell peak synchronously in phase ($$ \Delta \Phi = 0 $$ at the $$ \Delta \Phi $$-phasonant frequency $$ f_{phas,\Delta \Phi }$$). These results have direct implications for spike transmission (Curti et al. 2012; Davoine et al. 2020) in electrically coupled networks in response to oscillatory inputs, for the propagation of signals along the somato-dendritic axis in the presence of resonance and phasonance along the dendritic tree (Hu et al. 2002, 2009; Timofeeva et al. 2013), for the rhythmic coding of hippocampal pyramidal cells (Lowett et al. 2023) and, more generally, for dendritic computation (London and Hausser 2005, Poirazi 2014, Li et al. 2019, Li et al. 2021, Cazé et al. 2013, Cazé et al. 2024). More research is needed to clarify these issues and the difficulties that may emerge in extending this approach to larger networks and spiking regimes (e.g., Haas (2015)) and to test the ideas discussed in this paper by using other measures of effective electrical coupling such as the coupling conductance and the gap junction conductance (Weizel and Schuster 2019). The latter requires oscillatory inputs in voltage clamp. While the impedance and phase profiles for individual linear cells are identical in current and voltage clamp, differences emerge for nonlinear models such as quadratic models (Rotstein and Nadim 2019).

Research is also needed to experimentally test the predictions of our study established by the results discussed along the paper. These include the dependence of the K(f) and Δ(f) profiles on the different types and combinations of resonant and amplifying currents present in both the pre-J and post-J cell (e.g., Tables 2 and 3) and the role of the nonlinearities in shaping the K(f) and Δ(f) profiles. This can be achieved by using the dynamic clamp technique (Prinz et al. 2004; Sharp et al. 1993a, b; Szucs et al. 2017; Kispersky et al. 2010). By creating a real-time, closed-loop interface between an *in vitro* and *in silico* cells, this electrophysiological tool allows for the manipulations of the cellular intrinsic properties of one of the cells in the network.

## Data Availability

The codes will be made available at https://github.com/BioDatanamics-Lab/Coupling_Coefficient-25_05 upon acceptance of the paper

## Biophysical (conductance - based) models of Hodgkin-Huxley type: Linearization and quadratization

### Conductance-based models of single cells

We use the following biophysical (conductance-based) models of Hodgkin-Huxley type (Hodgkin and Huxley 1952; Ermentrout and Terman 2010) to describe the neuronal subthreshold dynamics for each cell

A-1 $$\begin{aligned} C\, \frac{dV}{dt} = - I_L - \sum _{j} I_j + I_{app} + I_{in}(t), \ \ \ \ \ \ \ \ \ \ \end{aligned}$$

A-2 $$\begin{aligned} \frac{dx_j}{dt} = \frac{x_{j,\infty } (V)-x_j }{\tau _{j,x } (V)}, \quad j = 1, 2. \end{aligned}$$

In the current-balance equation (A-1), V is the membrane potential (mV), t is time (ms), C is the membrane capacitance (μF/cm^2^), $$ I_{app} $$ is the applied bias (DC) current (μA/cm^2^), $$ I_L = G_L\, (V - E_L) $$ is the leak current, and $$ I_j = G_{j}\, x_j, (V - E_{j})$$ are generic expressions for ionic currents (with j an index) with maximal conductance $$ G_j $$ (mS/cm^2^) and reversal potentials $$ E_j $$ (mV) respectively. The dynamics of the gating variables $$ x_j $$ obey the kinetic equations (A-2) where $$ x_{j,\infty }(V) $$ and $$ \tau _{j,x}(V) $$ are the voltage-dependent activation/inactivation curves and time constants respectively. For simplicity, the generic ionic currents $$I_j $$ we consider here are restricted to have a single gating variable $$ x_j $$ and to be linear in $$ x_j $$. This is typically the case for persistent sodium ($$I_{Nap}$$), h- (hyperpolarization-activated, mixed-cation, inward; $$ I_h $$), and slow-potassium (M-type) ($$I_{Ks}$$) currents (see Section A.2). The input current $$ I_{in}(t) $$ (μA/cm^2^) in eq. (A-1) has the form (5), $$ I_{in}(t) = A_{in}\, sin(\Omega \, t) $$ with $$ \Omega = 2\, \pi \, f\, /\, 1000 $$ (f is the input frequency in Hz).

Here we focus on 2D models describing the dynamics of V and the gating variable ($x_1$) associated to the ionic current $I_1$. We include an additional current $I_2 = G_2\, x_{2,\infty }(V)\, (V - E_2 )$ by using the adiabatic approximation on the gating variable $x_2$, which is assumed to evolve on a very fast time scale (as compared to the other variables). $I_1$ is assumed to be resonant (restorative, negative feedback) and $I_2$ is assumed to be amplifying (regenerative, positive feedback). Our discussion and results can be easily adapted and generalized to include ionic currents having two gating variables (Richardson et al. 2003; Rotstein 2017, 2018a, b) raised to powers different from one such as T-type calcium and A-type potassium currents (Hutcheon and Yarom 2000). A trajectory exiting the subthreshold regime indicates the onset of a spike. If the model is supplemented with a return mechanism to the subthreshold regime, it becomes a model of quadratic integrate-and-fire type with adaptation (Chialva et al. 2023; Izhikevich 2006, 2010).

### The ${{\textrm{I}}_{\mathrm{{Nap}}}}$ + ${{\textrm{I}}_{\textrm{h}}}$, ${{\textrm{I}}_{\textrm{Nap}}}$ + ${{\textrm{I}}_{\textrm{Ks}}}$ and ${{\textrm{I}}_{\mathrm{{Nap}}}}$ models in the subthreshold regime

The $$I_{Nap} $$ + $$ I_h $$ and $$I_{Nap} $$ + $$ I_{Ks} $$ models (Rotstein 2017a, 2015) are adapted versions of the models presented in Acker et al. (2003); Rotstein et al. (2006) (see also Rotstein (2015, 2017a, 2017b); Turnquist and Rotstein (2018); Chialva et al. (2023)). They involve the interaction between a persistent sodium ($$I_{Nap} = I_2 $$) and either a hyperpolarization -activated (or h-) ($$I_h = I_1 $$) or an M-type slow potassium ($$I_{Ks} = I_1 $$) currents. The formulation of these currents is

A-3 $$\begin{aligned} I_{Nap}&= G_p\, p_{\infty }(V)\, (V - E_{Na}), \quad I_h = G_h\, r\, (V-E_h), \nonumber \\ I_{Ks}&= G_{q}\, q\, (V-E_k). \end{aligned}$$

The activation and inactivation curves are given, respectively, by

A-4 $$\begin{aligned} x_{\infty }(V) = \left( 1 + e^{\frac{V-V_{1/2}}{V_{slp}}}\right) ^{-1} \end{aligned}$$

with (i) $V_{1/2} = -38$ and $V_{slp} = -6.5$ for $p_{\infty }(V)$, (ii) $V_{1/2} = -79.2$ and $V_{slp} = -9.78$ for $r_{\infty }(V)$, and (iii) $V_{1/2} = -40$ and $V_{slp} = 5.5$ for $q_{\infty }(V)$ (Acker et al. 2003). Graphs of these functions are shown in Fig. 20-C (see also Turnquist and Rotstein (2018), Fig. 1). While both $I_h$ and $I_{Ks}$ are resonant, they have activation curves with opposite monotonic properties. The $I_{Nap}$ model is a reduced (1D) version of the other two models.

#### Parameter values

We used the following parameter values (unless stated otherwise)

**Parameter values for the** $$\mathbf{I_{Nap}} $$ + $$ \mathbf{I_h} $$ **model:** C=1, $$ E_L = -65 $$, $$ E_h = -20 $$, $$ E_{Na} = 55 $$, $$ G_L = 0.5 $$, $$ V_{p,1/2} = -38$$, $$ V_{p,slp} = -65 $$, $$ V_{r,1/2} = -79.2 $$, $$ V_{r,slp} = 9.78 $$, $$ \tau _r = 100 $$, $$ G_p = 0.5 $$, $$ G_h = 1.5 $$, $$ I_{app} = -2.5 $$. The values of $$ G_L $$, $$ G_p $$, $$ G_h $$ and $$ I_{app} $$ are the baseline parameter values and were varied in our simulations. For our calculations using the generic model (A-1)-(A-2), we used the following notation: $$ E_1 = E_h $$, $$ E_2 = E_{Na} $$, $$ G_1 = G_h $$, $$ G_2 = G_p $$, $$ V_{1,1/2} = V_{h,1/2} $$, $$ V_{1,slp} = V_{h,slp} $$, $$ V_{2,1/2} = V_{p,1/2} $$, $$ V_{2,slp} = V_{p,slp} $$, $$ \tau _1 = \tau _r $$.

**Parameter values for the** $$\mathbf{I_{Nap}} $$ + $$ \mathbf{I_{Ks}} $$ **model:** C=1, $$ E_L = -54 $$, $$ E_K = -90 $$, $$ E_{Na} = 55 $$, $$ G_L = 0.1 $$, $$ V_{p,1/2} = -38$$, $$ V_{p,slp} = -65 $$, $$ V_{q,1/2} = -40 $$, $$ V_{q,slp} = -5.5 $$, $$ \tau _q = 100 $$, $$ G_p = 0.21 $$, $$ G_q = 1 $$, $$ I_{app} = -0.6 $$. The values of $$ G_L $$, $$ G_p $$, $$ G_q $$ and $$ I_{app} $$ are the baseline parameter values and were varied in our simulations. For our calculations using the generic model (A-1)-(A-2), we used the following notation: $$ E_1 = E_K $$, $$ E_2 = E_{Na} $$, $$ G_1 = G_q $$, $$ G_2 = G_p $$, $$ V_{1,1/2} = V_{q,1/2} $$, $$ V_{1,slp} = V_{q,slp} $$, $$ V_{2,1/2} = V_{p,1/2} $$, $$ V_{2,slp} = V_{p,slp} $$, $$ \tau _1 = \tau _q $$.

**Parameter values for the** $$\mathbf{I_{Nap}} $$ **model:** C=1, $$ E_L = -65 $$, $$ E_{Na} = 55 $$, $$ G_L = 0.1 $$, $$ V_{p,1/2} = -38$$, $$ V_{p,slp} = -65 $$, $$ G_p = 0.1 $$, $$ I_{app} = 0 $$. The values of $$ G_L $$, $$ G_p $$, and $$ I_{app} $$ are the baseline parameter values and were varied in our simulations. Fig. 18 illustrates the dynamics for representative scenarios. For our calculations using the generic model (A-1)-(A-2), we used the following notation: $$ E_2 = E_{Na} $$, $$ G_2 = G_p $$, $$ V_{2,1/2} = V_{p,1/2} $$ and $$ V_{2,slp} = V_{p,slp} $$.

#### Dynamic structure of the $\mathbf{I_{Nap}}$ + $\mathbf{I_h}$ and $\mathbf{I_{Nap}}$ + $\mathbf{I_{Ks}}$ models in the subthreshold regime with nonlinearities of quadratic type

The two models have V-nullclines of quadratic type or are quasi-linear in the subthreshold regime for the parameter sets considered (20-A1 and -B1) (see also Turnquist and Rotstein (2018), Fig. 2, Chialva et al. (2023), Fig. 7, and Bel et al. (2025), Figs. S4 and S5). However, while this is a rather general property of models of HH type having regenerative (amplifying) ionic currents (e.g., $$ I_{Nap} $$ or inward-rectifying potassium $$ I_{Kir}$$) (Izhikevich 2006; Rotstein 2017a, b)), we note that in certain, also rather general parameter regimes, both models can have V-nullclines of cubic-type (Rotstein 2017a, b; Remme et al. 2012) around which, not around the parabolic component of the cubic-like nullcline relevant subthreshold behavior such as subthreshold oscillations are generated.

The $I_{Nap}$ model, which can be thought of as a 1D reduced version of the 2D models discussed above has a parabolic-like dV/dt vs. V curve.

### Linearization: conductance-based linearized models

Linearization of the autonomous part ($$ I_{in}(t) = 0$$) of system (A-1)-(A-2) around the fixed-point $$ (\bar{V},\bar{x}_1) $$ yields (Richardson et al. 2003; Rotstein and Nadim 2014; Rotstein 2018a; Chialva et al. 2023)

A-5 $$\begin{aligned} C\, \frac{dv}{dt} = -g_L v - g_1 w_1 + I_{in}(t), \end{aligned}$$

A-6 $$\begin{aligned} \bar{\tau }_1 \frac{d w_1}{dt} = v - w_1, \end{aligned}$$

where

A-7 $$\begin{aligned} v = V - \bar{V}, \ \ \ \ \ \ \ \ \ \ w_1 = \frac{x_1 - \bar{x}_1}{x_{1,\infty }'(\bar{V})}, \end{aligned}$$

A-8 $$\begin{aligned} \bar{x}_1 = x_{1,\infty }(\bar{V}), \ \ \ \ \ \ \ \ \ \ \bar{\tau }_1 = \tau _{x,1}(\bar{V}), \end{aligned}$$

A-9 $$\begin{aligned} g_L&= G_L + G_1\, x_{1,\infty }(\bar{V}) + G_2\, x_{2,\infty }(\bar{V}) + g_2 \quad \text{ and } \nonumber \\ g_j&= G_j\, x_{j,\infty }'(\bar{V})\, (\bar{V} - E_j), \ \ \ \ \ \ \ \ j = 1, 2, \end{aligned}$$

Note that the gating variables $w_1$ and $w_2$ in (A-7) have units of voltage ($[v] = [w_1]=$V). A Comparison between the original (biophysical) $I_{Nap}$ + $I_h$ and $I_{Nap}$ + $I_{Ks}$ models and their linearization are presented in Figs. 20-A2 and -B2 (see also Chialva et al. (2023), Fig. 7).

The effective leak conductance $g_L$ (A-9) contains information about the biophysical leak conductance $G_L$, the ionic conductances, and their associated voltage-dependent activation / inactivation curves. The fast ionic current $I_2$ contributes to $g_L$ with an additional term $g_2$. The signs of the effective ionic conductances $g_j$ determine whether the associated gating variables are resonant ($g_j > 0$) or amplifying ($g_j < 0$) (Richardson et al. 2003; Hutcheon and Yarom 2000). Specific examples are the gating variables associated to $I_h$ (resonant), $I_{Ks}$ (resonant), $I_{Nap}$ (amplifying) and $I_{Kir}$ (amplifying). All terms in $g_L$ are positive except for the last one that can be either positive or negative. Specifically, $g_L$ can become negative for negative enough values of $g_2$.

All the linearized conductances are affected not only by the respective biophysical conductances, but also by the magnitudes and monotonic properties of the activation and inactivation curves (A-4) and their derivatives (see the examples shown in Section A.2 and Fig. 20-C)

### Quadratization: conductance-based linearized models

Quadratization of biophysically plausible models of HH type (A-1)-(A-2) extends the notion of linearization to include the parabolic-like properties of the V-nullcline in the subthreshold regime and therefore capture more realistic aspects of the dynamics of these models, which can be missed by the corresponding linearization (Rotstein 2015; Turnquist and Rotstein 2018; Chialva et al. 2023). In contrast to linearization, which is carried out around the model’s fixed point (determined by the intersection of the model’s nullclines), quadratization is done around the extremum (minimum or maximum) of the V-nullcline of quadratic type.

One important assumption is that the V-nullcline is parabolic-like in the subthreshold regime (e.g., Fig. 2 in Turnquist and Rotstein (2018) and Fig. 7 in Chialva et al. (2023)). This is a rather general property of neuronal models of HH type having regenerative (amplifying) ionic currents (Izhikevich 2006; Rotstein 2017a, b).

The process of quadratization (Rotstein 2015; Turnquist and Rotstein 2018) consists of expanding the right-side of the model differential equations into Taylor series around the minimum/maximum ($V_e$, $x_{1,e}$) of the parabolic-like V-nullcline in the subthreshold regime, neglecting all the terms with power bigger than two in the equation for V and bigger than one in the equation for $x_1$, and translating the minimum/maximum of the V-nullcline to the origin. Details are provided in Rotstein (2015); Turnquist and Rotstein (2018); Chialva et al. (2023) for 2D and 3D models.

The quadratized 2D model (A-1)-(A-2) around ($V_e$,$x_{1,e}$) is given by

A-10 $$\begin{aligned} \frac{dv}{dt} = \sigma a v^2 - w, \ \ \ \ \ \ \, \end{aligned}$$

A-11 $$\begin{aligned} \frac{dw}{dt} = \epsilon \, [\, \alpha \, v - \lambda - w\, ], \end{aligned}$$

where

A-12 $$\begin{aligned} v&= V - V_e - \frac{g_L}{2\, \sigma \, g_c} \quad \text{ and } \nonumber \\ w&= \frac{g_1}{C}\, \frac{x_1 - x_{1,e}}{x_{1,\infty }'(V_e)} - \frac{F_e}{C} + \frac{g_L^2}{4\, \sigma \, g_c\, C}, \end{aligned}$$

A-13 $$\begin{aligned} g_L&= G_L + G_1\, x_{1,e} + G_2\, x_{2,\infty }(V_e) + g_2, \nonumber \\ g_j&= G_j\, (V_e - E_j)\, x_{j,\infty }'(V_e), \quad j = 1, 2 \ \end{aligned}$$

A-14 $$\begin{aligned} \sigma \, a = - \frac{G_2\, x_{2,\infty }''(V_e)\, (V_e - E_2) + 2\, G_2\, x_{2,\infty }'(V_e)}{2\, C}, \end{aligned}$$

A-15 $$\begin{aligned} \epsilon&= \frac{1}{\tau _1}, \quad \alpha = \frac{g_1}{C}, \nonumber \\ \beta&= \frac{x_{1,\infty }(V_e) - x_{1,e}}{x_{1,\infty }'(V_e)}, \quad \lambda = \frac{F_e}{C} - \frac{g_L^2}{4\, \sigma \, a\, C} - \frac{g_1\, \beta }{C} - \frac{g_1\, g_L}{2\, \sigma \, a\, C}, \end{aligned}$$

and

A-16 $$\begin{aligned} F_e&= F(V_e,x_{1,e}) = I_{app} - G_L\, (V_e - E_L)-G_1\, x_{1,e}\, (V_e - E_1) \nonumber \\&- G_2\, x_{2,\infty }(V_e)\, (V_e-E_2). \end{aligned}$$

A Comparison between the original (biophysical) $$ I_{Nap} $$ + $$ I_h$$ and $$ I_{Nap} $$ + $$ I_{Ks}$$ models and their quadratization is presented in Figs. 20-A3 and -B3 (see also Chialva et al. (2023), Figs. 3 and 4). A detailed description of the process for 3D models of HH type including time-dependent external currents and synaptic inputs is presented in Turnquist and Rotstein (2018) and also discussed in Chialva et al. (2023) in the context of reduced neuronal models.

## Impedance amplitude and phase profiles for 2D linear systems: individual cells

For a linear system

A-17 $$\begin{aligned} C\, \frac{dv}{dt} = -g_{L}\, v - g\, w + I_{in}(t), \end{aligned}$$

A-18 $$\begin{aligned} \tau \, \frac{dw}{dt} = v- w, \end{aligned}$$

receiving a sinusoidal input current of the form (5)

A-19 $$\begin{aligned} I_{in}(t) = A_{in}\, \sin (\Omega \, t) \ \ \ \ \ \ \ \ \ \ \text{ with } \ \ \ \ \ \ \ \ \ \ \Omega = \frac{2 \pi f}{1000}, \end{aligned}$$

the voltage response is given by

A-20 $$\begin{aligned} V_{out}(t;f) = A_{out}(f)\, \sin {(\Omega \, t - \Phi (f))} \end{aligned}$$

where $A_{out}(f)$ is the amplitude and Φ(f) is the phase-shift (or phase), which captures the difference between the peaks of $I_{in}(t)$ and $V_{out}(t;f)$ normalized by the period. For the linear system (A-17)-(A-18), the impedance is given by Richardson et al. (2003); Rotstein and Nadim (2014); Rotstein (2018a))

A-21 $$\begin{aligned} \textbf{Z}(f) = \frac{A_{out}(f)}{A_{in}}= \frac{1+i\, \tau \Omega }{(g_L+i\, C\Omega )(1+i\, \tau \Omega )+g}. \end{aligned}$$

The Z- and Φ- profiles are given, respectively by

A-22 $$\begin{aligned} Z(f) \!=\! C\, \sqrt{\frac{1+\tau ^2\, \Omega ^2}{(g_L + g - C\, \tau \, \Omega ^2)^2 + (g_L\, \tau + C)^2\, \Omega ^2}}, \end{aligned}$$

and

A-23 $$\begin{aligned} \Phi (f) = \tan ^{-1}\left( \frac{C-\tau g +C(\tau \, \Omega )^2}{g_L + g +g_L(\tau \, \Omega )^2 }\Omega \right) . \end{aligned}$$

System (A-17)-(A-18) exhibits *resonance* (BPF) if Z(f) peaks at a non-zero (resonant) frequency $f_{res}$ (Figs. 1-A2) and *phasonance* if Φ(f) vanishes at a non-zero (phasonant) frequency $f_{phas}$ (Figs. 1-C2).

## Network impedance profiles for linear systems: Linear nodes and connectivity

We consider networks consisting of N nodes with 2D linear dynamics and linear connectivity. The dynamics are described by

A-24 $$\begin{aligned} x_k' = a\, x_k + b\, y_k + \sum _{j=1}^{N} \Gamma _{kj}\, x_j+ A_{in,k}\, e^{i\, \Omega \, t}, \end{aligned}$$

A-25 $$\begin{aligned} y _k' = c\, x_k + d\, y_k, \end{aligned}$$

for k=1,…,N. In (A-24)-(A-25), a, b, c and d are constant, Ω>0 is the input frequency and $A_{in,k} \ge 0$ are the input amplitudes. The connectivity coefficients $\Gamma _{kj}$ indicate a connection from cell j to cell k. The coefficients a, b, c and d may be different in different nodes in the network. For simplicity in the notation we omit the subindex, which will be reintroduced later in the notation for the impedance profiles.

We use the notation $\mathbf{Z_k}(\Omega )$, $Z_k(\Omega )$ and $\Phi _k(\Omega )$ (k=1,…,N) to refer to the (complex) impedance, impedance amplitude (or simply, impedance) and phase profiles, respectively, of the individual nodes. The analytical expressions for 2D and 1D (b=0) models are given in the Appendix B.

The particular solution to system (A-24)-(A-25) has the form

A-26 $$\begin{aligned} x_k(t) =\mathbf{A_k}(\Omega )\, e^{i\, \Omega \, t} \ \ \ \text{ and } \ \ \ \ \ \ y_k(t) = \mathbf{B_k}(\Omega )\, e^{i\, \Omega \, t} \ \ \ \ \end{aligned}$$

for k=1,…,N. By substituting (A-26) into (A-24)-(A-25) and rearranging terms we obtain

A-27 $$\begin{aligned} \frac{1}{{\hat{\textbf{Z}}_k}(\Omega )}\, \mathbf{A_k}(\Omega ) - \sum _{j=1,\, j \ne k}^{N} \Gamma _{kj}\, \mathbf{A_j}(\Omega ) = A_{in,k} \end{aligned}$$

for k=1,…,N, where ${\hat{\textbf{Z}}_k}(\Omega )$ is the impedance of the individual linear node k for $\hat{a} = a + \Gamma _{kk}$ (Rotstein and Nadim 2014; Bel et al. 2025), which we refer to as the extended impedance. Similarly, $\hat{Z}_k(\Omega )$ and $\hat{\Phi }_k(\Omega )$ refer to the extended impedance amplitude and phase profiles of these systems, respectively.

This is a complex linear system of algebraic equations that can be written in compact form as

A-28 $$\begin{aligned} \textbf{J}(\Omega )\, \textbf{A}(\Omega ) = A_{in} \end{aligned}$$

whose solution is formally given by

A-29 $$\begin{aligned} \textbf{A}(\Omega ) = \textbf{J}(\Omega )^{-1}\, A_{in}. \end{aligned}$$

The vector $\textbf{A}(\Omega$) represents the response of the network to the oscillatory inputs. For a two-cell network (N=2) we obtain

A-30 $$\begin{aligned} \textbf{A}_1(\Omega )&= \frac{A_{in,1}+ \Gamma _{12}\, {\hat{\textbf{Z}}}_2(\Omega )\, A_{in,2}}{1 - \Gamma _{12}\, \Gamma _{21}\, {\hat{\textbf{Z}}}_1(\Omega )\, {\hat{\textbf{Z}}}_2(\Omega )}\, {\hat{\textbf{Z}}}_1(\Omega ) \quad \text{ and } \nonumber \\ \textbf{A}_2(\Omega )&= \frac{ \Gamma _{21}\, {\hat{\textbf{Z}}}_1(\Omega )\, A_{in,1}+A_{in,2}}{1 - \Gamma _{12}\, \Gamma _{21}\, {\hat{\textbf{Z}}}_1(\Omega )\, {\hat{\textbf{Z}}}_2(\Omega )}\, {\hat{\textbf{Z}}}_2(\Omega ). \end{aligned}$$

More details are provided in Bel et al. (2025).

## Networks of electrically coupled weakly nonlinear cells: Regular perturbation analysis

For electrically connected pairs of linear cells, the coupling coefficients are independent of the properties of the pre-J cell (cell1) and the input amplitude, and depend only on the gap junction coefficient ($g_c$) and the properties of the post-J cell (cell 2). Using regular perturbation analysis for electrically coupled weakly nonlinear cells, we show analytically that as soon as the model becomes nonlinear, the coupling coefficients are affected by the interplay of the intrinsic properties of both the pre- and post-J cells, the input amplitude and $g_c$. As in the main body of the paper, we use the notation K for frequency-independent inputs, and K(f) and $\Delta \Phi (f)$ for frequency-dependent inputs.

We first analyze the response to a constant input to the pre-J cell and then the response to an oscillatory input to the pre-J cell. The coupling coefficients are affected by the intrinsic properties of the pre-J cell (in addition to these of the post-J cell) in both cases. While, in the presence of combinations of constant and oscillatory inputs, the strength of the gap junction is determined by the sum of the stationary and frequency-dependent coupling coefficients, the frequency-dependent properties of the gap junction strength is determined only by the latter.

The networks of electrically coupled weakly nonlinear cells are an extension of the linear model (1)-(2) to include weak cellular nonlinearities in the current balance equation

A-31 $$\begin{aligned} C_k\, \frac{dv_k}{dt} = -g_{L,k}\, v_k - g_{k}\, w_{k} + \epsilon \, F_k(v_k,w_k) + g_c\, (v_j - v_k) + I_{in,k}(t), \end{aligned}$$

A-32 $$\begin{aligned} \tau _{k}\, \frac{dw_{k}}{dt} = v_k - w_{k}, \end{aligned}$$

for k,j=1,2 where $\epsilon \ll 1$. The functions $F_k$ are arbitrary well-behaved functions.

### Response to constant inputs: Stationary coupling coefficient

The steady-state response of the quasi-linear system (A-31)-(A-32) to a constant (DC) inputs solely to cell 1 $I_1$ ($I_2= 0$) can be written as

A-33 $$\begin{aligned} \bar{v}_1 = \bar{v}_{1,0} + \epsilon \, \bar{v}_{1,1} + {{\mathcal {O}}}(\epsilon ^2) \quad \text{ and } \quad \bar{v}_2 = \bar{v}_{2,0} + \epsilon \, \bar{v}_{2,1} + {{\mathcal {O}}}(\epsilon ^2) \end{aligned}$$

where $\bar{v}_{1,0}$ and $\bar{v}_{2,0}$ are given by (20) and (21), respectively. The ${{\mathcal {O}}}(\epsilon )$ approximations are the solutions to

A-34 $$\begin{aligned} \left\{ \begin{array}{lllllllll} (g_{L,1}+g_1+g_c)\ \bar{v}_{1,1} - g_c\, \bar{v}_{2,1} = F(\bar{v}_{1,0},\bar{w}_{1,0}) = \bar{F}_{1,0} \\ \\ -g_c\, \bar{v}_{1,1} + (g_{L,2}+g_2+g_c)\ \bar{v}_{2,1} = F(\bar{v}_{2,0},\bar{w}_{2,0}) = \bar{F}_{2,0} \end{array} \right. \end{aligned}$$

given by

A-35 $$\begin{aligned} \bar{v}_{1,1} = \frac{(g_{L,2}+g_2+g_c)\, \bar{F}_{1,0} + g_c\, \bar{F}_{2,0}}{(g_{L,1}+g_1+g_c)\, (g_{L,2}+g_2+g_c) - g_c^2} \end{aligned}$$

and

A-36 $$\begin{aligned} \bar{v}_{2,1} = \frac{g_c\, \bar{F}_{1,0} + (g_{L,1}+g_1+g_c)\, \bar{F}_{2,0}}{(g_{L,1}+g_1+g_c)\, (g_{L,2}+g_2+g_c) - g_c^2}. \end{aligned}$$

By substituting into (A-33),

A-37 $$\begin{aligned} K = \frac{v_2}{v_1} = \frac{g_c\, I_1 + \epsilon \, [\, g_c\, \bar{F}_{1,0} + (g_{L,1}+g_1+g_c)\, \bar{F}_{2,0}\, ]}{(g_{L,2}+g_2+g_c)\, I_1 + \epsilon \, [\, g_{L,2}+g_2+g_c)\, \bar{F}_{1,0} + g_c\, \bar{F}_{2,0} \,]}. \end{aligned}$$

For linear models (ϵ=0), K is independent of the (constant) input current and the intrinsic properties of the pre-J cell (Curti and O’brien 2016; Curti et al. 2022) (see also Section 3.1.1) and depends only on $$ g_c $$, $$ g_{L,2} $$ and $$ g_2$$. As soon as ϵ>0, K depends also on both the input current and the properties of the pre-J cell ($$ g_{L,1} $$ and $$ g_1 $$).

### Response to oscillatory inputs: Frequency-dependent coupling coefficient

The stationary solution of the quasi-linear system (A-31)-(A-32) to an oscillatory input current $I_1(t) = A_{in}\, e^{i\, \Omega \, t}$ solely to the pre-J cell ($I_2= 0$) can be written as

A-38 $$\begin{aligned} v_k(t) {=} v_{k,0}(t) {+} \epsilon \, v_{k,1}(t) {+} {{\mathcal {O}}}(\epsilon ^2) {=} [\, \textbf{A}_{k,0}(\Omega ) + \epsilon \, \textbf{A}_{k,1}(\Omega )\, ]\, e^{i\, \Omega \, t} {+} {{\mathcal {O}}}(\epsilon ^2) \end{aligned}$$

and

A-39 $$\begin{aligned} w_k(t) {=} w_{k,0}(t) {+} \epsilon \, w_{k,1}(t) {+} {{\mathcal {O}}}(\epsilon ^2) {=} [\, \textbf{B}_{k,0} (\Omega ) {+} \textbf{B}_{k,1}(\Omega )\, ]\, e^{i\, \Omega \, t} {+} {{\mathcal {O}}}(\epsilon ^2) \end{aligned}$$

for k=1,2, where

A-40 $$\begin{aligned} \textbf{A}_{1,0}(\Omega ) = \frac{1}{1 - g_c^2\, {\hat{\textbf{Z}}_1^0}(\Omega )\, {\hat{\textbf{Z}}_2^0}(\Omega )}\, {\hat{\textbf{Z}}_1^0}(\Omega )\, A_{in} \end{aligned}$$

and

A-41 $$\begin{aligned} \textbf{A}_{2,0}(\Omega ) = \frac{g_c\, {\hat{\textbf{Z}}_1^0}(\Omega )}{1 - g_c^2\, {\hat{\textbf{Z}}_1^0}(\Omega )\, {\hat{\textbf{Z}}_2^0}(\Omega )}\, {\hat{\textbf{Z}}_2^0}(\Omega )\, A_{in}, \end{aligned}$$

and ${\hat{\textbf{Z}}_k^0}(\Omega )$, k=1,2 are given by (9)-(11).

The complex coupling coefficient is given by

A-42 $$\begin{aligned} \textbf{K}(\Omega ) = \frac{ \textbf{A}_{2,0}(\Omega ) + \epsilon \, \textbf{A}_{2,1}(\Omega )}{\textbf{A}_{1,0}(\Omega ) + \epsilon \, \textbf{A}_{1,1}(\Omega )} + {{\mathcal {O}}}(\epsilon ^2). \end{aligned}$$

In order to calculate the ${{\mathcal {O}}}(\epsilon )$ terms of $\textbf{K}(\Omega )$, for simplicity, we assume that $F_k$ is independent of $w_k$ (k=1,2) and we expand

A-43 $$\begin{aligned} F_k(v_{k,0}) = \sum _{n=0}^{\infty } \alpha _{k,n}\, v_{k,0}^n = \sum _{n=0}^{\infty } \alpha _{k,n}\, [\textbf{A}_{k,0}(\Omega )]^n\, e^{i\, n\, \Omega \, t}. \end{aligned}$$

The ${{\mathcal {O}}}(\epsilon )$ approximations are the stationary solutions of the following system

A-44 $$\begin{aligned} C_k\, \frac{dv_{k,1}}{dt}&= -g_{L,k}\, v_{k,1} - g_{k}\, w_{k,1} + g_c\, (v_{j,1} - v_{k,1}) \nonumber \\&\quad + \sum _{n=0}^{\infty } \alpha _{k,n}\, [\textbf{A}_{k,0}(\Omega )]^n\, e^{i\, n\, \Omega \, t} , \end{aligned}$$

and

A-45 $$\begin{aligned} \tau _{k}\, \frac{dw_{k,1}}{dt} = v_{k,1} - w_{k,1}, \end{aligned}$$

for k,j=1,2. By solving this system, one obtains

A-46 $$\begin{aligned} v_{k,1}(t) = \sum _{n=0}^{\infty } \textbf{A}_{k,1}(n\, \Omega )\, e^{i\, n\, \Omega \, t} \end{aligned}$$

where

A-47 $$\begin{aligned} \textbf{A}_{1,1}(n\, \Omega ) = \frac{\alpha _{1,n}\, [\textbf{A}_{1,0} (\Omega )]^n + g_c\, \alpha _{2,n}\, [\textbf{A}_{2,0} (\Omega )]^n\, {\hat{\textbf{Z}}_2^0}(n \Omega )}{1 - g_c^2\, {\hat{\textbf{Z}}_1^0}(n \Omega )\, {\hat{\textbf{Z}}_2^0}(n \Omega )}\, {\hat{\textbf{Z}}_1^0}(n \Omega ) \end{aligned}$$

A-48 $$\begin{aligned} \textbf{A}_{2,1}(n\, \Omega ) = \frac{\alpha _{2,n}\, [\textbf{A}_{2,0} (\Omega )]^n + g_c\, \alpha _{1,n}\, [\textbf{A}_{1,0} (\Omega )]^n\, {\hat{\textbf{Z}}_1^0}(n \Omega )}{1 - g_c^2\, {\hat{\textbf{Z}}_1^0}(n \Omega )\, {\hat{\textbf{Z}}_2^0}(n \Omega )}\, {\hat{\textbf{Z}}_2^0}(n \Omega ) \end{aligned}$$

for n=1,2,…. The solution to the constant input term (n=0) is provided in the previous section.

From eqs. (A-38)-(A-39), for linear models (ϵ=0), $\textbf{K}(\Omega )$ is independent of the input amplitude ($A_{in}$) and the intrinsic properties of the pre-J cell as discussed in the main body of the paper, and depends only on $g_c$ and the intrinsic properties of the post-J cell. As soon as ϵ>0, $\textbf{K}(\Omega )$ depends also on $A_{in}$ and the intrinsic properties of the pre-J cell.
